# SDS screen-positive prediction from structured questionnaires in university students: a comparative evaluation of large language model prompting strategies

**DOI:** 10.3389/fpsyt.2026.1942164

**Published:** 2026-10-19

**Authors:** Xiao Yang, Ying Cao, Haibin Wang, Huan Li, Xin Jiang, Huiying Xu, Tao Yao, Xiaolei Zhang, Yekai Chen, Jiaming Li, Bin Zhou, Lingwei Xu, Zhendong Chen, Xinzhong Zhu

**Affiliations:** 1 School of Educational Sciences, Huangshan University, Huangshan, Anhui, China; 2 Faculty of Health Sciences, Universiti Teknologi MARA, Puncak Alam, Selangor, Malaysia; 3 School of Economics and Management, Huangshan University, Huangshan, Anhui, China; 4 College of Education, Zhejiang Normal University, Jinhua, Zhejiang, China; 5 School of Computer Science and Technology, Zhejiang Normal University, Jinhua, Zhejiang, China; 6 Security Department, Zhejiang Normal University, Jinhua, Zhejiang, China; 7 School of Psychology, Zhejiang Normal University, Jinhua, Zhejiang, China; 8 College of Mathematical Medicine, Zhejiang Normal University, Jinhua, Zhejiang, China; 9 AIFORIA (Hangzhou) Intelligent Technology Co., Ltd, Hangzhou, Zhejiang, China

**Keywords:** calibration, large language models, repeated-output stability, SDS screen-positive prediction, structured questionnaires, threshold sensitivity

## Abstract

**Introduction:**

Large language models (LLMs) can render structured questionnaire variables as natural-language profiles, but their value in mental-health screening cannot be judged from a single fixed-threshold score. We evaluated LLM prompt conditions in a cross-questionnaire benchmark for predicting screen-positive status on the Self-Rating Depression Scale (SDS) among undergraduates. The intended application was an offline methodological benchmark using previously collected questionnaire data, not a shorter alternative to direct SDS administration.

**Methods:**

De-identified records were assigned to Development (n=1,951), Test (n=2,186), and Holdout (n=2,258) grade cohorts. We evaluated logistic regression, XGBoost, and 12 model-prompt conditions at the fixed 0.5 threshold and at thresholds selected only in Development-validation. We also examined paired bootstrap differences, ranking stability, scale correlations, grouped ablations, cohort shift, calibration intercepts and slopes, alternative expected calibration error specifications, representative repeated calls at temperatures 0 and 0.1, sex-stratified performance, and per-1,000 screening scenarios.

**Results:**

At the fixed 0.5 threshold on Test, GPT E1, Gemini E2, and XGBoost achieved macro-F1 values of 0.732, 0.730, and 0.674. At Development-derived thresholds of 0.38, 0.50, and 0.174, the corresponding macro-F1 values were 0.702, 0.730, and 0.660. At the Development-selected specificity target, XGBoost achieved a Test macro-F1 of 0.738. Pairwise intervals showed no clear fixed-0.5 macro-F1 difference among the three highest-ranked LLM conditions. XGBoost yielded the lowest Brier scores. The combined-sample Spearman correlation between SDS and SAS was 0.60. Together with the ablation results, this correlation indicated predictive information distributed across overlapping questionnaire groups rather than dependence on a single measured group. In repeated calls across the complete Holdout cohort, inconsistent-case rates were 23.6% for GPT E1 and 1.5% for Gemini E2. Temperature 0 did not clearly eliminate GPT E1 instability in the representative 400-case subset.

**Discussion:**

Model conclusions depended on both the threshold and the metric. Certain LLM prompt conditions were competitive at specific operating points, but the evidence did not establish a stable or general advantage over structured-data models. Nor did it establish external validity, clinical diagnostic utility, or reduced questionnaire burden. Threshold choice, calibration, output validity, and repeated-call stability therefore require separate evaluation before prospective use.

## 1 Introduction

University students encounter academic, interpersonal, and developmental transitions during which psychological distress may become more apparent (1, 2). International surveys and syntheses document substantial mental-health needs among university students, whereas multinational programs support surveillance (3–8). In the Huangshan University data, the proportion reaching the prespecified SDS screen-positive threshold ranged from 16.3% to 27.9% across grade cohorts. Although these values do not estimate population prevalence, they illustrate the magnitude and heterogeneity of the local screening problem. Screen-positive results may identify students who require further assessment, but they cannot establish a psychiatric diagnosis (9).

The 20-item Zung Self-Rating Depression Scale (SDS) provides a standardized self-report measure of depressive symptom burden (10). We defined screen-positive status as an SDS standard score of at least 53, a threshold used in Chinese SDS applications (11, 12). The outcome was therefore a questionnaire-based screening threshold, not depressive disorder confirmed by clinical interview. Predictors comprised sex, age, the Self-Rating Anxiety Scale (SAS), Cognitive Fusion Questionnaire (CFQ), Acceptance and Action Questionnaire-II (AAQ-II), and eligible non-depression dimensions of the SCL-90 (13–16). Psychometric evidence is available for the SDS across Chinese populations (17). Related Chinese studies evaluated the SAS in university students (18), the 9-item CFQ in a general sample (19), the AAQ-II in college students and athletes (20), and the SCL-90 in undergraduates (21). We excluded SDS, the SCL-90 depression subscale, and highly aggregated SCL-90 summaries. Nevertheless, conceptual overlap among anxiety, general distress, and depressive symptoms remained a validity concern that required direct analysis.

Structured questionnaire variables can be modeled directly with logistic regression and XGBoost (22–24). In screening applications, however, applying a common cutoff can be misleading because scores from different models need not have the same probabilistic meaning. Discrimination, calibration, and clinically relevant operating points should therefore be evaluated separately. Thresholds should also be selected without using evaluation outcomes (25–27). These requirements are especially important when model scores are used to prioritize cases for review.

Natural-language profiles of structured variables introduce representation and prompting choices beyond direct matrix-based modeling (28–32). These choices differ across structured-data, table-reasoning, and biomedical tasks (33–35). Evaluation should therefore assess calibration, structured-output validity, and repeatability alongside macro-F1 (36–39). Reporting and appraisal guidance for health-related AI therefore emphasizes transparent task boundaries, data roles, model versions, output handling, and uncertainty rather than a single accuracy statistic (40–45). The key evidence gap is not simply which current LLM ranks first. It is whether comparative conclusions remain credible across thresholds, calibration specifications, cohort shifts, construct-overlap checks, and repeated calls.

Previous LLM studies of mental health have addressed substantively different data and tasks. Xu et al. used social-media language to detect suicide risk, whereas Teferra et al. used DAIC-WOZ clinical-interview transcripts to estimate PHQ-8 item scores for automated depression screening (46, 47). Social-media text, clinical-interview language, and consented structured questionnaire profiles differ in provenance, context, task definition, privacy expectations, and the consequences of false classification. Their performance estimates are therefore not directly comparable with those of the present SDS-threshold task. We instead compared prompting strategies using a fixed inventory of structured questionnaire variables rendered as text. We then tested whether the comparisons remained credible under broader robustness analyses.

Several psychological questionnaires are required to construct the predictor profile. The approach therefore does not reduce questionnaire burden relative to direct administration of the 20-item SDS and is not proposed as an SDS replacement. The analysis tests whether previously collected non-SDS questionnaire data can support an auditable offline threshold-prediction benchmark. It also identifies failure modes that must be addressed before any prospective workflow study. Simple scoring rules and conventional machine-learning models therefore remain essential comparators. Using an LLM for this structured-input task adds representation and generation steps that conventional tabular models do not require. A prospective rationale is that the same language interface could eventually combine questionnaire summaries with conversational or other unstructured information, although the present study evaluated only the structured-questionnaire component.

We compared logistic regression, XGBoost, and three LLMs under four prompt conditions, yielding 12 LLM conditions. Supplementary analyses selected model-specific thresholds exclusively in Development-validation and evaluated fixed-sensitivity and fixed-specificity operating points. We also quantified paired model uncertainty, scale overlap, grouped ablation and demographics-only performance, cohort distribution shift, enhanced calibration, repeated-output stability at two temperatures, sex-stratified performance, and per-1,000 screening consequences. The aim was to characterize validity, robustness, and applicability boundaries, not to establish comprehensive LLM superiority or clinical diagnostic utility.

## 2 Materials and methods

### 2.1 Study overview

Before the primary comparison, the core workflow fixed the data roles, predictors, prompts, model slugs, and calling settings. The supplementary-analysis protocol was frozen before any supplementary results were generated. Its configuration, seed, and input hashes were retained. Traditional machine learning received structured feature matrices, whereas LLMs received natural-language profiles rendered from the same primary predictor inventory.

**Figure 1** summarizes the fixed cohort roles and analytical workflow. First-year students formed Development, second-year students formed Test, and third-year students formed Holdout. Development was divided into training and validation subsets with distinct analytical roles. Training supported ML fitting and frozen exemplar selection, whereas validation supported XGBoost calibration and model-specific threshold selection. Test compared 12 LLM prompt conditions with LR and XGBoost at the prespecified 0.5 threshold. The Test-selected finalists, GPT E1 and Gemini E2, were then evaluated with both ML comparators in Holdout. Holdout represented independent within-institution grade-cohort validation, not external or temporal validation. Supplementary analyses examined thresholds and paired uncertainty, construct overlap and cohort shift, calibration, stability, subgroup performance, and per-1,000 screening consequences.

**Figure 1:**
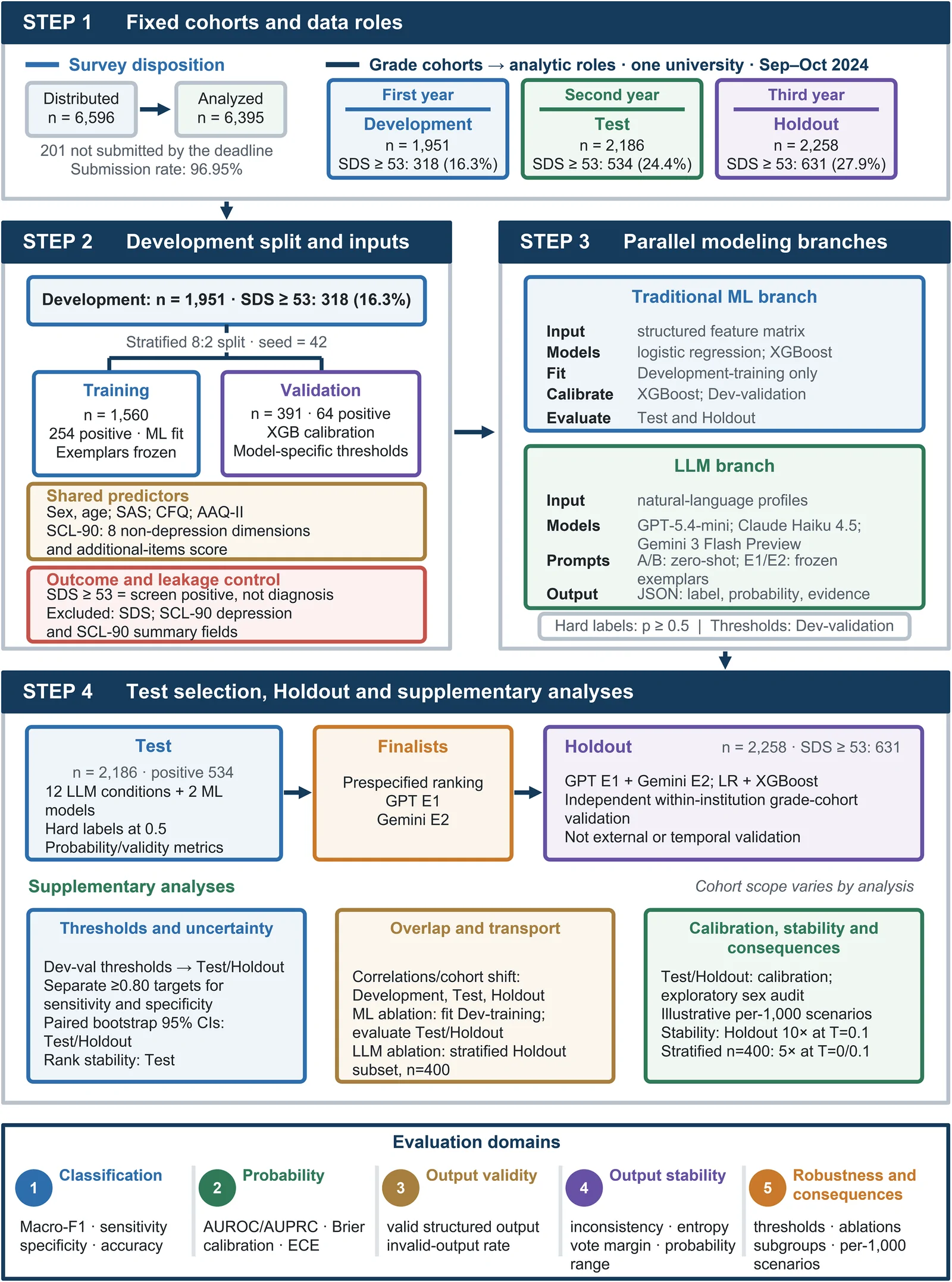
Study design and analytical workflow. Three grade-specific questionnaire cohorts served as Development, Test, and independent within-institution Holdout cohorts. Development supplied ML training, frozen exemplars, calibration-method selection, and model-specific thresholds. Test compared 12 LLM prompt conditions with two structured-data models, after which the Test-selected finalists were evaluated on Holdout. Supplementary analyses examined threshold curves and operating points, paired bootstrap differences, scale correlations and ablations, cohort shift, and enhanced calibration. They also assessed representative stability at two temperatures, sex subgroups, and per-1,000 screening scenarios.

**Supplementary Table S1** reports the frozen run configuration and request audit. **Supplementary Table S2** provides complete single-run estimates, and **Supplementary Table S3** reports the primary repeated-output stability estimates. **Supplementary Table S4** contains the exact model and prompt specifications, frozen exemplars, parsing and retry rules, and file hashes. **Supplementary Table S5** provides privacy-preserving synthetic input/output examples. **Supplementary Tables S6**-**S13** report supplementary thresholds and fixed operating points, paired comparisons and ranking, construct-overlap and demographics-only analyses, cohort shift, calibration, stability, subgroup results, and per-1,000 scenarios.

### 2.2 Data source, sample roles, and fixed splits

This study used de-identified structured mental-health questionnaire data from first- to third-year undergraduates at Huangshan University. The survey was conducted from September to October 2024 through the university’s institutional psychological-assessment system. The target population comprised all first- to third-year undergraduates registered in that system during the survey period. Huangshan University was selected because its established institution-wide system provided a complete and consistently administered survey frame across the three grade cohorts. Recruitment was census-based rather than probability or discipline-proportional sampling: all 6,596 eligible students in the frame were invited, and no separate sample-size calculation was performed. Analytic inclusion required current enrollment in years 1–3, electronic informed consent, and an on-time successfully submitted questionnaire record. Of the 6,596 invited students, 6,395 submitted by the deadline and entered the analytic dataset, giving a submission rate of 96.95%; the remaining 201 did not submit on time and were excluded. No additional respondents were excluded after the prespecified data-integrity checks, and discipline was not retained in the analytical export. The survey comprised 146 items: the 20-item SDS, 20-item SAS, 9-item CFQ, 7-item AAQ-II, and 90-item SCL-90 (17–21). After excluding the 20 SDS items used to define the outcome, 126 non-SDS items contributed to the predictor scales. Completion time was unavailable in the institutional dataset; the 146-item battery was therefore treated as a substantial multi-instrument assessment rather than a brief screen. The analysis used de-identified scale and dimension scores generated within the institutional system, whereas item-level questionnaire responses were unavailable in the analytical dataset. Questionnaire item text was neither transmitted to the LLMs nor reproduced in the manuscript or **Supplementary Materials**. SDS defined the outcome only. SAS, CFQ, AAQ-II, and eligible non-depression SCL-90 variables were candidate predictors. SDS, SCL-90 depression, and SCL-90 summary statistics were excluded from model inputs. The first-, second-, and third-year cohorts were assigned to Development, Test, and Holdout, respectively.

Sample size, sex distribution, age, SDS≥53 positive cases, and the internal Development split are reported in **Table 1**.

**Table 1:** Dataset characteristics and internal development split.

Analysis set	n	Female, n	Male, n	Age, mean (SD)	SDS≥53, n (%)
Development	1,951	1,063	888	19.34 (1.23)	318 (16.3)
Development-training	1,560	858	702	19.34 (1.24)	254 (16.3)
Development-validation	391	205	186	19.32 (1.17)	64 (16.4)
Test	2,186	1,125	1,061	20.40 (0.96)	534 (24.4)
Holdout	2,258	1,136	1,122	21.49 (0.99)	631 (27.9)

Development was used only for training and validation. Prespecified processing mapped source columns to standardized fields, rejected unmapped columns, and converted direct identifiers to 12-character SHA-256 hashes. It also standardized sex, converted questionnaire variables to numeric values, and checked duplicates, cross-split overlap, missingness, and theoretical ranges. The SDS≥53 label was derived from the recorded SDS standard score. Using seed 42, Development was stratified by this label into Development-training (n=1,560) and Development-validation (n=391). The former trained LR and XGBoost and supplied the frozen exemplars. The latter selected XGBoost calibration and all supplementary model-specific thresholds. Test and Holdout labels were never used for fitting, calibration selection, or threshold selection.

Test evaluated all 12 LLM conditions and both machine-learning models and supported the prespecified finalist selection. Holdout evaluated only the two Test-selected LLM finalists and the structured-data comparators. All three cohorts came from the same university and survey period and differed mainly by year level. We therefore describe Holdout as an independent within-institution cohort validation, not as external or temporal validation.

The SDS≥53 rates were 16.3% in Development, 24.4% in Test, and 27.9% in Holdout. We treated these differences as cohort heterogeneity rather than random-split fluctuation. Distribution-shift analyses therefore compared predictor distributions and outcome prevalence across every pair of cohorts.

### 2.3 Outcome definition, de-identification, and predictor inventory

The primary-analysis outcome was the screen-positive label defined by an SDS standard score ≥53. For student *i*, the label was coded as 1 if the SDS standard score was ≥53. Otherwise, it was coded as 0. This label was a threshold-based screening label. It did not represent a clinical diagnosis of depression and was not used for etiological interpretation or treatment recommendations. Accordingly, we treated the task as a cross-questionnaire SDS-threshold prediction benchmark and did not interpret the observed associations as evidence of independent or etiological effects.

Raw identifiers were standardized and hashed as subject_id_hash. Direct identifiers were not retained in model inputs, and the study reported only aggregate metrics and cohort-level characteristics.

The primary predictor inventory was identical across modeling branches: sex, age_years, sas_std, cfq_total, aaq2_total, and eight non-depression SCL-90 symptom dimensions plus the additional-items score. **Table 2** lists these fields and their leakage-control categories. For grouped analyses, sex and age formed a demographics group that was always retained. SAS, CFQ, AAQ-II, and all eligible SCL-90 fields formed four prespecified questionnaire groups.

**Table 2:** Predictor inventory and leakage-control categories used in the primary analysis.

Variable group	Kept fields	ML expression	LLM expression
Demographic	sex, age_years	Sex one-hot; age_years continuous with preprocessing	Sex text; rounded integer age_years
Anxiety scale	sas_std	Continuous	Natural-language numeric
Cognitive fusion	cfq_total	Continuous	Natural-language numeric
Experiential avoidance	aaq2_total	Continuous	Natural-language numeric
SCL-90 factors	scl90_somatization, scl90_obsessive_compulsive, scl90_interpersonal_sensitivity, scl90_anxiety, scl90_hostility, scl90_phobic_anxiety, scl90_paranoid_ideation, scl90_psychoticism, scl90_additional_items	Continuous	Natural-language numeric
Supplemental SCL-90 fields	scl90_depression, scl90_mean, scl90_positive_count, scl90_positive_mean	Optional supplemental-predictor export only; not used in primary models	Optional supplemental-predictor export only; not shown in primary participant profiles
Key label-defining or leakage-control excluded fields	sds_std, scl90_total, scl90_negative_count	Not used as primary model inputs	Not shown in primary participant profiles

After cleaning, no split had missing values in the primary predictor inventory. LLM profiles were generated from complete predictor records without additional missing-value handling. Age was included in the same predictor inventory in both branches, but it was expressed with slightly different precision. The ML branch used continuous age_years. For readability in natural-language descriptions, the LLM profiles used rounded integer values of age_years.

All primary inputs excluded SDS and fields closely proximate to the target. Nevertheless, SAS and the eligible non-depression SCL-90 fields measure symptoms that may covary with depressive symptom burden. Leakage controls therefore did not eliminate construct overlap. Scale-level Spearman correlations and grouped leave-one-out and single-group analyses were used to quantify this overlap. These analyses were interpreted as measures of predictive dependence rather than causal contribution.

The aggregate fields scl90_total and scl90_negative_count were also excluded from primary-analysis inputs. This design reduced the risk of approximately reconstructing the target from highly aggregated symptom-burden statistics. It also kept the primary predictor inventory aligned across the ML and LLM branches. The branches nevertheless differed in modeling. LR and XGBoost underwent cross-validated hyperparameter selection and fitting in Development-training. XGBoost calibration methods were selected and fitted in Development-validation. LLMs received no parameter updates and used fixed slugs, prompts, and calling parameters.

### 2.4 Prompt families and frozen exemplars

LLM inputs were rendered from the same primary predictor inventory into natural-language profiles. Before the formal run, all prompt templates were fixed. During the core run, prompts were not revised based on Test or Holdout results. The system prompt explicitly required the model to use only the participant variables provided in the prompt. The model was instructed not to infer unprovided information, use external knowledge, or interpret the task as a clinical diagnostic task. All LLMs were required to return structured JSON with three fields: label, prob_positive, and evidence.

This study designed four prompt families. A was the direct baseline. It provided only the basic screening prediction task, the JSON output format, and rules prohibiting diagnostic wording. B was the rule-informed baseline. Building on A, it further specified the SDS≥53 threshold, the non-SDS input variables, and the nondiagnostic boundary. It also required prob_positive to be consistent with the final label. E1 added one negative medoid exemplar and one positive medoid exemplar under the same rule boundary as B. E2 added two frozen exemplars from each class under the same rule boundary as B, including a medoid exemplar and a diverse exemplar. A and B were both zero-shot conditions. E1 corresponded to a balanced two-shot condition, and E2 corresponded to a balanced four-shot condition.

Frozen exemplars were selected only from Development-training. Samples from Test and Holdout were not used for exemplar selection. Before exemplar selection, continuous variables were z-score standardized within Development-training. The sex variable was represented using one-hot encoding. These steps formed a fixed exemplar selection distance space. Within each class, the class center was first calculated. The sample closest to the class center was then selected as the medoid exemplar. Next, within the IQR range of the within-class distance distribution, a sample farther from the medoid was selected as the diverse exemplar. This rule avoided selecting both extreme outliers and samples that were overly similar to the medoid.

The four prompt conditions represented progressively richer bundles of task instructions and examples. A and B differed in task boundary, threshold specification, and output-consistency constraints. E1 and E2 also added balanced frozen demonstrations, displayed output formats, and probability anchors. Comparisons are therefore described as differences between prompt conditions. They do not isolate a causal effect of exemplar number, and we make no claim of a pure few-shot effect.

### 2.5 LLM conditions and calling settings

Three commercially hosted LLM families were accessed through a single OpenRouter interface using fixed slugs: openai/gpt-5.4-mini, anthropic/claude-haiku-4.5, and google/gemini-3-flash-preview. The design pragmatically selected one accessible, low-latency model from each of three provider families. This selection allowed prompt-condition behavior to be examined under a common access layer.

Core LLM conditions used temperature=0.1, top_p=1.0, max_tokens=200, disabled reasoning, and a JSON-object response constraint. GPT calls set reasoning effort to none. Limited post-processing parsed JSON, normalized prob_positive, and recorded whether a usable structured output remained. Operational structured-output validity required a successful API response and a valid normalized probability after this limited procedure. It did not denote raw JSON-syntax validity before repair.

The primary fixed-threshold evaluation regenerated label_eval from normalized prob_positive≥0.5 rather than using the model’s self-reported label. Supplementary hard-label analyses regenerated decisions at the locked Development-derived thresholds. Percentage strings were converted to proportions; numeric values above 1 and up to 100 were divided by 100. Values outside [0, 1] after normalization were invalid. Invalid outputs received an evaluation-only error penalty in failure-penalized hard-label metrics and were excluded from probability metrics. Every probability table reports its valid denominator.


$${\hat{y}}_{i}=I\left(p_{i}\geq 0.5\right)$$

where *p_i_* denotes the parsed and normalized prob_positive for sample *i*, and I(·) denotes the indicator function. ${\hat{y}}_{i}=1\;$ indicates that the model predicted student *i* as SDS screen-positive at the fixed 0.5 threshold. ${\hat{y}}_{i}=0$ indicates a screen-negative prediction.

### 2.6 Traditional machine learning baselines

LR and XGBoost were used as traditional structured modeling references and shared the same primary predictor inventory as the LLMs. Their hyperparameters were selected within Development-training through cross-validation, and final models were fitted on Development-training. XGBoost was further evaluated on Development-validation to compare sigmoid and isotonic calibration. ML results were reported on Test and Holdout as references for hard-label screening performance, probability ranking, and probability quality.

The preprocessing order was fixed in each machine learning workflow. LR used the sex variable after imputation and one-hot encoding. It used continuous predictors after median imputation and standardization. It also used L2 regularization and balanced class weights. XGBoost used the same categorical-variable encoding and continuous-variable imputation procedures, but it did not standardize continuous predictors. Both traditional ML baselines used 5-fold stratified cross-validation within Development-training. Hyperparameters were selected first by macro-F1 and then by log loss if further separation was needed. Complete model instantiation parameters are reported in **Supplementary Table S4**.

Logistic Regression used L2 regularization and class_weight=balanced. Sex was mode-imputed and one-hot encoded, and continuous variables were median-imputed and standardized, although no missing predictors were present in the primary analysis. C was selected from 0.01, 0.1, 1, 10, and 100 using 5-fold stratified cross-validation within Development-training. Selection prioritized macro-F1 and then log loss. The selected LR model was refitted on full Development-training and fixed for Test and Holdout prediction.

XGBoost hyperparameters and isotonic calibration were selected entirely within Development, as described above. The main Test and Holdout tables retain the fixed 0.5 hard-label threshold. In the supplementary analysis, each frozen model or prompt condition received a separate threshold selected only in Development-validation. These thresholds were then applied unchanged to Test and Holdout. No threshold was optimized in an evaluation cohort.

### 2.7 Evaluation metrics

Evaluation covered hard-label screening performance, probability discrimination and quality, operational structured-output validity, repeated-output stability, threshold robustness, construct overlap, and cohort shift. It also included exploratory subgroup and decision-consequence analyses. Hard-label metrics were accuracy, sensitivity, specificity, precision, and macro-F1. Probability metrics were AUROC, AUPRC, Brier score, calibration intercept and slope, and ECE under multiple binning specifications.

Hard-label metrics used the full cohort, with invalid LLM outputs counted as errors. Macro-F1 remained the primary summary because the screen-positive class was smaller, but it was interpreted only at the stated threshold. Sensitivity, specificity, and confusion counts were reported alongside macro-F1.


$$Macro-F1=\frac{F1_{positive}+F1_{negative}}{2}$$

AUROC, AUPRC, Brier score, and calibration measures used valid probabilities. ML models used the complete evaluation cohorts, whereas LLMs used operationally valid cases. The primary descriptive ECE used 10 equal-width bins. Enhanced analyses reported logistic calibration intercepts and slopes and Brier confidence intervals. They calculated ECE using 5, 10, 15, and 20 equal-width or equal-frequency bins. Results were reported for both model-native valid denominators and the four-model common-valid intersection. Predicted probabilities were clipped to [10^−6^, 1-10^−6^] only for calibration-logit regression. An intercept of 0 and a slope of 1 denote ideal calibration.


$$Brier=\frac{1}{n_{prob}}\sum_{i=1}^{n_{prob}}{\left(p_{i}-y_{i}\right)}^{2}$$

$$ECE=\sum_{b=1}^{B}\frac{n_{b}}{n_{prob}}\left|obs\left(b\right)-conf\left(b\right)\right|$$

where *p_i_* denotes the predicted positive probability for sample *i*, *y_i_* denotes the true label, *B* denotes the number of bins, and *n_b_* denotes the number of samples in bin *b*. *obs*(*b*) denotes the observed positive rate in that bin. *conf*(*b*) denotes the mean predicted probability in that bin. For LLM conditions, both *n_prob_* and *n_b_* were computed based on the operationally structured-output-valid subset. Invalid outputs were not included in probability metrics. They were counted as incorrect predictions under the conservative penalty rule in hard-label metrics.

Operational structured-output validity was measured by the operational structured-output validity rate and invalid output rate. The former indicated that the API returned successfully, the response could be parsed or repaired into a JSON object, and a valid normalized prob_positive could be obtained. It was not equivalent to raw JSON syntax parsing success.

The primary stability analysis repeatedly called each model with identical inputs, prompts, slugs, and temperature=0.1. For participant i and repeat r, ŷ_ir denoted the binary evaluation label and v_ir indicated an invalid output; inconsistency meant differing ŷ_ir values or any v_ir=1. The Test subset contained 120 class-balanced cases and was retained as an explicitly non-representative condition-comparison experiment. The two finalists also underwent 10 repeated calls across the complete Holdout cohort.


$$I_{i}=I\left(\underset{1\leq r\leq R}{\min}{\hat{y}}_{ir}\neq \underset{1\leq r\leq R}{\max}{\hat{y}}_{ir}\vee \underset{1\leq r\leq R}{\max}v_{ir}=1\right)$$

Votes used the binary evaluation label over R repeats (R = 10 for Test and complete Holdout; R = 5 for the representative 400-case analysis). The vote margin for sample *i* was defined as the absolute difference between the number of positive and negative recorded evaluation labels, divided by the number of repeated runs:


$$n_{i1}=\sum_{r=1}^{R}{\hat{y}}_{ir},\;n_{i0}=R-n_{i1}$$

$$M_{i\;}=\frac{\left|n_{i1}-\;n_{i0}\right|}{R}$$

where *n_i_*_1_ and *n_i_*_0_ denote the numbers of times that the sample was recorded as a positive and negative evaluation label across repeated runs. The mean vote margin was the average of *M_i_* across all samples:


$$\bar{M}=\frac{1}{N}\sum_{i=1}^{N}M_{i}$$

Its range was 0 to 1. Values closer to 1 indicated more concentrated voting across repeated runs. Values closer to 0 indicated that positive and negative votes were closer. This metric measured the strength of repeated-output agreement. It did not indicate whether the modal label was positive or negative. The modal evaluation label was defined as positive when $n_{i1}\geq n_{i0}$. Otherwise, it was defined as negative. Mode macro-F1 was computed from these modal evaluation labels. Prediction entropy used a base-2 logarithm:


$$H_{i}=-\sum_{c\in \left\{0,1\right\}}q_{ic}{\mathrm{lo}ɡ}_{2}\left(q_{ic}\right)$$

where $q_{ic}={\text{n}}_{ic}\text{/R}$. When $q_{ic}=0$, the standard entropy convention was used, and $q_{ic}\log_{2}\left(q_{ic}\right)$ was set to 0.

Any parsing or API failure caused the participant to be counted as inconsistent; failure counts were also reported separately. Vote margin, modal evaluation label, mode macro-F1, and entropy were computed from the binary evaluation label for each repeat. Runs with parsing or API failure were assigned the prespecified evaluation penalty label. These metrics therefore followed the failure-penalized evaluation rule rather than relying on the model’s raw label.

### 2.8 Test comparison, within-institution validation, and stability design

All 12 LLM conditions were run on Test. They were formed by combining three model families and four prompt families. Test also evaluated LR and XGBoost as traditional structured baselines. For each LLM condition, the study reported hard-label screening performance, probability ranking, probability quality, and operational structured-output validity. Repeated-output stability was reported in an independent stability analysis. Test results were used to describe prompt family patterns and model family patterns across LLM conditions. They were also used to select two LLM finalists for Holdout.

The primary Test stability subset comprised 60 screen-positive and 60 screen-negative cases, each called 10 times at temperature=0.1. Complete-Holdout stability used 10 repeats for each finalist. Using seed 42, the supplementary stability experiment sampled 400 Holdout students by proportional stratification on observed SDS screen-positive status and sex, thereby retaining the observed prevalence. Each finalist received five calls at temperature=0 and five calls at temperature=0.1, with all other settings fixed. No generation seed was set. The analysis therefore assessed the repeated behavior of the hosted interface rather than seeded local determinism.

The prespecified finalist rule ranked the 12 Test LLM conditions lexicographically by descending macro-F1, ascending Brier score, ascending invalid-output rate, and descending AUROC. GPT E1 and Gemini E2 therefore entered Holdout before the supplementary analyses. They were not reselected after the threshold or bootstrap analyses.

Holdout evaluated the two Test-selected LLM finalists and both structured-data comparators. It assessed transport across grade cohorts within the same institution and survey period.

### 2.9 Statistical reporting principles

All bootstrap analyses used 1,000 subject-level resamples with seed 42. Wilson intervals were used for cohort proportions, supplementary-analysis subgroup output-validity rates, and inconsistent-case rates; calibration intercepts and slopes used Wald intervals from binomial logistic models. Primary-analysis single-model bootstrap intervals, including those for valid- and invalid-output rates, were retained. Supplementary comparisons used paired resampling, so each model pair received the same subject indices in every replicate. Hard-label differences used the full cohort. AUROC and Brier differences used the intersection of cases with valid probabilities for both models. We reported percentile 95% confidence intervals for differences without multiplicity-adjusted significance claims. Formal normality tests were not performed because, at N = 6,395, they would be highly sensitive to minor departures from normality, and the bounded psychometric scale scores were not assumed to follow Gaussian distributions. Scale associations were therefore evaluated using Spearman correlations. Cohort distributions were described using means and standard deviations together with medians and interquartile ranges, with standardized mean differences used for location shifts and two-sample Kolmogorov–Smirnov statistics used for distributional differences.

Interpretation focused on effect direction, magnitude, uncertainty, and metric trade-offs. Output tables recorded cohort n, valid-probability n, threshold source, seed, and data/configuration hashes.

### 2.10 Development-only threshold selection and operating points

All 12 LLM conditions were run on Development-validation at temperature=0.1. LR and the frozen isotonic XGBoost model generated probabilities for the same 391 students. For each condition, the Youden threshold maximized sensitivity+specificity-1. Ties were resolved first by greater sensitivity and then by proximity to 0.5. Probabilities and thresholds were compared after canonical rounding to 12 decimals. This procedure handled discrete isotonic-score ties consistently across CSV files. Each threshold was locked and applied unchanged to Test and Holdout, whereas the prespecified 0.5 results were retained in parallel.

Threshold sensitivity was evaluated from 0.05 to 0.95 in increments of 0.01 using sensitivity, specificity, and macro-F1. Development-only operating points also identified the highest specificity available at sensitivity ≥0.80 and the highest sensitivity available at specificity ≥0.80. Discrete probabilities may not attain exactly 0.80. We therefore report the achieved evaluation-cohort values rather than assuming exact equality.

### 2.11 Paired model differences and ranking stability

Paired bootstrap differences were calculated for macro-F1, sensitivity, specificity, AUROC, and Brier score. Test analyses compared the three highest-ranked primary-analysis LLM conditions pairwise, each finalist with LR and XGBoost, and the two finalists with each other. Holdout analyses repeated the finalist-versus-baseline and finalist-versus-finalist comparisons. Hard-label differences were evaluated at 0.5 and at Development-derived thresholds. Within each bootstrap replicate, we also reranked all 12 Test LLM conditions. This analysis estimated rank-1 frequency, top-2 frequency, and median rank and interquartile range.

### 2.12 Construct overlap and grouped ablation

Spearman correlations were calculated among continuous SDS, SAS, CFQ, AAQ-II, the eight eligible non-depression SCL-90 symptom dimensions, and the additional-items score in Development, Test, Holdout, and the combined sample. SDS correlations received bootstrap 95% confidence intervals. LR and XGBoost were then refitted without new hyperparameter searches. Specifications comprised the full model, four leave-one-questionnaire-group-out models, and four single-questionnaire-group-plus-demographics models. The prespecified XGBoost isotonic calibration method was retained. Changes in Test and Holdout AUROC, Brier score, and macro-F1 received paired bootstrap intervals. These analyses quantified predictive dependence and sufficiency under correlated inputs, not causal importance. As a further benchmark, LR and XGBoost models restricted to sex and age were fitted without an additional hyperparameter search and evaluated on Test and Holdout. Their AUROC and Brier scores were compared with those of the corresponding full models using 1,000 paired subject-level bootstrap resamples.

For exploratory LLM input sensitivity, GPT E1 and Gemini E2 were evaluated on the same representative 400-case Holdout subset at temperature=0. The analysis separately removed SAS, CFQ, AAQ-II, or all eligible SCL-90 fields. Each removal was applied to both target profiles and every frozen demonstration. The first temperature=0 full-input repeat served as the reference. Changes in macro-F1, AUROC, and Brier score received paired bootstrap intervals.

### 2.13 Cohort distribution shift

Continuous predictors were summarized by mean, standard deviation, median, and interquartile range. For each cohort pair, standardized mean differences (SMDs) quantified location shifts. Two-sample Kolmogorov–Smirnov statistics described changes in distribution shape. The pairs were Development-Test, Development-Holdout, and Test-Holdout. Sex was summarized by proportions and binary SMDs. SDS screen-positive prevalence was summarized with Wilson intervals and absolute differences. We did not emphasize variable-level P values because large samples can make trivial differences appear significant.

### 2.14 Enhanced calibration analysis

LR, XGBoost, GPT E1, and Gemini E2 were evaluated on Test and Holdout. For GPT E1 and Gemini E2, calibration analyses used operationally valid raw model-reported probabilities after normalization, without fitting a *post-hoc* calibration model. After logit clipping, a binomial logistic calibration model estimated the intercept and slope with 95% confidence intervals. Brier scores received bootstrap intervals. ECE was recalculated using 5, 10, 15, and 20 equal-width or equal-frequency bins. Results were reported for each model’s native valid sample and for the four-model common-valid intersection. Calibration plots display bin counts, and histograms stratify predicted probabilities by the true label.

### 2.15 Repeated-output stability analyses

The 400-case experiment reported the inconsistent-case rate with a Wilson interval, prediction entropy, mean vote margin, modal-label macro-F1, and each participant’s probability range. Temperature differences in inconsistency received paired bootstrap intervals. For the complete-Holdout repeats, we grouped each participant by the distance of their median probability from 0.5:<0.05, 0.05-<0.10, 0.10-<0.20, or ≥0.20. Inconsistency was also summarized by outcome and sex. Two exploratory mitigation analyses were then conducted using the complete-Holdout repeated calls. First, probability-margin review deferred cases when the absolute distance between the median predicted probability and 0.5 was below 0.05, 0.10, or 0.20; we reported deferred coverage, the proportion of inconsistent cases captured, and the inconsistent-case rate among retained cases. Second, majority-vote labels across the 10 calls were compared with the mean single-call macro-F1 using participant-level bootstrap resampling. A backend-drift audit compared the April and August 2026 temperature=0.1 call windows using label agreement, probability mean absolute error, and Spearman correlation. This audit was not interpreted as strict version reproduction.

### 2.16 Exploratory subgroup and screening-consequence analyses

LR, XGBoost, GPT E1, and Gemini E2 were evaluated separately for female and male students in Test and Holdout. Each subgroup report included n, positive cases, sensitivity, specificity, Brier score, calibration intercept and slope, LLM valid-output rate, and bootstrap intervals. Female-minus-male differences received subject-level bootstrap intervals. Age was not dichotomized because the undergraduate range was narrow and any cutoff would be arbitrary. These analyses were exploratory and did not constitute a comprehensive fairness validation.

To describe screening consequences without claiming validated clinical utility, observed Test and Holdout prevalence was standardized to 1,000 students. At 0.5 and at Development-derived thresholds, we reported true positives, false positives, missed positives, true negatives, and the number requiring further review. Illustrative false-negative:false-positive cost ratios of 1:1, 2:1, 5:1, and 10:1 yielded standardized losses. These scenarios were transparent illustrations, not empirically validated clinical costs or a decision-curve analysis.

## 3 Results

### 3.1 Sample description and predictor inventory

The fixed cohorts included 1,951 Development, 2,186 Test, and 2,258 Holdout students. SDS≥53 rates were 16.3%, 24.4%, and 27.9%, respectively. **Table 1** reports sex, age, outcome prevalence, and the internal Development split. These grade-specific cohorts differed materially in age and outcome prevalence.

Stratification by the SDS≥53 label produced a Development-training subset of 1,560 records, including 254 screen-positive cases (16.3%). The Development-validation subset contained 391 records, including 64 screen-positive cases (16.4%).

The primary predictor inventory was identical for the ML and LLM conditions. No cleaned split contained missing primary predictors. SDS, SCL-90 depression, and aggregated SCL-90 summaries were excluded from all primary models. ML models used continuous age, whereas LLM profiles displayed rounded integer age for readability.

### 3.2 Cohort distribution shift and construct overlap

Age showed the largest cohort shift. Absolute SMDs were 0.96 for Development-Test, 1.93 for Development-Holdout, and 1.11 for Test-Holdout. The differences in SDS screen-positive prevalence were 8.1 percentage points for Development-Test, 11.6 for Development-Holdout, and 3.5 for Test-Holdout. For psychological predictors, Development-Holdout SMDs were 0.25 for SAS and no greater than 0.19 for the other reported variables. Test-Holdout shifts other than age were small. **Supplementary Figure S3** and **Supplementary Table S9** provide the complete SMD, Kolmogorov–Smirnov, and distribution summaries.

SDS correlated positively with every questionnaire predictor. In the combined sample, Spearman rho was 0.60 (95% CI 0.58-0.61) for SAS, 0.52 for SCL-90 anxiety, and 0.51 for somatization. Corresponding values were 0.47 for AAQ-II and 0.43 for CFQ. SAS was also the strongest SDS correlate in Development, Test, and Holdout, with rho values of 0.56, 0.59, and 0.62, respectively. These correlations documented substantive construct overlap despite the exclusion of SDS and SCL-90 depression.

For both LR and XGBoost, omitting SAS reduced AUROC by approximately 0.025-0.029 and worsened the Brier score by 0.008-0.013 across Test and Holdout. Omitting the SCL-90 group reduced XGBoost AUROC by 0.019 on Test and 0.018 on Holdout. The Brier score increased by approximately 0.009-0.010. The effects of CFQ and AAQ-II were smaller and depended on the metric. Some leave-one-out macro-F1 values increased because each refitted model received a separate Development threshold. In the 400-case LLM analysis, removing SAS increased the Brier score by 0.035 for GPT E1 and 0.017 for Gemini E2, whereas macro-F1 intervals included zero. No single questionnaire group consistently matched the full-input model across models, cohorts, and metrics. Models restricted to demographics provided little discrimination. Demographics-only LR yielded AUROCs of 0.531 on Test and 0.538 on Holdout, compared with 0.844 and 0.832 for the full LR model; demographics-only XGBoost yielded AUROCs of 0.498 and 0.521, compared with 0.846 and 0.835 for the full model. All paired confidence intervals for the demographics-only minus full-model AUROC differences excluded zero. These comparisons indicated that sex and age alone did not account for the benchmark performance. **Supplementary Figures S1**, **S2** and **Supplementary Table S8** report the correlation matrix, grouped ablations, LLM input-ablation results, and demographics-only comparisons; the latter are presented in **Supplementary Table S8E**.

### 3.3 Performance of traditional ML baselines

At the prespecified 0.5 threshold, LR produced a more balanced sensitivity-specificity profile, whereas isotonic XGBoost assigned positive labels conservatively. XGBoost nevertheless yielded favorable AUROC, Brier score, and descriptive ECE. **Table 3** reports the prespecified 0.5 results. **Supplementary Table S6** reports Development-derived thresholds and fixed operating points separately.

**Table 3:** Traditional ML results on test and holdout at the prespecified fixed 0.5 threshold.

Condition	Split	Macro-F1 (95% CI)	Sens.	Spec.	Prec.	Acc.	AUROC (95% CI)	AUPRC	Brier	ECE
LR	Test	0.710 (0.689-0.728)	0.785	0.737	0.491	0.748	0.844 (0.824-0.862)	0.701	0.174	0.204
LR	Holdout	0.712 (0.692-0.731)	0.784	0.720	0.521	0.738	0.832 (0.813-0.851)	0.710	0.179	0.187
XGBoost	Test	0.674 (0.649-0.701)	0.316	0.979	0.833	0.817	0.846 (0.829-0.865)	0.639	0.129	0.045
XGBoost	Holdout	0.632 (0.609-0.655)	0.266	0.976	0.812	0.778	0.835 (0.818-0.853)	0.636	0.147	0.062

Sens., sensitivity; Spec., specificity; Prec., precision; Acc., accuracy; ECE, primary 10-bin equal-width expected calibration error. **Supplementary Table S6** reports development-derived thresholds and fixed-sensitivity and fixed-specificity operating points.

The final regularization parameter used by LR was C = 0.1. This parameter was selected through cross-validation within Development-training. The final XGBoost parameters were n_estimators=500, max_depth=2, learning_rate=0.05, subsample=0.8, colsample_bytree=1.0, min_child_weight=3, and reg_lambda=1.0. These hyperparameters were also selected through cross-validation within Development-training. XGBoost was then evaluated on Development-validation to compare and fit sigmoid and isotonic calibration. Isotonic calibration was selected. Its Development-validation Brier score was 0.106865, which was lower than the value of 0.116083 for sigmoid calibration.

At 0.5 on Test, LR achieved sensitivity 0.785, specificity 0.737, macro-F1 0.710, AUROC 0.844, AUPRC 0.701, and Brier score 0.174. The corresponding Holdout values were 0.784, 0.720, 0.712, 0.832, 0.710, and 0.179.

At 0.5 on Test, XGBoost achieved sensitivity 0.316, specificity 0.979, macro-F1 0.674, AUROC 0.846, AUPRC 0.639, and Brier score 0.129. On Holdout, sensitivity was 0.266, specificity 0.976, macro-F1 0.632, AUROC 0.835, AUPRC 0.636, and Brier score 0.147. The fixed 0.5 decision rule therefore missed many screen-positive cases, although ranking and squared probability error remained competitive.

### 3.4 Main comparison of 12 LLM conditions on Test

A total of 12 LLM model-prompt conditions were evaluated on Test at the prespecified 0.5 threshold. These results retain the prespecified comparison used for finalist selection. They report hard-label performance, probability discrimination and quality, and operational structured-output validity. **Supplementary Table S2** provides complete point estimates and single-model bootstrap intervals.

GPT E1, Gemini E2, and GPT E2 had the three highest Test macro-F1 point estimates: 0.732, 0.730, and 0.729. Their corresponding AUROCs were 0.818, 0.834, and 0.826, and their Brier scores were 0.157, 0.167, and 0.153, respectively.

Mean macro-F1 across A, B, E1, and E2 was approximately 0.622, 0.639, 0.708, and 0.724, respectively. The example-augmented conditions therefore showed a more favorable fixed-0.5 pattern in this benchmark. E1 and E2 also changed output-format demonstrations, class balance, and probability anchors. **Figure 2** shows the fixed-0.5 macro-F1 and AUROC trade-off.

**Figure 2:**
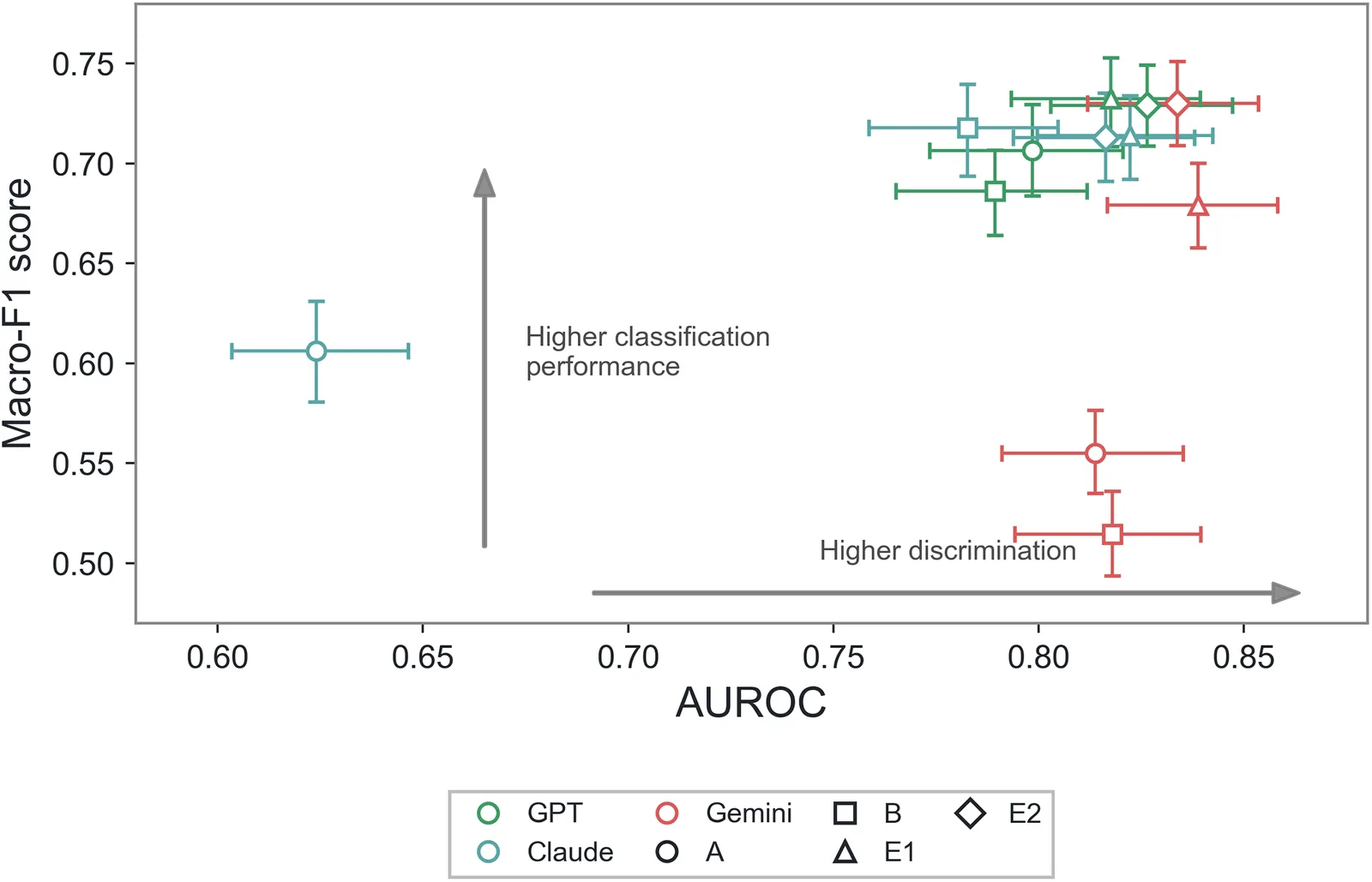
Macro-F1 and AUROC across 12 LLM conditions on test at the fixed 0.5 threshold. Each point represents one model-prompt condition; error bars show single-model bootstrap 95% confidence intervals. The leading macro-F1 point estimates were closely matched. **Supplementary Table S7** therefore reports paired differences and rank frequencies.

Model-family patterns varied by metric and prompt condition. GPT conditions had relatively high macro-F1 across the four prompts. Gemini E2 combined high macro-F1 with strong AUROC, whereas some Gemini conditions had less favorable Brier score, ECE, or structured-output validity. Claude B, E1, and E2 achieved similar fixed-0.5 macro-F1 despite differences in stability. **Figure 3** presents probability quality and output validity jointly.

**Figure 3:**
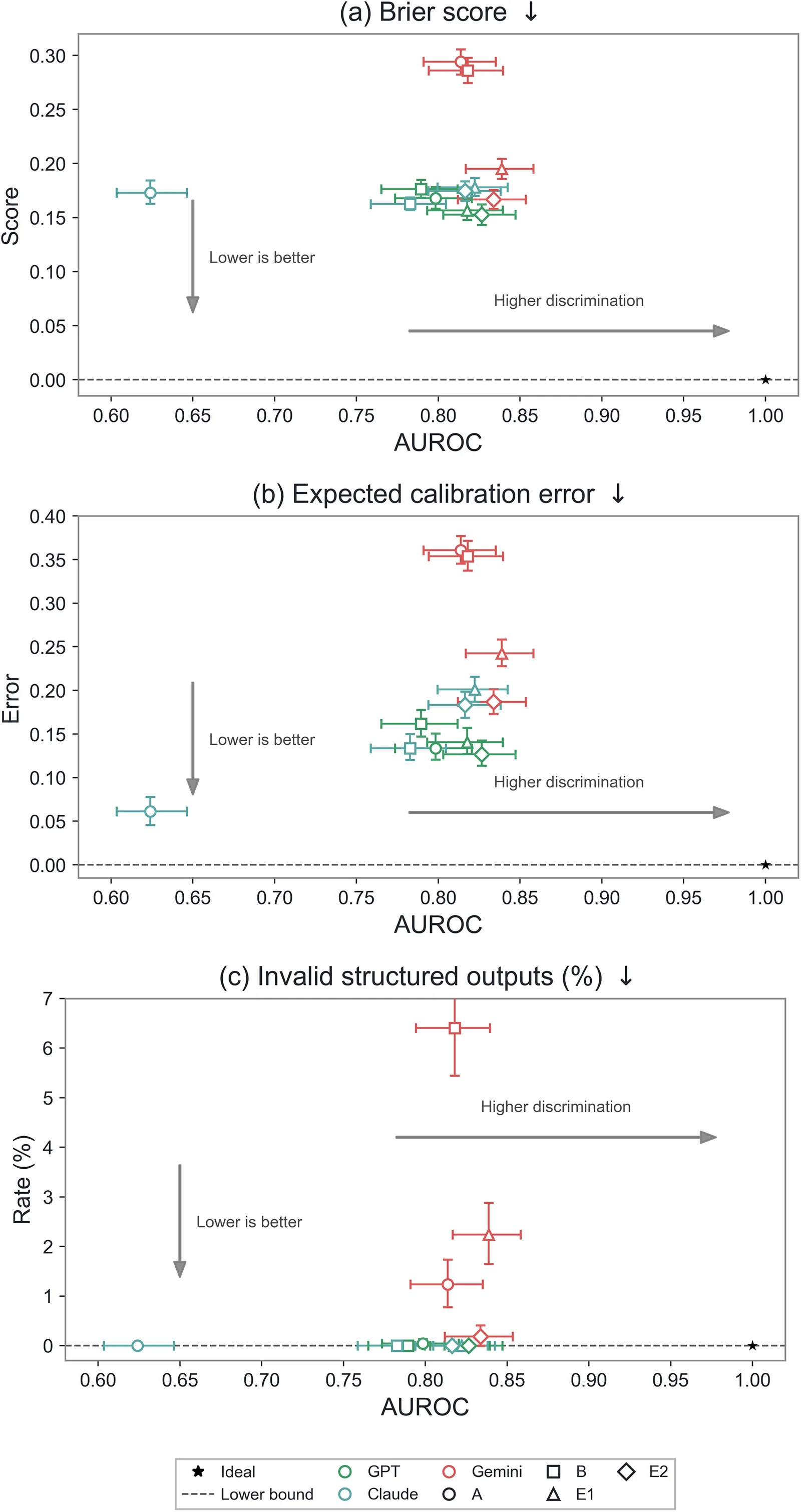
Probability quality and operational structured-output validity across 12 LLM conditions on test. Panels **(a–c)** plot Brier score, the primary 10-bin equal-width ECE, and invalid-output rate against AUROC, respectively. Probability metrics include operationally valid outputs only; failure-penalized hard-label scoring treats invalid outputs as errors. **Supplementary Figures S4**–**S6** and **Supplementary Table S10** report the enhanced calibration analyses.

### 3.5 Independent within-institution Holdout validation

Holdout was limited to GPT E1, Gemini E2, LR, and XGBoost and did not repeat the 12-condition search. It therefore assessed transport from the Test grade cohort to another grade cohort in the same university and survey period. **Table 4** reports the two LLM finalist results.

**Table 4:** Prespecified fixed-0.5 results for the test-selected LLM finalists in the independent within-institution Holdout cohort.

Condition	Split	Macro-F1	Sens.	Spec.	Prec.	Acc.	AUROC	AUPRC	Brier	ECE	Invalid, n/N (%)
GPT E1	Holdout	0.729 (0.711-0.749)	0.655	0.821	0.586	0.774	0.808 (0.788-0.830)	0.668	0.160	0.113	0/2258 (0.00%)
Gemini E2	Holdout	0.739 (0.720-0.758)	0.723	0.796	0.579	0.775	0.820 (0.801-0.839)	0.664	0.167	0.151	2/2258 (0.09%)

Probability metrics include operationally valid cases only. **Supplementary Table S6** reports alternative thresholds.

At 0.5 on Holdout, GPT E1 achieved macro-F1 0.729, sensitivity 0.655, specificity 0.821, AUROC 0.808, and Brier score 0.160, with no invalid outputs. It identified 413 of 631 screen-positive cases. **Figure 4** shows the fixed-0.5 confusion matrices for all four models.

**Figure 4:**
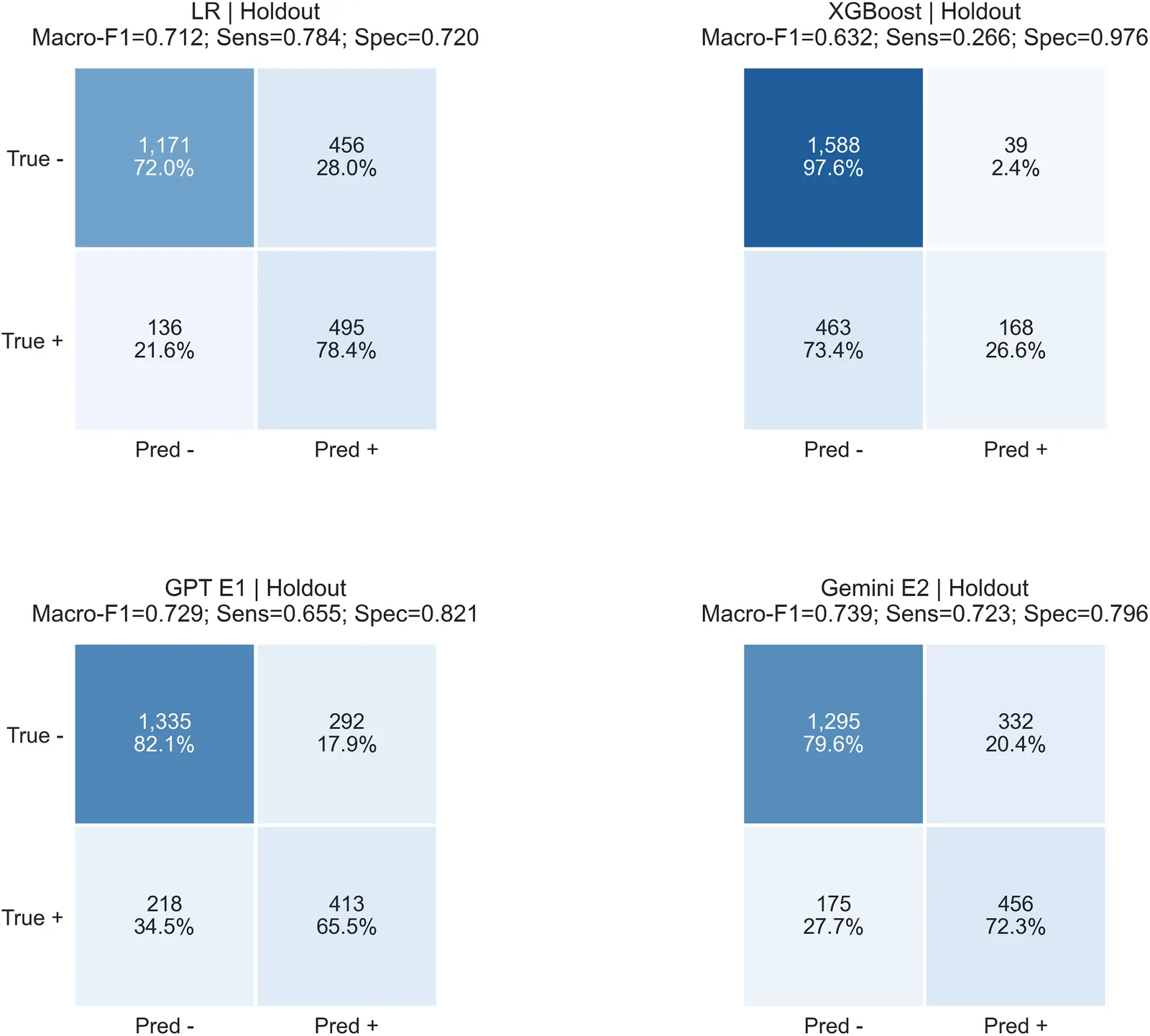
Holdout confusion matrices for LR, XGBoost, GPT E1, and Gemini E2 at the fixed 0.5 threshold. Cells show counts and row percentages within the true class. This image presents the prespecified common-threshold comparison. **Supplementary Table S6** and **Supplementary Figure S7** report Development-derived thresholds and fixed operating points.

At 0.5 on Holdout, Gemini E2 achieved macro-F1 0.739, sensitivity 0.723, and specificity 0.796. AUROC was 0.820 and Brier score 0.167, with two invalid outputs among 2,258 cases. It identified 456 of 631 screen-positive cases. Gemini E2 had higher sensitivity, whereas GPT E1 had higher specificity and a lower Brier score.

The fixed-0.5 Test order did not translate into a clear pairwise advantage on Holdout. **Figure 5** compares Test and Holdout macro-F1 at 0.5 with threshold-independent AUROC and Brier scores. Paired intervals and alternative operating points are reported below.

**Figure 5:**
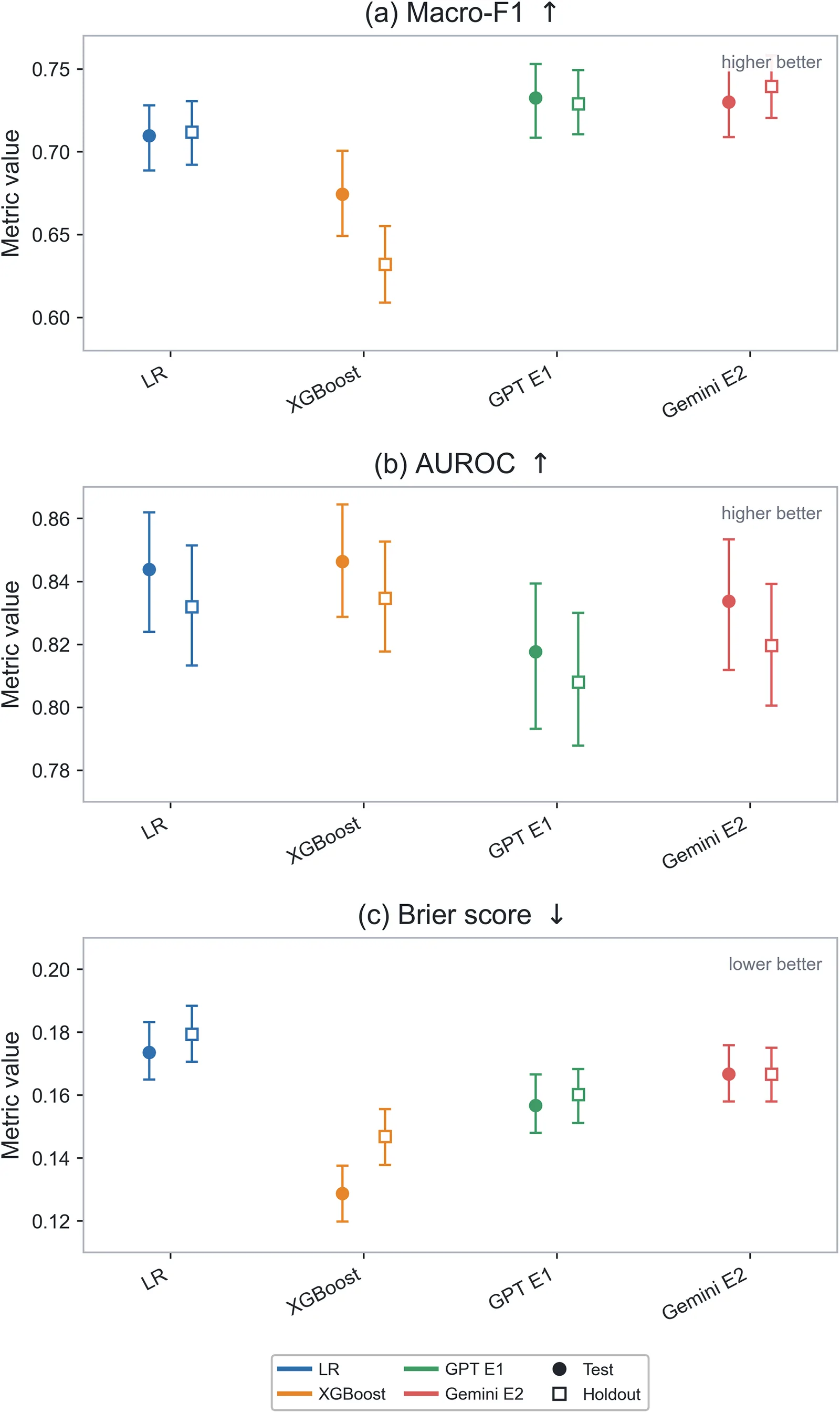
Test and independent within-institution Holdout performance of LR, XGBoost, GPT E1, and Gemini E2. **(a)** shows macro-F1 at the prespecified 0.5 threshold; **(b, c)** show AUROC and Brier score, respectively. Error bars indicate bootstrap 95% confidence intervals.

### 3.6 Threshold sensitivity, fixed operating points, and paired uncertainty

Development-derived thresholds were 0.466 for LR, 0.174 for XGBoost, 0.38 for GPT E1, and 0.50 for Gemini E2. On Test, macro-F1 changed from 0.710 to 0.695 for LR, from 0.674 to 0.660 for XGBoost, and from 0.732 to 0.702 for GPT E1. It remained 0.730 for Gemini E2. The XGBoost threshold shift increased sensitivity from 0.316 to 0.871 and reduced specificity from 0.979 to 0.622. Its conservative result at 0.5 therefore depended on the chosen cutoff. On Holdout, macro-F1 at the Development-derived thresholds was 0.696, 0.652, 0.709, and 0.739, respectively.

At Development-selected operating points targeting specificity ≥0.80, Test macro-F1 was 0.724 for LR, 0.738 for XGBoost, 0.726 for GPT E1, and 0.730 for Gemini E2. Achieved Test specificity was 0.768, 0.791, 0.795, and 0.788. Cohort transport and discrete scores prevented exact equality with the Development target. At operating points targeting sensitivity ≥0.80, Test macro-F1 ranged from 0.610 to 0.695, with a greater review burden. **Supplementary Figure S7** shows the complete threshold curves, and **Supplementary Table S6** reports all thresholds and operating points.

At 0.5, pairwise Test macro-F1 differences among GPT E1, Gemini E2, and GPT E2 were 0.001-0.003, and every 95% interval included zero. The difference between GPT E1 and Gemini E2 was 0.002 (95% CI −0.013 to 0.016). At Development-derived thresholds, GPT E1 minus Gemini E2 was −0.028 (−0.040 to −0.016). GPT E1 minus XGBoost was 0.042 (0.025-0.060), and Gemini E2 minus XGBoost was 0.070 (0.052-0.086). The direction and magnitude therefore depended on both the model pair and the operating-point rule. In bootstrap reranking at 0.5, GPT E1 ranked first in 49.0% of replicates and Gemini E2 in 26.0%. At Development-derived thresholds, Gemini E2 ranked first in 99.9% of replicates. **Supplementary Table S7** reports all paired differences and ranking frequencies.

### 3.7 Probability discrimination and enhanced calibration

Probability analyses used model-native valid denominators and, for enhanced calibration, the four-model common-valid intersection. On Test, native valid n was 2,186 for LR, XGBoost, and GPT E1 and 2,182 for Gemini E2. On Holdout, the corresponding values were 2,258, 2,258, 2,258, and 2,256. Common-valid and native results were nearly identical. The few invalid finalist outputs therefore did not account for the observed calibration differences.

On Test, AUROC was 0.844 for LR, 0.846 for XGBoost, 0.818 for GPT E1, and 0.834 for Gemini E2. The corresponding Holdout values were 0.832, 0.835, 0.808, and 0.820. The AUPRC ordering differed and should be interpreted alongside cohort prevalence. These results did not support a general discrimination advantage for the LLM finalists over the structured-data models.

XGBoost had the lowest Brier score on Test, at 0.129 (95% CI 0.119-0.138), and on Holdout, at 0.147 (0.138-0.156). Test Brier scores were 0.174 for LR, 0.157 for GPT E1, and 0.167 for Gemini E2. Test calibration intercepts were −1.39 for LR, 0.25 for XGBoost, −0.95 for GPT E1, and −1.21 for Gemini E2. The corresponding slopes were 0.96, 0.93, 0.89, and 1.04. Holdout intercepts were −1.20, 0.46, −0.71, and −0.93, with slopes of 0.93, 1.02, 0.86, and 1.00, respectively. Favorable macro-F1 therefore did not imply calibrated probability estimates, and several intercept intervals excluded the ideal value of zero. These calculations used raw model-reported probabilities for GPT E1 and Gemini E2 without applying *post-hoc* calibration.

Across 5, 10, 15, and 20 equal-width or equal-frequency bins, Test ECE was 0.204 for LR. The ranges were 0.041-0.045 for XGBoost, 0.138-0.148 for GPT E1, and 0.181-0.189 for Gemini E2. Holdout values were 0.187, 0.061-0.063, 0.105-0.114, and 0.146-0.152, respectively. The calibration ordering was therefore not an artifact of the initially reported 10 equal-width bins. **Figure 6** retains the primary descriptive calibration display. **Supplementary Figures S4**–**S6** add bin counts, label-stratified probability histograms, and ECE sensitivity. **Supplementary Table S10** reports intercepts, slopes, Brier scores, ECE, confidence intervals, and denominators.

**Figure 6:**
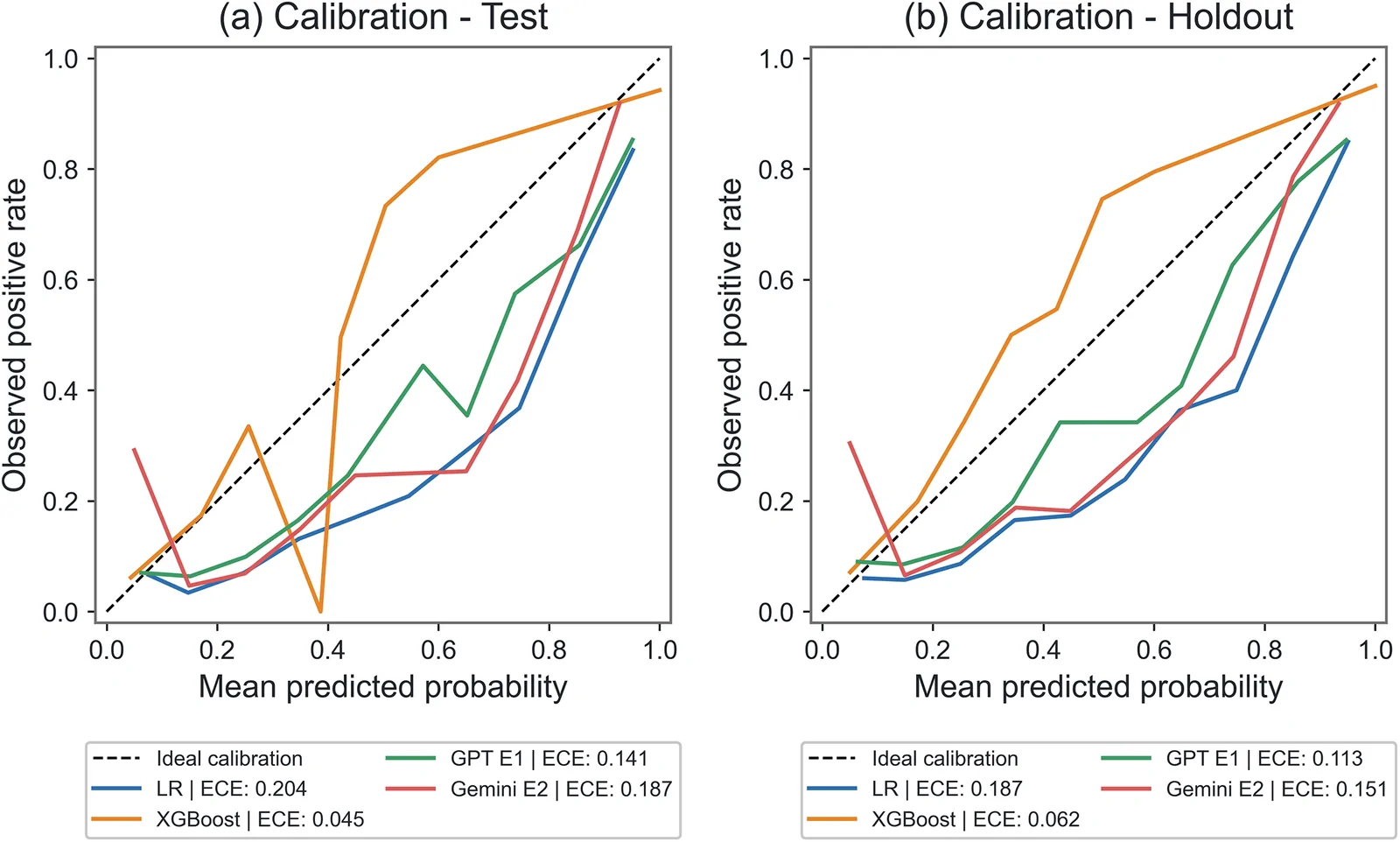
Primary descriptive calibration curves for LR, XGBoost, GPT E1, and Gemini E2 on Test **(a)** and Holdout **(b)**. Observed positive rates are plotted against mean predicted probabilities in 10 equal-width bins. LLM curves include operationally valid raw model-reported probabilities only; no *post-hoc* calibration model was fitted to the LLM outputs. **Supplementary Figures S4**–**S6** and **Supplementary Table S10** report calibration intercepts and slopes, confidence intervals, and common-valid denominators. They also provide label-stratified histograms and ECE sensitivity to binning method and bin count.

### 3.8 Operational structured-output validity

In the primary Test run, GPT and Claude conditions produced zero or nearly zero operationally invalid outputs. GPT A produced 1 invalid output among 2,186 cases. GPT B, E1, and E2 and all four Claude conditions produced none. These values refer to usable outputs after the prespecified limited parsing procedure, not to raw JSON syntax alone.

Invalid outputs were concentrated in the Gemini preview conditions: 27 for A, 140 for B, 49 for E1, and 4 for E2 among 2,186 Test cases. Gemini B combined an AUROC of 0.818 with macro-F1 of 0.514 and operational validity of 0.936. Thus, probability discrimination did not substitute for reliable structured output. **Table 5** and **Figure 3** report these results.

**Table 5:** Results for 12 LLM model-prompt conditions on test at the prespecified fixed 0.5 threshold.

Model	Prompt	Macro-F1 (95% CI)	AUROC	AUPRC	Brier	ECE	Operational structured-output validity rate	Invalid outputs, n/N (%)
GPT	E1	0.732 (0.709-0.753)	0.818	0.652	0.157	0.141	1.0000	0/2186 (0.00%)
Gemini	E2	0.730 (0.709-0.751)	0.834	0.645	0.167	0.187	0.9982	4/2186 (0.18%)
GPT	E2	0.729 (0.709-0.749)	0.826	0.656	0.153	0.127	1.0000	0/2186 (0.00%)
Claude	B	0.718 (0.694-0.740)	0.783	0.523	0.163	0.134	1.0000	0/2186 (0.00%)
Claude	E1	0.714 (0.692-0.734)	0.822	0.621	0.178	0.201	1.0000	0/2186 (0.00%)
Claude	E2	0.713 (0.691-0.735)	0.816	0.612	0.175	0.183	1.0000	0/2186 (0.00%)
GPT	A	0.706 (0.684-0.729)	0.798	0.591	0.168	0.134	0.9995	1/2186 (0.05%)
GPT	B	0.686 (0.664-0.707)	0.789	0.566	0.176	0.162	1.0000	0/2186 (0.00%)
Gemini	E1	0.679 (0.658-0.700)	0.839	0.656	0.195	0.242	0.9776	49/2186 (2.24%)
Claude	A	0.606 (0.581-0.631)	0.624	0.370	0.173	0.061	1.0000	0/2186 (0.00%)
Gemini	A	0.555 (0.535-0.577)	0.814	0.597	0.294	0.361	0.9876	27/2186 (1.24%)
Gemini	B	0.514 (0.494-0.536)	0.818	0.618	0.286	0.354	0.9360	140/2186 (6.40%)

Operational validity requires a successful response and a valid normalized prob_positive after limited parsing. Failure-penalized hard-label metrics treat invalid outputs as errors; probability metrics exclude them. **Supplementary Table S7** reports paired differences and ranking stability.

On Holdout, GPT E1 produced 0 invalid outputs, whereas Gemini E2 produced 2 among 2,258 cases. Across the supplementary-analysis LLM calls, eight invalid structured outputs were observed, all from Gemini conditions. These outputs were retained under the prespecified scoring and denominator rules.

### 3.9 Repeated-output stability and unstable-case localization

**Table 6** summarizes the repeated-output stability analyses. **Supplementary Table S11** provides complete metrics, distance bands, outcome and sex subgroups, and backend drift.

**Table 6:** Principal repeated-output stability results for the two Test-selected LLM finalists.

Condition	Analysis set and temperature	Cases, n	Inconsistent cases, n/N (%; 95% CI)	Mean vote margin	Mode macro-F1	Prediction entropy
GPT E1	Complete Holdout; T = 0.1	2258	532/2258 (23.6; 21.9-25.4)	0.885	0.734	0.172
GPT E1	Representative Holdout subset; T = 0.0	400	62/400 (15.5; 12.3-19.4)	0.913	0.736	0.127
GPT E1	Representative Holdout subset; T = 0.1	400	66/400 (16.5; 13.2-20.5)	0.905	0.744	0.137
Gemini E2	Complete Holdout; T = 0.1	2258	34/2258 (1.5; 1.1-2.1)	0.995	0.739	0.009
Gemini E2	Representative Holdout subset; T = 0.0	400	4/400 (1.0; 0.4-2.5)	0.996	0.730	0.007
Gemini E2	Representative Holdout subset; T = 0.1	400	3/400 (0.8; 0.3-2.2)	0.996	0.730	0.006

The complete-Holdout analysis used 10 repeats at temperature=0.1. The prevalence-representative 400-case subset used five repeats at each temperature. Wilson 95% confidence intervals are shown for inconsistent-case rates. **Supplementary Table S11** reports paired temperature differences, probability ranges, threshold-distance bands, outcome and sex subgroups, exploratory probability-margin review, repeated-call majority-vote aggregation, and backend drift.

In the primary Test subset, Claude E1 and E2 produced no inconsistent cases, Gemini E2 produced 3 of 120, and GPT E1 produced 38 of 120. In complete Holdout at temperature=0.1, GPT E1 had 532 inconsistent cases (23.6%; Wilson 95% CI 21.9%-25.4%). Its mean vote margin was 0.885, entropy was 0.172, and modal macro-F1 was 0.734. Gemini E2 had 34 inconsistent cases (1.5%; 1.1%-2.1%), a mean vote margin of 0.995, entropy of 0.009, and modal macro-F1 of 0.739. See **Figure 7** for relevant results.

**Figure 7:**
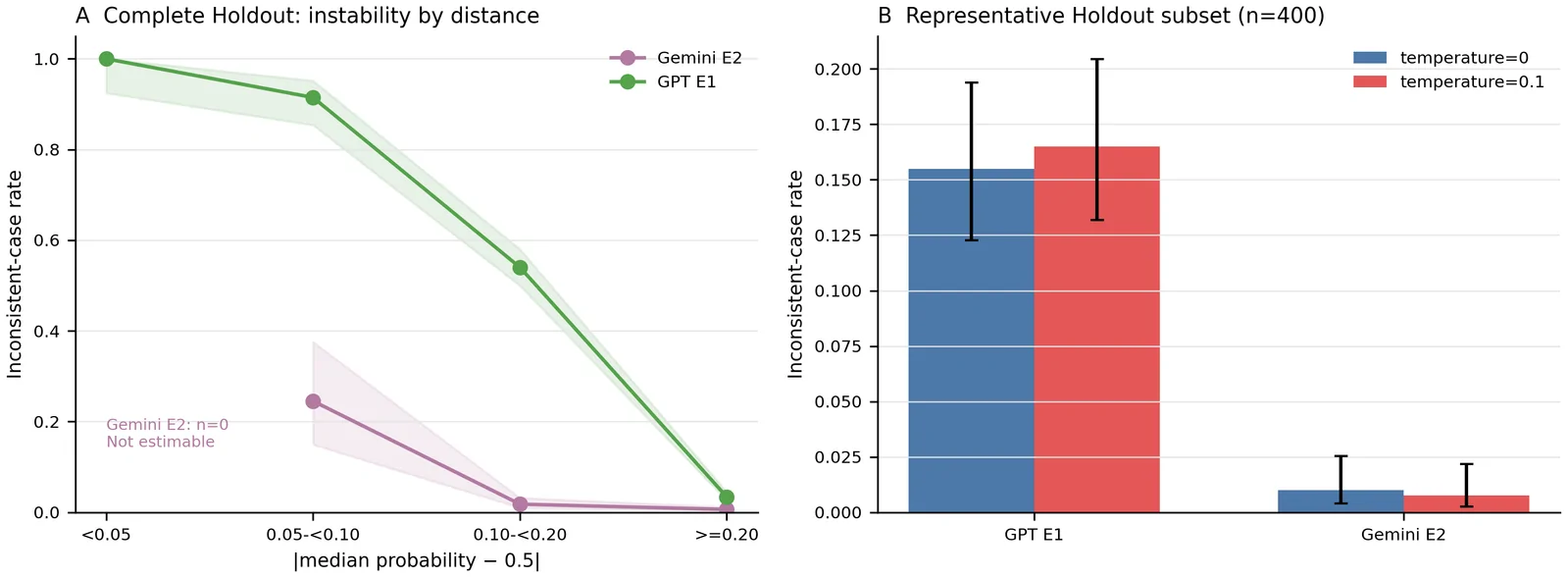
Repeated-output stability of GPT E1 and Gemini E2. **(A)** shows complete-Holdout inconsistent-case rates by the distance between each participant’s median probability and 0.5. The Gemini E2 group at distance<0.05 contained no participants, so its rate and interval were not estimated. **(B)** compares inconsistent-case rates at temperature=0 and temperature=0.1 in the same prevalence-representative Holdout subset. The 400-case subset was proportionally stratified by observed SDS screen-positive status and sex. Shading and error bars show Wilson intervals. **Supplementary Table S11** reports paired temperature differences, probability-margin review, and repeated-call majority-vote analyses.

In the representative 400-case subset, GPT E1 inconsistency was 15.5% at temperature=0 and 16.5% at temperature=0.1. The paired difference was −1.0 percentage point (95% CI −4.3 to 2.3). Gemini E2 inconsistency was 1.0% and 0.8%, with a paired difference of 0.25 percentage point (−1.0 to 1.5). Minimal-temperature decoding therefore did not clearly eliminate GPT E1 variability under the hosted interface.

GPT E1 instability was concentrated near the decision boundary. All 47 of 47 cases within 0.05 of 0.5 were inconsistent, as were 118 of 129 cases at a distance of 0.05-<0.10. By comparison, 51 of 1,497 cases at a distance ≥0.20 were inconsistent. Inconsistency was 30.6% among SDS screen-positive students and 20.8% among SDS screen-negative students, with similar rates by sex. Across the April and August 2026 temperature=0.1 call windows, label agreement was 96.8% for GPT E1 and 98.3% for Gemini E2. Probability Spearman correlations were 0.991 and 0.983, respectively. **Figure 7** displays the threshold-distance and temperature comparisons.

Exploratory probability-margin review demonstrated a marked coverage–stability trade-off for GPT E1. A 0.10 margin deferred 176 of 2,258 cases (7.8%) and captured 165 of 532 inconsistent cases (31.0%), whereas a 0.20 margin deferred 761 cases (33.7%) and captured 481 inconsistent cases (90.4%). Ten-call majority voting yielded a macro-F1 of 0.7343, compared with a mean single-call value of 0.7296 (difference 0.0046, 95% CI −0.0015 to 0.0110). For Gemini E2, the corresponding difference was 0.0014 (95% CI 0.0002 to 0.0025). Complete mitigation results are reported in **Supplementary Tables S11D, E**.

### 3.10 Exploratory sex subgroups and per-1,000 screening consequences

Sex-stratified estimates varied by model and metric rather than showing uniform parity. At 0.5 on Test, LR sensitivity was 0.082 higher among female students (95% CI 0.013-0.157), whereas specificity was 0.070 lower (−0.116 to −0.031). GPT E1 specificity was 0.066 lower among female students (−0.102 to −0.026). On Holdout, GPT E1 sensitivity was 0.128 higher among female students (0.055-0.210). **Supplementary Table S12** reports subgroup n, prevalence, performance, calibration, validity, and differences.

At 0.5, projected outcomes per 1,000 Holdout students were 183 true positives, 129 false positives, and 97 missed positives for GPT E1. The corresponding counts were 202, 147, and 78 for Gemini E2; 219, 202, and 60 for LR; and 74, 17, and 205 for XGBoost. At its Development-derived Youden threshold, XGBoost yielded 245 true positives, 303 false positives, and 34 missed positives. This threshold increased the number sent for review from 92 to 549 per 1,000. The model-threshold pair with the lowest illustrative loss varied with the assumed false-negative:false-positive cost ratio. **Supplementary Table S13** reports Test and Holdout scenarios at ratios of 1:1, 2:1, 5:1, and 10:1.

## 4 Discussion

### 4.1 Main findings

This study evaluated prediction of an SDS standard-score threshold rather than clinical depression. Self-report cutoffs require cautious interpretation and cannot replace a diagnostic interview (48–50). Within this boundary, the study provides a multilayer evaluation of LLM prompt conditions applied to structured questionnaire profiles, rather than a transient ranking of commercial model versions.

Threshold choice materially altered model comparisons. XGBoost’s very low sensitivity at 0.5 largely reflected its conservative isotonic score distribution. Its Development-derived Youden threshold substantially increased sensitivity but also false positives, whereas the fixed-specificity operating point yielded competitive macro-F1. GPT E1 lost macro-F1 at its Development-derived threshold, whereas Gemini E2 retained 0.5. Thus, a common 0.5 cutoff alone did not support a fair claim of LLM superiority. However, no alternative threshold rule identified a universal winner.

Paired uncertainty further weakened point-estimate rankings. For fixed-0.5 Test macro-F1, pairwise differences among the three leading LLM conditions were small and imprecise; all corresponding 95% confidence intervals included 0. Rank frequencies indicated selection variability, and the Development-derived rule favored a different prompt condition.

Probability quality yielded a different comparison. XGBoost had the lowest Brier score and ECE across binning methods. Calibration intercepts, however, indicated substantial systematic miscalibration for LR and both LLM finalists, and GPT E1 had a slope below one on Holdout. A model may therefore support a useful thresholded decision in one cohort whereas its reported probabilities remain unsuitable for risk communication or prioritization without further calibration and validation. For GPT E1 and Gemini E2, the raw model-reported probabilities should therefore be interpreted as model scores rather than calibrated estimates of clinical risk. Any future probability-based use would require *post-hoc* calibration on separate development data, such as logistic scaling or isotonic regression, followed by independent validation.

Scale correlations confirmed shared psychological content between the outcome and predictors. SAS showed the strongest association with SDS, and removing SAS or the non-depression SCL-90 group often worsened AUROC or Brier score. However, no single group explained all predictive performance, and some hard-label changes reversed after threshold selection. The near-chance demographics-only AUROCs further showed that the observed performance was derived mainly from psychometric questionnaire information rather than sex and age. These findings indicate predictive redundancy and construct overlap, not causal attribution. The task is therefore better described as a cross-questionnaire SDS-threshold prediction benchmark than as the discovery of an independent depression marker.

Repeated-call behavior imposed a separate limitation. Gemini E2 was comparatively stable, whereas GPT E1 changed decisions for almost one quarter of complete-Holdout cases and remained variable at temperature=0. Instability was concentrated near 0.5, as expected for thresholded probabilities, but the operational consequence remains important. Such variation can alter who is flagged and increase review burden. The exploratory mitigation analyses showed that probability-margin review could capture most unstable GPT E1 cases only by deferring approximately one-third of the cohort, whereas the 0.10 margin captured fewer than one-third of unstable cases. Majority voting produced only a small change in macro-F1. These strategies may manage selected consequences of instability, but they do not remove the underlying dependence on repeated hosted-model calls. Hosted-model stability should therefore be measured for the exact prompt, decoding configuration, and call window, rather than inferred from macro-F1 or model brand.

### 4.2 Related work and contribution boundary

The present task differs from LLM analyses of social-media language for suicide risk and clinical-interview text used to infer PHQ-8 responses (46, 47). Social-media data raise concerns about contextual collapse, consent, surveillance, and high-consequence false alarms for suicide risk. Clinical interviews contain dialogue, symptom narratives, and interviewer context but require clinical data governance. By contrast, our inputs were consented, de-identified structured questionnaire values rendered as text. This format reduces linguistic ambiguity but retains construct overlap and questionnaire burden. These distinctions define the contribution and preclude direct performance comparisons across incompatible tasks.

More broadly, an LLM benchmark for structured questionnaires requires the same scrutiny of thresholds, calibration, transportability, and subgroups as a conventional prediction model. It additionally requires audits of prompts, parsing, backends, and repeated generation. The present comparisons did not establish that this added representation and generation complexity improved discrimination or probability quality relative to LR or XGBoost. This contribution extends beyond model-version ranking and aligns with transparent prediction-model reporting principles (51). It is also consistent with evidence that input representation and prompt perturbation can alter LLM classification behavior (52, 53).

E1 and E2 should therefore remain labels for bundled prompt conditions. Their higher mean fixed-0.5 macro-F1 cannot be attributed solely to adding two or four examples. Task constraints, balanced demonstrations, output formatting, and probability anchors changed together. Isolating the effect of exemplar number would require a factorial prompt ablation.

### 4.3 Practical implications

The current approach provides no demonstrated reduction in time, item count, or personnel burden relative to direct administration of the 20-item SDS. It requires 126 non-SDS predictor items and should not be presented as a shorter screening instrument. Its defensible immediate use is offline methodological evaluation or secondary analysis when the predictor questionnaires have already been collected for another justified purpose.

Even in this narrow setting, LLM output should not automatically trigger a clinical label or intervention. The evidence field was retained for audit but was not validated as an explanation. A prospective workflow at a Chinese university would require qualified mental-health professionals to define the action threshold and review flagged cases. It would also require appropriate consent, protection of transmitted data, and referral for assessment rather than model-based diagnosis. Questionnaire-based LLM prediction could eventually be evaluated as one component of a broader AI-supported intake or decision-support workflow that combines structured questionnaires with conversational or other clinically relevant information. Such integration would require empirical validation, human oversight, privacy safeguards, and ethical governance before clinical implementation (54).

The per-1,000 scenarios make the threshold trade-off explicit. A highly sensitive rule reduces missed positives but may refer several hundred additional students for review. A conservative rule reduces false positives but can miss most screen-positive cases. Because the false-negative:false-positive cost ratios were illustrative, these analyses support transparent planning rather than claims of net clinical benefit. Operational threshold selection requires empirical preferences, service-capacity estimates, and downstream outcomes (55–57).

### 4.4 Limitations and future research

First, SDS≥53 is a self-report screening threshold rather than a clinician-confirmed disorder, and the retained SAS and SCL-90 variables overlap with general distress and depressive symptoms. Correlation and ablation analyses characterize this overlap but cannot eliminate it. Because item-level questionnaire responses were unavailable in the analytical dataset, sample-specific Cronbach’s alpha and McDonald’s omega could not be calculated. All three cohorts were drawn from one university during a single survey period. Large-age SMDs reflected the grade-cohort design, whereas outcome prevalence and several psychological predictors also shifted. Holdout therefore provides independent within-institution cohort validation, not external validation. Future studies should pair predictions with blinded psychiatric or clinical-psychological assessments and evaluate alternative thresholds and outcomes without equating cross-scale agreement with diagnosis. Multi-university, multi-region, and temporal validation is also required before broader generalization to Chinese university students (58, 59).

Second, model-specific thresholds were selected in a Development-validation subset of 391 students, including 64 screen-positive cases. Evaluation-cohort bootstrapping treated these thresholds as fixed and did not propagate selection uncertainty. Accordingly, the analyses assess prespecified threshold rules rather than precise deployable cutoffs. Reproducibility is further limited because hosted commercial models, OpenRouter routing, model slugs, backend implementations, and response formats may change. Across the supplementary LLM analyses, eight outputs remained invalid after the prespecified parsing and retry procedures. Call timestamps, request metadata, file hashes, and recovery procedures improve reproducibility but cannot freeze an external backend. The findings therefore apply to the evaluated configurations and should not be interpreted as permanent product rankings.

Third, the fairness audit was restricted to sex and did not assess intersectional groups, socioeconomic status, discipline, disability, or other relevant characteristics. The observed female–male differences alone establish neither discrimination nor parity. The benchmark also excluded locally developed Chinese LLMs, supervised fine-tuning, distillation, prospective human review, and a clinical reference standard. Future studies should compare locally available models and evaluate fine-tuning or distillation in separately trained, externally validated pipelines (60–63). They should extend subgroup audits and examine human factors, data governance, and professional review (64, 65).

## 5 Conclusion

In this single-institution SDS-threshold benchmark, several LLM prompt conditions were competitive with LR and XGBoost at specific operating points. The evidence did not show a stable or general LLM advantage. Conclusions varied with threshold rules, XGBoost retained better probability quality, and GPT E1 showed substantial repeated-call instability.

The task also relied on overlapping psychological scales and did not reduce questionnaire burden. Holdout supported only within-institution grade-cohort transport, and the sex analyses were exploratory. No result established clinical diagnosis, clinical utility, fairness, or external validity.

A credible evaluation of LLMs on structured mental-health questionnaires should report discrimination, calibration, Development-derived and prespecified operating points, and paired uncertainty. It should also assess structured-output validity, repeated-call stability, construct overlap, data shift, subgroup performance, and review consequences. These dimensions should remain distinct rather than being collapsed into macro-F1.

Prospective use should be considered only after true external and clinical validation, locally defined action thresholds, privacy and platform governance, and qualified professional oversight. Until then, these findings are best understood as a documented and auditable offline methodological benchmark.

## Data Availability

The raw data supporting the conclusions of this article will be made available by the authors, without undue reservation.
