# Supplementary material for: SDS screen-positive prediction from structured questionnaires in university students: a comparative evaluation of large language model prompting strategies
Source: Front Psychiatry. 2026 Oct 19;17:1942164. doi: 10.3389/fpsyt.2026.1942164 (PMC13724331; doi:10.3389/fpsyt.2026.1942164)
Supplement: Supplementary file 1 [file SupplementaryFile1.docx]

**Supplementary Materials**

**SDS Screen-Positive Prediction From Structured Questionnaires in University Students: A Comparative Evaluation of Large Language Model Prompting Strategies**

Supplementary Tables S1-S13 and Supplementary Figures S1-S7

**Contents**

| **Item** | **Content** |
| --- | --- |
| Table S1 | Frozen run configuration, software environment, model-selection outputs, and API request accounting |
| Table S2 | Single-run estimates and bootstrap confidence intervals |
| Table S3 | Primary repeated-output stability point estimates |
| Table S4 | Exact prompts, exemplars, model settings, parsing/retry rules, and hashes |
| Table S5 | Synthetic illustrative LLM input/output examples |
| Table S6 | Threshold and fixed operating-point analyses |
| Table S7 | Paired bootstrap differences and LLM ranking stability |
| Table S8 | Scale correlations and grouped ablations |
| Table S9 | Cohort distributions and shift measures |
| Table S10 | Enhanced calibration results |
| Table S11 | Repeated-output stability and backend-drift audit |
| Table S12 | Exploratory sex-subgroup audit |
| Table S13 | Per-1,000 screening and error-cost scenarios |
| Figure S1 | Scale correlation matrix |
| Figure S2 | Grouped scale-ablation effects |
| Figure S3 | Cohort-shift heatmap |
| Figure S4 | Test calibration and probability distributions |
| Figure S5 | Holdout calibration and probability distributions |
| Figure S6 | ECE binning sensitivity |
| Figure S7 | Threshold robustness |

**Table S1.** Frozen run configuration, software environment, model-selection outputs, and API request accounting

| **Item** | **Value** |
| --- | --- |
| Primary analysis run identifier | 20260422_145804 |
| Random seed | 42 |
| Primary label | SDS standard score >= 53 |
| Development/Test/Holdout sample sizes | 1951 / 2186 / 2258 |
| Missingness in primary predictor inventory | Development: 0; Test: 0; Holdout: 0 missing values in the primary predictor inventory |
| Conda environment | mental-llm |
| Python version | 3.10.0 |
| Operating system | Windows-10-10.0.19045-SP0 |
| Key package versions | scikit-learn 1.7.2; xgboost 3.2.0; pandas 2.3.3; numpy 2.2.6; scipy 1.15.3; matplotlib 3.10.8 |
| LR hyperparameter selection | Development-training cross-validation |
| LR final parameter | C=0.1 |
| XGBoost hyperparameter selection | Development-training cross-validation |
| XGBoost final parameters | n_estimators=500; max_depth=2; learning_rate=0.05; subsample=0.8; colsample_bytree=1.0; min_child_weight=3; reg_lambda=1.0 |
| XGBoost calibration selection/fitting | Development-validation |
| XGBoost calibration method | isotonic calibration |
| Development-validation Brier score, isotonic | 0.106865 |
| Development-validation Brier score, sigmoid | 0.116083 |
| LLM model slugs | openai/gpt-5.4-mini; anthropic/claude-haiku-4.5; google/gemini-3-flash-preview |
| Prompt families | A, B, E1, E2 |
| Temperature | 0.1 |
| top_p | 1.0 |
| max_tokens | 200 |
| Reasoning setting | GPT: reasoning effort none; non-GPT models: reasoning disabled |
| Response format | JSON object |
| Configuration SHA-256 | 5e2308860fd1712a40053d3efaf625f32858475405ca21df9418050e7bdb416d |
| Prompt-assets SHA-256 | 2e63ea32e4b432ed282edb992e72d8776225f64ee26fc2aaca6a6765ddb83866 |
| Provider-side API requests, total | 122,051 requests, including retries and resumed calls; not used as an analytic denominator |
| Provider-side API requests by model | anthropic/claude-haiku-4.5: 13,557; google/gemini-3-flash-preview: 49,699; openai/gpt-5.4-mini: 58,795 |
| Finalized LLM output rows used for metric computation | 90,308 finalized output rows across single-run and repeated-call analyses |
| API request accounting | Provider-side request totals exceeded finalized analytical outputs because retries and interrupted calls generated additional requests; all metrics were calculated exclusively from finalized prediction files. |

***Note.*** *Configuration details reflect the frozen analytical settings and retained software environment. Provider-side API request totals include retries and interrupted calls and are not analytic denominators.*

**Table S2.** Complete point estimates and bootstrap 95% confidence intervals for single-run performance, probability, calibration, and output-validity metrics

| **Condition** | **Split** | **Acc. (95% CI)** | **Sens. (95% CI)** | **Spec. (95% CI)** | **Prec. (95% CI)** | **Macro-F1 (95% CI)** | **AUROC (95% CI)** | **AUPRC (95% CI)** | **Brier (95% CI)** | **ECE (95% CI)** | **Parse-and-validation success rate (95% CI)** | **Invalid output rate (95% CI)** |
| --- | --- | --- | --- | --- | --- | --- | --- | --- | --- | --- | --- | --- |
| LR | Test | 0.748 (0.729-0.765) | 0.785 (0.750-0.817) | 0.737 (0.715-0.756) | 0.491 (0.458-0.522) | 0.710 (0.689-0.728) | 0.844 (0.824-0.862) | 0.701 (0.664-0.736) | 0.174 (0.165-0.183) | 0.204 (0.190-0.219) | N/A | N/A |
| XGBoost | Test | 0.817 (0.802-0.833) | 0.316 (0.279-0.359) | 0.979 (0.972-0.986) | 0.833 (0.781-0.883) | 0.674 (0.649-0.701) | 0.846 (0.829-0.865) | 0.639 (0.600-0.677) | 0.129 (0.120-0.138) | 0.045 (0.034-0.061) | N/A | N/A |
| GPT E1 | Test | 0.786 (0.768-0.804) | 0.691 (0.653-0.730) | 0.817 (0.798-0.836) | 0.550 (0.510-0.587) | 0.732 (0.709-0.753) | 0.818 (0.793-0.839) | 0.652 (0.614-0.689) | 0.157 (0.148-0.167) | 0.141 (0.127-0.157) | 1.0000 (1.0000-1.0000) | 0.0000 (0.0000-0.0000) |
| Gemini E2 | Test | 0.776 (0.758-0.794) | 0.742 (0.704-0.778) | 0.788 (0.767-0.808) | 0.530 (0.496-0.565) | 0.730 (0.709-0.751) | 0.834 (0.812-0.853) | 0.645 (0.605-0.683) | 0.167 (0.158-0.176) | 0.187 (0.173-0.202) | 0.9982 (0.9959-1.0000) | 0.0018 (0.0000-0.0041) |
| GPT E2 | Test | 0.785 (0.766-0.802) | 0.680 (0.643-0.719) | 0.818 (0.800-0.836) | 0.548 (0.511-0.586) | 0.729 (0.709-0.749) | 0.826 (0.803-0.847) | 0.656 (0.616-0.694) | 0.153 (0.143-0.162) | 0.127 (0.114-0.143) | 1.0000 (1.0000-1.0000) | 0.0000 (0.0000-0.0000) |
| Claude B | Test | 0.819 (0.803-0.834) | 0.449 (0.411-0.492) | 0.939 (0.927-0.950) | 0.704 (0.654-0.752) | 0.718 (0.694-0.740) | 0.783 (0.759-0.805) | 0.523 (0.484-0.562) | 0.163 (0.157-0.169) | 0.134 (0.120-0.150) | 1.0000 (1.0000-1.0000) | 0.0000 (0.0000-0.0000) |
| Claude E1 | Test | 0.753 (0.734-0.771) | 0.781 (0.744-0.816) | 0.745 (0.723-0.766) | 0.497 (0.464-0.530) | 0.714 (0.692-0.734) | 0.822 (0.800-0.842) | 0.621 (0.582-0.657) | 0.178 (0.170-0.187) | 0.201 (0.187-0.216) | 1.0000 (1.0000-1.0000) | 0.0000 (0.0000-0.0000) |
| Claude E2 | Test | 0.758 (0.740-0.777) | 0.738 (0.700-0.775) | 0.765 (0.743-0.786) | 0.504 (0.468-0.539) | 0.713 (0.691-0.735) | 0.816 (0.794-0.838) | 0.612 (0.573-0.648) | 0.175 (0.165-0.184) | 0.183 (0.169-0.198) | 1.0000 (1.0000-1.0000) | 0.0000 (0.0000-0.0000) |
| GPT A | Test | 0.769 (0.749-0.785) | 0.631 (0.590-0.672) | 0.813 (0.793-0.831) | 0.522 (0.484-0.560) | 0.706 (0.684-0.729) | 0.798 (0.773-0.820) | 0.591 (0.550-0.634) | 0.168 (0.158-0.178) | 0.134 (0.121-0.151) | 0.9995 (0.9986-1.0000) | 0.0005 (0.0000-0.0014) |
| GPT B | Test | 0.731 (0.712-0.748) | 0.725 (0.687-0.763) | 0.732 (0.712-0.752) | 0.467 (0.431-0.501) | 0.686 (0.664-0.707) | 0.789 (0.765-0.812) | 0.566 (0.523-0.609) | 0.176 (0.168-0.185) | 0.162 (0.147-0.178) | 1.0000 (1.0000-1.0000) | 0.0000 (0.0000-0.0000) |
| Gemini E1 | Test | 0.712 (0.693-0.732) | 0.800 (0.766-0.834) | 0.684 (0.662-0.707) | 0.450 (0.419-0.480) | 0.679 (0.658-0.700) | 0.839 (0.817-0.858) | 0.656 (0.617-0.694) | 0.195 (0.186-0.204) | 0.242 (0.228-0.258) | 0.9776 (0.9712-0.9835) | 0.0224 (0.0165-0.0288) |
| Claude A | Test | 0.777 (0.760-0.793) | 0.242 (0.206-0.279) | 0.950 (0.939-0.961) | 0.611 (0.545-0.676) | 0.606 (0.581-0.631) | 0.624 (0.603-0.646) | 0.370 (0.337-0.408) | 0.173 (0.163-0.185) | 0.061 (0.046-0.078) | 1.0000 (1.0000-1.0000) | 0.0000 (0.0000-0.0000) |
| Gemini A | Test | 0.562 (0.541-0.583) | 0.897 (0.871-0.923) | 0.453 (0.429-0.478) | 0.347 (0.323-0.370) | 0.555 (0.535-0.577) | 0.814 (0.791-0.835) | 0.597 (0.557-0.636) | 0.294 (0.282-0.306) | 0.361 (0.346-0.377) | 0.9876 (0.9826-0.9922) | 0.0124 (0.0078-0.0174) |
| Gemini B | Test | 0.518 (0.497-0.540) | 0.878 (0.850-0.906) | 0.402 (0.379-0.426) | 0.322 (0.300-0.346) | 0.514 (0.494-0.536) | 0.818 (0.794-0.840) | 0.618 (0.577-0.657) | 0.286 (0.275-0.298) | 0.354 (0.338-0.372) | 0.9360 (0.9254-0.9456) | 0.0640 (0.0544-0.0746) |
| LR | Holdout | 0.738 (0.720-0.755) | 0.784 (0.751-0.816) | 0.720 (0.699-0.740) | 0.521 (0.488-0.551) | 0.712 (0.692-0.731) | 0.832 (0.813-0.851) | 0.710 (0.676-0.745) | 0.179 (0.171-0.188) | 0.187 (0.171-0.203) | N/A | N/A |
| XGBoost | Holdout | 0.778 (0.762-0.795) | 0.266 (0.232-0.299) | 0.976 (0.968-0.983) | 0.812 (0.755-0.866) | 0.632 (0.609-0.655) | 0.835 (0.818-0.853) | 0.636 (0.600-0.672) | 0.147 (0.138-0.156) | 0.062 (0.047-0.079) | N/A | N/A |
| GPT E1 | Holdout | 0.774 (0.757-0.791) | 0.655 (0.617-0.692) | 0.821 (0.802-0.840) | 0.586 (0.550-0.621) | 0.729 (0.711-0.749) | 0.808 (0.788-0.830) | 0.668 (0.630-0.707) | 0.160 (0.151-0.168) | 0.113 (0.097-0.129) | 1.0000 (1.0000-1.0000) | 0.0000 (0.0000-0.0000) |
| Gemini E2 | Holdout | 0.775 (0.759-0.793) | 0.723 (0.689-0.756) | 0.796 (0.776-0.815) | 0.579 (0.546-0.612) | 0.739 (0.720-0.758) | 0.820 (0.801-0.839) | 0.664 (0.628-0.698) | 0.167 (0.158-0.175) | 0.151 (0.135-0.167) | 0.9991 (0.9978-1.0000) | 0.0009 (0.0000-0.0022) |

***Note.*** *Cells report point estimate (bootstrap 95% CI). The table covers all evaluated single-run conditions: all LLM conditions and ML baselines on Test, plus ML baselines and Test-selected LLM finalists on Holdout. Structured-output validity metrics apply to LLM outputs; ML rows are marked N/A. Parse-and-validation success rate is the operational parse_success indicator described in Methods. Repeated-output stability metrics are not included here because they are reported as point estimates only in Supplementary Table S3. Acc. = accuracy; Sens. = sensitivity; Spec. = specificity; Prec. = precision; ECE = expected calibration error.*

**Table S3.** Repeated-output stability point estimates

| **Condition** | **Stability set** | **Case count** | **Inconsistent cases, n/N (%)** | **Mean vote margin** | **Mode Macro-F1** | **Prediction entropy** |
| --- | --- | --- | --- | --- | --- | --- |
| Claude A | Test subset | 120 | 3/120 (2.50%) | 0.988 | 0.557 | 0.019 |
| Claude B | Test subset | 120 | 2/120 (1.67%) | 0.995 | 0.652 | 0.010 |
| Claude E1 | Test subset | 120 | 0/120 (0.00%) | 1.000 | 0.775 | 0.000 |
| Claude E2 | Test subset | 120 | 0/120 (0.00%) | 1.000 | 0.800 | 0.000 |
| GPT A | Test subset | 120 | 42/120 (35.00%) | 0.843 | 0.783 | 0.250 |
| GPT B | Test subset | 120 | 50/120 (41.67%) | 0.807 | 0.783 | 0.301 |
| GPT E1 | Test subset | 120 | 38/120 (31.67%) | 0.867 | 0.800 | 0.214 |
| GPT E2 | Test subset | 120 | 17/120 (14.17%) | 0.923 | 0.792 | 0.109 |
| Gemini A | Test subset | 120 | 9/120 (7.50%) | 0.983 | 0.665 | 0.034 |
| Gemini B | Test subset | 120 | 21/120 (17.50%) | 0.952 | 0.650 | 0.076 |
| Gemini E1 | Test subset | 120 | 19/120 (15.83%) | 0.963 | 0.774 | 0.071 |
| Gemini E2 | Test subset | 120 | 3/120 (2.50%) | 0.992 | 0.775 | 0.015 |
| GPT E1 | Holdout finalist | 2258 | 532/2258 (23.56%) | 0.885 | 0.734 | 0.172 |
| Gemini E2 | Holdout finalist | 2258 | 34/2258 (1.51%) | 0.995 | 0.739 | 0.009 |

***Note.*** *Point estimates only. Test stability uses the balanced Test subset (n=120 per LLM condition); Holdout finalist stability uses the full Holdout set (n=2258 per finalist). Each stability analysis uses 10 repeated runs. Vote-based metrics use the binary evaluation labels recorded for each repeat; invalid outputs contribute to inconsistent cases as described in Methods.*

**Table S4.** Exact LLM prompt conditions, frozen exemplar materials, model settings, parsing and retry rules, and reproducibility file index.

**Panel A. Twelve evaluated LLM model-prompt conditions**

| **Condition** | **OpenRouter model slug** | **Prompt** | **Prompt components** | **Temperature** | **top_p** | **Max tokens** |
| --- | --- | --- | --- | --- | --- | --- |
| GPT A | openai/gpt-5.4-mini | A | Direct task instruction and JSON schema; no exemplars. | 0.1 | 1.0 | 200 |
| GPT B | openai/gpt-5.4-mini | B | Explicit SDS>=53 outcome definition, uncertainty guidance, and JSON schema; no exemplars. | 0.1 | 1.0 | 200 |
| GPT E1 | openai/gpt-5.4-mini | E1 | Two class-balanced demonstrations (negative and positive medoids), output-format demonstrations, and 0.20/0.80 probability anchors. | 0.1 | 1.0 | 200 |
| GPT E2 | openai/gpt-5.4-mini | E2 | Four class-balanced demonstrations (medoid and diverse example per class), output-format demonstrations, and 0.20/0.80 probability anchors. | 0.1 | 1.0 | 200 |
| Claude A | anthropic/claude-haiku-4.5 | A | Direct task instruction and JSON schema; no exemplars. | 0.1 | 1.0 | 200 |
| Claude B | anthropic/claude-haiku-4.5 | B | Explicit SDS>=53 outcome definition, uncertainty guidance, and JSON schema; no exemplars. | 0.1 | 1.0 | 200 |
| Claude E1 | anthropic/claude-haiku-4.5 | E1 | Two class-balanced demonstrations (negative and positive medoids), output-format demonstrations, and 0.20/0.80 probability anchors. | 0.1 | 1.0 | 200 |
| Claude E2 | anthropic/claude-haiku-4.5 | E2 | Four class-balanced demonstrations (medoid and diverse example per class), output-format demonstrations, and 0.20/0.80 probability anchors. | 0.1 | 1.0 | 200 |
| Gemini A | google/gemini-3-flash-preview | A | Direct task instruction and JSON schema; no exemplars. | 0.1 | 1.0 | 200 |
| Gemini B | google/gemini-3-flash-preview | B | Explicit SDS>=53 outcome definition, uncertainty guidance, and JSON schema; no exemplars. | 0.1 | 1.0 | 200 |
| Gemini E1 | google/gemini-3-flash-preview | E1 | Two class-balanced demonstrations (negative and positive medoids), output-format demonstrations, and 0.20/0.80 probability anchors. | 0.1 | 1.0 | 200 |
| Gemini E2 | google/gemini-3-flash-preview | E2 | Four class-balanced demonstrations (medoid and diverse example per class), output-format demonstrations, and 0.20/0.80 probability anchors. | 0.1 | 1.0 | 200 |

**Panel B. Calling, parsing, scoring, and structured-data model specifications**

| **Item** | **Frozen specification** |
| --- | --- |
| Access layer | OpenRouter chat-completions API; api_base=https://openrouter.ai/api/v1 |
| Response constraint | response_format={"type":"json_object"}; required output keys: label, prob_positive, evidence. |
| Reasoning mode | disabled; GPT reasoning_effort=none. |
| Timeout and retry | Timeout 120 s; 2 retries after the initial attempt. |
| Limited parsing | Attempt strict JSON; if needed remove markdown fences, isolate the outermost JSON object, normalize smart quotes, remove trailing commas, and use relaxed JSON loading. |
| Probability normalization | Accept numeric values or percentage strings; values >1 and <=100 are divided by 100; values outside [0,1] or nonnumeric values are invalid. |
| Formal hard label | Regenerated from normalized prob_positive>=0.5; the model-reported label is retained only for audit. |
| Invalid-output handling | Invalid outputs are errors in failure-penalized hard-label metrics and are excluded from probability metrics; valid denominators are reported. |
| LR final model | C=0.1; L2; class_weight=balanced; sex mode-imputed and one-hot encoded; continuous variables median-imputed and standardized. |
| XGBoost final model | n_estimators=500; max_depth=2; learning_rate=0.05; subsample=0.8; colsample_bytree=1.0; min_child_weight=3; reg_lambda=1.0; isotonic calibration. |

**Panel C. Exact common system prompt**

| **Frozen asset** | **Verbatim text** |
| --- | --- |
| system_prompt_common_v1.txt | You are assisting a research benchmark on questionnaire-based mental health screening. Use only the participant variables provided in the prompt. Do not infer missing information, do not use external knowledge, and do not treat the task as a clinical diagnosis. Return only valid JSON with the keys label, prob_positive, and evidence. |

**Panel D. Exact user prompt A**

| **Frozen asset** | **Verbatim text** |
| --- | --- |
| prompt_A_user_v1.txt | [Task] Predict whether the following participant is SDS screen-positive in this study.  [Output] Return only JSON in the form: {{"label":"positive\|negative","prob_positive":0.000,"evidence":["<short phrase>","<short phrase>"]}}  [Rules] - Use only the participant profile shown below. - Do not mention diagnosis. - Do not output any text outside the JSON object.  [Participant Profile] sex: {sex} age_years: {age_years_int} sas_std: {sas_std} cfq_total: {cfq_total} aaq2_total: {aaq2_total} scl90_somatization: {scl90_somatization} scl90_obsessive_compulsive: {scl90_obsessive_compulsive} scl90_interpersonal_sensitivity: {scl90_interpersonal_sensitivity} scl90_anxiety: {scl90_anxiety} scl90_hostility: {scl90_hostility} scl90_phobic_anxiety: {scl90_phobic_anxiety} scl90_paranoid_ideation: {scl90_paranoid_ideation} scl90_psychoticism: {scl90_psychoticism} scl90_additional_items: {scl90_additional_items} |

**Panel E. Exact user prompt B**

| **Frozen asset** | **Verbatim text** |
| --- | --- |
| prompt_B_user_v1.txt | [Task] Estimate the probability that the following participant would have a standardized SDS score greater than or equal to 53 and therefore be classified as SDS screen-positive in this study.  [Output] Return only JSON in the form: {{"label":"positive\|negative","prob_positive":0.000,"evidence":["<short phrase>","<short phrase>"]}}  [Rules] - Use only the non-SDS variables shown below. - Do not infer missing information. - This is a screening prediction task, not a clinical diagnosis. - If the evidence is mixed, reflect uncertainty in prob_positive instead of fabricating facts. - The final label must correspond to whether prob_positive is at least 0.5. - Do not output any text outside the JSON object.  [Participant Profile] sex: {sex} age_years: {age_years_int} sas_std: {sas_std} cfq_total: {cfq_total} aaq2_total: {aaq2_total} scl90_somatization: {scl90_somatization} scl90_obsessive_compulsive: {scl90_obsessive_compulsive} scl90_interpersonal_sensitivity: {scl90_interpersonal_sensitivity} scl90_anxiety: {scl90_anxiety} scl90_hostility: {scl90_hostility} scl90_phobic_anxiety: {scl90_phobic_anxiety} scl90_paranoid_ideation: {scl90_paranoid_ideation} scl90_psychoticism: {scl90_psychoticism} scl90_additional_items: {scl90_additional_items} |

**Panel F. Exact user prompt E1**

| **Frozen asset** | **Verbatim text** |
| --- | --- |
| prompt_E1_user_v1.txt | [Task] Estimate the probability that the target participant would have a standardized SDS score greater than or equal to 53 and therefore be classified as SDS screen-positive in this study.  [Output] Return only JSON in the form: {{"label":"positive\|negative","prob_positive":0.000,"evidence":["<short phrase>","<short phrase>"]}}  [Rules] - Use only the non-SDS variables shown below. - This is a screening prediction task, not a clinical diagnosis. - Follow the examples' output format and decision style, but judge the target participant independently from the target profile. - The example probabilities (0.20 / 0.80) are output-format and decision-style anchors only; they are not the target participant's true SDS probability and should not be copied mechanically. - If the evidence is mixed, reflect uncertainty in prob_positive instead of fabricating facts. - The final label must correspond to whether prob_positive is at least 0.5. - Do not output any text outside the JSON object.  [Example 1] Participant profile: sex: {ex_neg_medoid.sex} age_years: {ex_neg_medoid.age_years_int} sas_std: {ex_neg_medoid.sas_std} cfq_total: {ex_neg_medoid.cfq_total} aaq2_total: {ex_neg_medoid.aaq2_total} scl90_somatization: {ex_neg_medoid.scl90_somatization} scl90_obsessive_compulsive: {ex_neg_medoid.scl90_obsessive_compulsive} scl90_interpersonal_sensitivity: {ex_neg_medoid.scl90_interpersonal_sensitivity} scl90_anxiety: {ex_neg_medoid.scl90_anxiety} scl90_hostility: {ex_neg_medoid.scl90_hostility} scl90_phobic_anxiety: {ex_neg_medoid.scl90_phobic_anxiety} scl90_paranoid_ideation: {ex_neg_medoid.scl90_paranoid_ideation} scl90_psychoticism: {ex_neg_medoid.scl90_psychoticism} scl90_additional_items: {ex_neg_medoid.scl90_additional_items} Correct output: {{"label":"negative","prob_positive":0.20,"evidence":[]}}  [Example 2] Participant profile: sex: {ex_pos_medoid.sex} age_years: {ex_pos_medoid.age_years_int} sas_std: {ex_pos_medoid.sas_std} cfq_total: {ex_pos_medoid.cfq_total} aaq2_total: {ex_pos_medoid.aaq2_total} scl90_somatization: {ex_pos_medoid.scl90_somatization} scl90_obsessive_compulsive: {ex_pos_medoid.scl90_obsessive_compulsive} scl90_interpersonal_sensitivity: {ex_pos_medoid.scl90_interpersonal_sensitivity} scl90_anxiety: {ex_pos_medoid.scl90_anxiety} scl90_hostility: {ex_pos_medoid.scl90_hostility} scl90_phobic_anxiety: {ex_pos_medoid.scl90_phobic_anxiety} scl90_paranoid_ideation: {ex_pos_medoid.scl90_paranoid_ideation} scl90_psychoticism: {ex_pos_medoid.scl90_psychoticism} scl90_additional_items: {ex_pos_medoid.scl90_additional_items} Correct output: {{"label":"positive","prob_positive":0.80,"evidence":[]}}  [Target Participant] sex: {sex} age_years: {age_years_int} sas_std: {sas_std} cfq_total: {cfq_total} aaq2_total: {aaq2_total} scl90_somatization: {scl90_somatization} scl90_obsessive_compulsive: {scl90_obsessive_compulsive} scl90_interpersonal_sensitivity: {scl90_interpersonal_sensitivity} scl90_anxiety: {scl90_anxiety} scl90_hostility: {scl90_hostility} scl90_phobic_anxiety: {scl90_phobic_anxiety} scl90_paranoid_ideation: {scl90_paranoid_ideation} scl90_psychoticism: {scl90_psychoticism} scl90_additional_items: {scl90_additional_items} |

**Panel G. Exact user prompt E2**

| **Frozen asset** | **Verbatim text** |
| --- | --- |
| prompt_E2_user_v1.txt | [Task] Estimate the probability that the target participant would have a standardized SDS score greater than or equal to 53 and therefore be classified as SDS screen-positive in this study.  [Output] Return only JSON in the form: {{"label":"positive\|negative","prob_positive":0.000,"evidence":["<short phrase>","<short phrase>"]}}  [Rules] - Use only the non-SDS variables shown below. - This is a screening prediction task, not a clinical diagnosis. - Follow the examples' output format and decision style, but judge the target participant independently from the target profile. - The example probabilities (0.20 / 0.80) are output-format and decision-style anchors only; they are not the target participant's true SDS probability and should not be copied mechanically. - If the evidence is mixed, reflect uncertainty in prob_positive instead of fabricating facts. - The final label must correspond to whether prob_positive is at least 0.5. - Do not output any text outside the JSON object.  [Example 1] Participant profile: {negative_medoid_profile_block} Correct output: {{"label":"negative","prob_positive":0.20,"evidence":[]}}  [Example 2] Participant profile: {negative_diverse_profile_block} Correct output: {{"label":"negative","prob_positive":0.20,"evidence":[]}}  [Example 3] Participant profile: {positive_medoid_profile_block} Correct output: {{"label":"positive","prob_positive":0.80,"evidence":[]}}  [Example 4] Participant profile: {positive_diverse_profile_block} Correct output: {{"label":"positive","prob_positive":0.80,"evidence":[]}}  [Target Participant] sex: {sex} age_years: {age_years_int} sas_std: {sas_std} cfq_total: {cfq_total} aaq2_total: {aaq2_total} scl90_somatization: {scl90_somatization} scl90_obsessive_compulsive: {scl90_obsessive_compulsive} scl90_interpersonal_sensitivity: {scl90_interpersonal_sensitivity} scl90_anxiety: {scl90_anxiety} scl90_hostility: {scl90_hostility} scl90_phobic_anxiety: {scl90_phobic_anxiety} scl90_paranoid_ideation: {scl90_paranoid_ideation} scl90_psychoticism: {scl90_psychoticism} scl90_additional_items: {scl90_additional_items} |

**Panel H. Frozen, de-identified exemplar profiles used by E1 and E2**

| **Exemplar** | **Class** | **E1 order** | **E2 order** | **Exact profile block** |
| --- | --- | --- | --- | --- |
| negative_medoid | negative | 1 | 1 | sex: female age_years: 20 sas_std: 38 cfq_total: 28 aaq2_total: 21 scl90_somatization: 1.17 scl90_obsessive_compulsive: 1.8 scl90_interpersonal_sensitivity: 1.44 scl90_anxiety: 1.4 scl90_hostility: 1.17 scl90_phobic_anxiety: 1.57 scl90_paranoid_ideation: 1.17 scl90_psychoticism: 1.3 scl90_additional_items: 1.57 |
| negative_diverse | negative | - | 2 | sex: male age_years: 22 sas_std: 46 cfq_total: 31 aaq2_total: 19 scl90_somatization: 1.5 scl90_obsessive_compulsive: 2.2 scl90_interpersonal_sensitivity: 2.11 scl90_anxiety: 1.7 scl90_hostility: 1.67 scl90_phobic_anxiety: 1.14 scl90_paranoid_ideation: 2.17 scl90_psychoticism: 1.6 scl90_additional_items: 1.14 |
| positive_medoid | positive | 2 | 3 | sex: female age_years: 19 sas_std: 48 cfq_total: 33 aaq2_total: 25 scl90_somatization: 1.92 scl90_obsessive_compulsive: 2.1 scl90_interpersonal_sensitivity: 2.11 scl90_anxiety: 2.2 scl90_hostility: 2 scl90_phobic_anxiety: 1.71 scl90_paranoid_ideation: 1.83 scl90_psychoticism: 1.6 scl90_additional_items: 1.86 |
| positive_diverse | positive | - | 4 | sex: female age_years: 18 sas_std: 43 cfq_total: 46 aaq2_total: 40 scl90_somatization: 1 scl90_obsessive_compulsive: 2.1 scl90_interpersonal_sensitivity: 2.33 scl90_anxiety: 1.7 scl90_hostility: 1.17 scl90_phobic_anxiety: 1.29 scl90_paranoid_ideation: 1.67 scl90_psychoticism: 2 scl90_additional_items: 3.29 |

***Note.*** *Exemplars were selected only from Development-training with seed 42. Selection rule: z-score continuous predictors on development-training, one-hot sex, medoid by minimum distance to class centroid, diverse exemplar by IQR-constrained maximum distance to medoid. Subject hashes are omitted from the supplement; the exact predictor values, labels, and demonstration order are retained above.*

**Panel I. Reproducibility file index and SHA-256 hashes**

| **Repository-relative path** | **SHA-256** | **Bytes** |
| --- | --- | --- |
| prompts/system_prompt_common_v1.txt | e9bc2ef736a4331364d26b9c5ca9dc6bfd1ea6b90c3981ee25918bdcb10e8901 | 335 |
| prompts/prompt_A_user_v1.txt | 52f32bca6b2eef474232d2b6e6063820a2bd9903a65b83d6d800af1963fec18c | 908 |
| prompts/prompt_B_user_v1.txt | 1b4d1f2f38b22f278397133c7fdce56148118dfa6f3eb54bc64342e5f3cf3ffe | 1256 |
| prompts/prompt_E1_user_v1.txt | a479460861c54be2b85e5ec4f702e5177d69271f0b96425d01e0725a424c7e4b | 3188 |
| prompts/prompt_E2_user_v1.txt | 669091cc89b48f9d7afd2d27800f25d5574bc388d793d46d9f62c9047c080a2a | 2098 |
| column_map.yaml | f0055fb5af1a401bafd7ffce2b026ea7a4dfbaa5321d77793c317cae431e39d5 | 962 |
| mental_llm/llm.py | 7f7e8c8aef569e0e3ea6b5e804611cccc6bf08e243b34665c2d49962a88e80aa | 18320 |
| mental_llm/prompts.py | 720e0ec39ed6e209dda7b327579d476e1add4f85ae80e111108c19993d34ebb6 | 9382 |
| mental_llm/ml.py | 138b826ed10c7115c6f2e6b25baa01aed5651ba661bf9510fe3c629cde113f79 | 12381 |
| requirements.txt | 79267d67af226181e52bed544fd22200a5023fa9da48d678617631b4f8d73fa6 | 188 |

***Note.*** *Prompt-condition differences are interpreted as composite prompt-condition effects. E1 and E2 differ in exemplar number, exemplar composition, format demonstrations, and probability anchors; they are not isolated few-shot-count interventions.*

**Table S5.** Synthetic illustrative LLM output examples

**S5-1: Synthetic screen-negative example**

| **Field** | **Value** |
| --- | --- |
| Prompt condition | GPT E1; balanced 2-shot finalist condition |
| Input profile summary | Female; age 20 years; SAS standard score 39; CFQ total 19; AAQ-II total 18; SCL-90 factors generally low (somatization 1.10, obsessive-compulsive 1.30, interpersonal sensitivity 1.20, anxiety 1.10, hostility 1.00, phobic anxiety 1.00, paranoid ideation 1.10, psychoticism 1.00, additional items 1.10). SDS fields, SCL-90 depression, and SCL-90 summary statistics are not provided. |
| Model-style JSON output | {"label":"negative","prob_positive":0.18,"evidence":["synthetic profile has low anxiety and low SCL-90 factor scores","SDS fields and target-proximal depression features are not included"]} |
| Parsing / normalization result | Valid JSON; prob_positive already numeric in [0,1]; no repair required; operationally structured-output-valid = yes. |
| Formal evaluation label | label_eval = negative because normalized prob_positive < 0.5. |
| Note | Format illustration only; not a real participant record; evidence is audit-only and non-clinical. |

**S5-2: Synthetic screen-positive example**

| **Field** | **Value** |
| --- | --- |
| Prompt condition | Gemini E2; balanced 4-shot finalist condition |
| Input profile summary | Male; age 21 years; SAS standard score 61; CFQ total 42; AAQ-II total 35; SCL-90 factors elevated across several non-depression domains (somatization 1.80, obsessive-compulsive 2.40, interpersonal sensitivity 2.30, anxiety 2.60, hostility 2.10, phobic anxiety 2.00, paranoid ideation 2.20, psychoticism 1.90, additional items 2.30). SDS fields, SCL-90 depression, and SCL-90 summary statistics are not provided. |
| Model-style JSON output | {"label":"positive","prob_positive":0.74,"evidence":["synthetic profile has elevated anxiety and multiple SCL-90 factor scores","higher CFQ and AAQ-II totals indicate greater questionnaire-level distress"]} |
| Parsing / normalization result | Valid JSON; prob_positive already numeric in [0,1]; no repair required; operationally structured-output-valid = yes. |
| Formal evaluation label | label_eval = positive because normalized prob_positive >= 0.5. |
| Note | Format illustration only; not a real participant record; evidence is audit-only and non-clinical. |

***Note.*** *The examples are synthetic and are provided only to illustrate the LLM input-output format and JSON evaluation workflow used in the SDS>=53 screen-positive prediction benchmark. They are not real participant records and were not sampled from prediction files. The evidence field was retained for audit purposes only and was not evaluated as a clinically valid explanation, psychological risk report, or human-usable rationale. Formal evaluation labels were regenerated from normalized prob_positive using the fixed 0.5 threshold; model-reported label text was not used directly for scoring.*

**Table S6.** Development-derived thresholds, fixed 0.5 comparisons, and Development-selected fixed-sensitivity and fixed-specificity operating points.

**Panel A. Model-specific Youden thresholds selected only in Development-validation**

| **Condition** | **Rule** | **Locked threshold** | **Sensitivity** | **Specificity** | **Youden J** | **Valid n/N** |
| --- | --- | --- | --- | --- | --- | --- |
| LR | youden | 0.466259536891 | 0.813 | 0.752 | 0.565 | 391/391 |
| XGBoost | youden | 0.173913046718 | 0.844 | 0.630 | 0.474 | 391/391 |
| GPT A | youden | 0.248000000000 | 0.734 | 0.713 | 0.447 | 391/391 |
| GPT B | youden | 0.371000000000 | 0.734 | 0.682 | 0.416 | 391/391 |
| GPT E1 | youden | 0.380000000000 | 0.781 | 0.734 | 0.515 | 391/391 |
| GPT E2 | youden | 0.300000000000 | 0.828 | 0.661 | 0.489 | 391/391 |
| Claude A | youden | 0.250000000000 | 0.266 | 0.902 | 0.168 | 391/391 |
| Claude B | youden | 0.350000000000 | 0.750 | 0.633 | 0.383 | 391/391 |
| Claude E1 | youden | 0.500000000000 | 0.719 | 0.780 | 0.499 | 391/391 |
| Claude E2 | youden | 0.500000000000 | 0.656 | 0.786 | 0.442 | 391/391 |
| Gemini A | youden | 0.785000000000 | 0.797 | 0.612 | 0.408 | 391/391 |
| Gemini B | youden | 0.785000000000 | 0.641 | 0.734 | 0.375 | 389/391 |
| Gemini E1 | youden | 0.450000000000 | 0.797 | 0.722 | 0.519 | 389/391 |
| Gemini E2 | youden | 0.500000000000 | 0.719 | 0.801 | 0.520 | 391/391 |

**Panel B. Test and Holdout performance at 0.5 and the locked Development-derived threshold**

| **Condition** | **Cohort** | **Strategy** | **Threshold** | **Macro-F1** | **Sensitivity** | **Specificity** | **AUROC** | **Brier** | **Valid n/N** |
| --- | --- | --- | --- | --- | --- | --- | --- | --- | --- |
| LR | Holdout | Development-derived | 0.466260 | 0.696 | 0.810 | 0.682 | 0.832 | 0.179 | 2258/2258 |
| XGBoost | Holdout | Development-derived | 0.173913 | 0.652 | 0.878 | 0.579 | 0.835 | 0.147 | 2258/2258 |
| GPT E1 | Holdout | Development-derived | 0.380000 | 0.709 | 0.748 | 0.736 | 0.808 | 0.160 | 2258/2258 |
| Gemini E2 | Holdout | Development-derived | 0.500000 | 0.739 | 0.723 | 0.796 | 0.820 | 0.167 | 2256/2258 |
| LR | Holdout | Fixed 0.5 | 0.500000 | 0.712 | 0.784 | 0.720 | 0.832 | 0.179 | 2258/2258 |
| XGBoost | Holdout | Fixed 0.5 | 0.500000 | 0.632 | 0.266 | 0.976 | 0.835 | 0.147 | 2258/2258 |
| GPT E1 | Holdout | Fixed 0.5 | 0.500000 | 0.729 | 0.655 | 0.821 | 0.808 | 0.160 | 2258/2258 |
| Gemini E2 | Holdout | Fixed 0.5 | 0.500000 | 0.739 | 0.723 | 0.796 | 0.820 | 0.167 | 2256/2258 |
| LR | Test | Development-derived | 0.466260 | 0.695 | 0.801 | 0.707 | 0.844 | 0.174 | 2186/2186 |
| XGBoost | Test | Development-derived | 0.173913 | 0.660 | 0.871 | 0.622 | 0.846 | 0.129 | 2186/2186 |
| GPT A | Test | Development-derived | 0.248000 | 0.661 | 0.788 | 0.662 | 0.798 | 0.168 | 2185/2186 |
| GPT B | Test | Development-derived | 0.371000 | 0.678 | 0.777 | 0.694 | 0.789 | 0.176 | 2186/2186 |
| GPT E1 | Test | Development-derived | 0.380000 | 0.702 | 0.762 | 0.737 | 0.818 | 0.157 | 2186/2186 |
| GPT E2 | Test | Development-derived | 0.300000 | 0.681 | 0.845 | 0.665 | 0.826 | 0.153 | 2186/2186 |
| Claude A | Test | Development-derived | 0.250000 | 0.636 | 0.333 | 0.912 | 0.624 | 0.173 | 2186/2186 |
| Claude B | Test | Development-derived | 0.350000 | 0.650 | 0.818 | 0.631 | 0.783 | 0.163 | 2186/2186 |
| Claude E1 | Test | Development-derived | 0.500000 | 0.714 | 0.781 | 0.745 | 0.822 | 0.178 | 2186/2186 |
| Claude E2 | Test | Development-derived | 0.500000 | 0.713 | 0.738 | 0.765 | 0.816 | 0.175 | 2186/2186 |
| Gemini A | Test | Development-derived | 0.785000 | 0.664 | 0.807 | 0.658 | 0.814 | 0.294 | 2159/2186 |
| Gemini B | Test | Development-derived | 0.785000 | 0.677 | 0.727 | 0.717 | 0.818 | 0.286 | 2046/2186 |
| Gemini E1 | Test | Development-derived | 0.450000 | 0.674 | 0.816 | 0.668 | 0.839 | 0.195 | 2137/2186 |
| Gemini E2 | Test | Development-derived | 0.500000 | 0.730 | 0.742 | 0.788 | 0.834 | 0.167 | 2182/2186 |
| LR | Test | Fixed 0.5 | 0.500000 | 0.710 | 0.785 | 0.737 | 0.844 | 0.174 | 2186/2186 |
| XGBoost | Test | Fixed 0.5 | 0.500000 | 0.674 | 0.316 | 0.979 | 0.846 | 0.129 | 2186/2186 |
| GPT A | Test | Fixed 0.5 | 0.500000 | 0.706 | 0.631 | 0.813 | 0.798 | 0.168 | 2185/2186 |
| GPT B | Test | Fixed 0.5 | 0.500000 | 0.686 | 0.725 | 0.732 | 0.789 | 0.176 | 2186/2186 |
| GPT E1 | Test | Fixed 0.5 | 0.500000 | 0.732 | 0.691 | 0.817 | 0.818 | 0.157 | 2186/2186 |
| GPT E2 | Test | Fixed 0.5 | 0.500000 | 0.729 | 0.680 | 0.818 | 0.826 | 0.153 | 2186/2186 |
| Claude A | Test | Fixed 0.5 | 0.500000 | 0.606 | 0.242 | 0.950 | 0.624 | 0.173 | 2186/2186 |
| Claude B | Test | Fixed 0.5 | 0.500000 | 0.718 | 0.449 | 0.939 | 0.783 | 0.163 | 2186/2186 |
| Claude E1 | Test | Fixed 0.5 | 0.500000 | 0.714 | 0.781 | 0.745 | 0.822 | 0.178 | 2186/2186 |
| Claude E2 | Test | Fixed 0.5 | 0.500000 | 0.713 | 0.738 | 0.765 | 0.816 | 0.175 | 2186/2186 |
| Gemini A | Test | Fixed 0.5 | 0.500000 | 0.555 | 0.897 | 0.453 | 0.814 | 0.294 | 2159/2186 |
| Gemini B | Test | Fixed 0.5 | 0.500000 | 0.514 | 0.878 | 0.402 | 0.818 | 0.286 | 2046/2186 |
| Gemini E1 | Test | Fixed 0.5 | 0.500000 | 0.679 | 0.800 | 0.684 | 0.839 | 0.195 | 2137/2186 |
| Gemini E2 | Test | Fixed 0.5 | 0.500000 | 0.730 | 0.742 | 0.788 | 0.834 | 0.167 | 2182/2186 |

**Panel C. Operating points selected in Development-validation for sensitivity or specificity >=0.80**

| **Condition** | **Constraint** | **Target** | **Threshold** | **Macro-F1** | **Sensitivity** | **Specificity** | **Valid n/N** |
| --- | --- | --- | --- | --- | --- | --- | --- |
| LR | sensitivity | 0.80 | 0.466260 | 0.684 | 0.813 | 0.752 | 391/391 |
| LR | specificity | 0.80 | 0.533270 | 0.704 | 0.750 | 0.801 | 391/391 |
| XGBoost | sensitivity | 0.80 | 0.173913 | 0.605 | 0.844 | 0.630 | 391/391 |
| XGBoost | specificity | 0.80 | 0.250000 | 0.677 | 0.656 | 0.804 | 391/391 |
| GPT A | sensitivity | 0.80 | 0.218000 | 0.542 | 0.828 | 0.541 | 391/391 |
| GPT A | specificity | 0.80 | 0.338000 | 0.651 | 0.547 | 0.820 | 391/391 |
| GPT B | sensitivity | 0.80 | 0.312000 | 0.551 | 0.813 | 0.560 | 391/391 |
| GPT B | specificity | 0.80 | 0.596000 | 0.634 | 0.531 | 0.804 | 391/391 |
| GPT E1 | sensitivity | 0.80 | 0.340000 | 0.608 | 0.813 | 0.645 | 391/391 |
| GPT E1 | specificity | 0.80 | 0.440000 | 0.697 | 0.703 | 0.810 | 391/391 |
| GPT E2 | sensitivity | 0.80 | 0.300000 | 0.622 | 0.828 | 0.661 | 391/391 |
| GPT E2 | specificity | 0.80 | 0.400000 | 0.684 | 0.656 | 0.813 | 391/391 |
| Claude A | sensitivity | 0.80 | 0.150000 | 0.160 | 0.969 | 0.021 | 391/391 |
| Claude A | specificity | 0.80 | 0.250000 | 0.591 | 0.266 | 0.902 | 391/391 |
| Claude B | sensitivity | 0.80 | 0.320000 | 0.425 | 0.922 | 0.339 | 391/391 |
| Claude B | specificity | 0.80 | 0.720000 | 0.629 | 0.266 | 0.951 | 391/391 |
| Claude E1 | sensitivity | 0.80 | 0.350000 | 0.473 | 0.906 | 0.413 | 391/391 |
| Claude E1 | specificity | 0.80 | 0.680000 | 0.682 | 0.672 | 0.804 | 391/391 |
| Claude E2 | sensitivity | 0.80 | 0.350000 | 0.543 | 0.859 | 0.532 | 391/391 |
| Claude E2 | specificity | 0.80 | 0.720000 | 0.671 | 0.625 | 0.810 | 391/391 |
| Gemini A | sensitivity | 0.80 | 0.725000 | 0.532 | 0.828 | 0.526 | 391/391 |
| Gemini A | specificity | 0.80 | 0.825000 | 0.657 | 0.469 | 0.865 | 391/391 |
| Gemini B | sensitivity | 0.80 | 0.685000 | 0.493 | 0.891 | 0.446 | 389/391 |
| Gemini B | specificity | 0.80 | 0.825000 | 0.648 | 0.375 | 0.905 | 389/391 |
| Gemini E1 | sensitivity | 0.80 | 0.420000 | 0.604 | 0.813 | 0.639 | 389/391 |
| Gemini E1 | specificity | 0.80 | 0.680000 | 0.687 | 0.688 | 0.804 | 389/391 |
| Gemini E2 | sensitivity | 0.80 | 0.350000 | 0.555 | 0.891 | 0.538 | 391/391 |
| Gemini E2 | specificity | 0.80 | 0.650000 | 0.694 | 0.719 | 0.801 | 391/391 |

**Panel D. Transport of Development-selected fixed operating points to evaluation cohorts**

| **Condition** | **Cohort** | **Development constraint** | **Target** | **Threshold** | **Macro-F1** | **Sensitivity** | **Specificity** | **AUROC** | **Brier** |
| --- | --- | --- | --- | --- | --- | --- | --- | --- | --- |
| LR | Holdout | sensitivity | 0.80 | 0.466260 | 0.696 | 0.810 | 0.682 | 0.832 | 0.179 |
| LR | Holdout | specificity | 0.80 | 0.533270 | 0.723 | 0.759 | 0.750 | 0.832 | 0.179 |
| XGBoost | Holdout | sensitivity | 0.80 | 0.173913 | 0.652 | 0.878 | 0.579 | 0.835 | 0.147 |
| XGBoost | Holdout | specificity | 0.80 | 0.250000 | 0.733 | 0.764 | 0.763 | 0.835 | 0.147 |
| GPT E1 | Holdout | sensitivity | 0.80 | 0.340000 | 0.682 | 0.808 | 0.660 | 0.808 | 0.160 |
| GPT E1 | Holdout | specificity | 0.80 | 0.440000 | 0.730 | 0.681 | 0.806 | 0.808 | 0.160 |
| Gemini E2 | Holdout | sensitivity | 0.80 | 0.350000 | 0.628 | 0.872 | 0.545 | 0.820 | 0.167 |
| Gemini E2 | Holdout | specificity | 0.80 | 0.650000 | 0.739 | 0.723 | 0.796 | 0.820 | 0.167 |
| LR | Test | sensitivity | 0.80 | 0.466260 | 0.695 | 0.801 | 0.707 | 0.844 | 0.174 |
| LR | Test | specificity | 0.80 | 0.533270 | 0.724 | 0.762 | 0.768 | 0.844 | 0.174 |
| XGBoost | Test | sensitivity | 0.80 | 0.173913 | 0.660 | 0.871 | 0.622 | 0.846 | 0.129 |
| XGBoost | Test | specificity | 0.80 | 0.250000 | 0.738 | 0.757 | 0.791 | 0.846 | 0.129 |
| GPT A | Test | sensitivity | 0.80 | 0.218000 | 0.594 | 0.876 | 0.520 | 0.798 | 0.168 |
| GPT A | Test | specificity | 0.80 | 0.338000 | 0.710 | 0.661 | 0.801 | 0.798 | 0.168 |
| GPT B | Test | sensitivity | 0.80 | 0.312000 | 0.636 | 0.833 | 0.602 | 0.789 | 0.176 |
| GPT B | Test | specificity | 0.80 | 0.596000 | 0.689 | 0.697 | 0.752 | 0.789 | 0.176 |
| GPT E1 | Test | sensitivity | 0.80 | 0.340000 | 0.661 | 0.816 | 0.648 | 0.818 | 0.157 |
| GPT E1 | Test | specificity | 0.80 | 0.440000 | 0.726 | 0.715 | 0.795 | 0.818 | 0.157 |
| GPT E2 | Test | sensitivity | 0.80 | 0.300000 | 0.681 | 0.845 | 0.665 | 0.826 | 0.153 |
| GPT E2 | Test | specificity | 0.80 | 0.400000 | 0.718 | 0.721 | 0.781 | 0.826 | 0.153 |
| Claude A | Test | sensitivity | 0.80 | 0.150000 | 0.206 | 0.989 | 0.010 | 0.624 | 0.173 |
| Claude A | Test | specificity | 0.80 | 0.250000 | 0.636 | 0.333 | 0.912 | 0.624 | 0.173 |
| Claude B | Test | sensitivity | 0.80 | 0.320000 | 0.490 | 0.901 | 0.360 | 0.783 | 0.163 |
| Claude B | Test | specificity | 0.80 | 0.720000 | 0.718 | 0.449 | 0.939 | 0.783 | 0.163 |
| Claude E1 | Test | sensitivity | 0.80 | 0.350000 | 0.554 | 0.903 | 0.449 | 0.822 | 0.178 |
| Claude E1 | Test | specificity | 0.80 | 0.680000 | 0.734 | 0.738 | 0.795 | 0.822 | 0.178 |
| Claude E2 | Test | sensitivity | 0.80 | 0.350000 | 0.612 | 0.863 | 0.553 | 0.816 | 0.175 |
| Claude E2 | Test | specificity | 0.80 | 0.720000 | 0.727 | 0.704 | 0.802 | 0.816 | 0.175 |
| Gemini A | Test | sensitivity | 0.80 | 0.725000 | 0.601 | 0.867 | 0.534 | 0.814 | 0.294 |
| Gemini A | Test | specificity | 0.80 | 0.825000 | 0.728 | 0.631 | 0.844 | 0.814 | 0.294 |
| Gemini B | Test | sensitivity | 0.80 | 0.685000 | 0.535 | 0.865 | 0.437 | 0.818 | 0.286 |
| Gemini B | Test | specificity | 0.80 | 0.825000 | 0.688 | 0.577 | 0.818 | 0.818 | 0.286 |
| Gemini E1 | Test | sensitivity | 0.80 | 0.420000 | 0.656 | 0.839 | 0.630 | 0.839 | 0.195 |
| Gemini E1 | Test | specificity | 0.80 | 0.680000 | 0.704 | 0.719 | 0.762 | 0.839 | 0.195 |
| Gemini E2 | Test | sensitivity | 0.80 | 0.350000 | 0.610 | 0.891 | 0.537 | 0.834 | 0.167 |
| Gemini E2 | Test | specificity | 0.80 | 0.650000 | 0.730 | 0.742 | 0.788 | 0.834 | 0.167 |

***Note.*** *All thresholds were selected without Test or Holdout labels and then applied unchanged. The threshold comparison used the prespecified >= rule with 12-decimal comparison precision. Hard-label metrics include failure penalties for invalid LLM outputs; AUROC and Brier use valid probabilities and report their denominators.*

**Table S7.** Paired bootstrap differences between models and bootstrap ranking stability of the 12 LLM conditions.

**Panel A. Paired model differences (A minus B), 1,000 bootstrap resamples**

| **Cohort** | **Model A** | **Model B** | **Strategy** | **Metric** | **A** | **B** | **Difference (95% CI)** | **Paired n** |
| --- | --- | --- | --- | --- | --- | --- | --- | --- |
| Holdout | Gemini E2 | LR | Fixed 0.5 | Macro-F1 | 0.739 | 0.712 | 0.027 (0.013 to 0.041) | 2258 |
| Holdout | Gemini E2 | LR | Fixed 0.5 | Sensitivity | 0.723 | 0.784 | -0.062 (-0.086 to -0.040) | 2258 |
| Holdout | Gemini E2 | LR | Fixed 0.5 | Specificity | 0.796 | 0.720 | 0.076 (0.060 to 0.092) | 2258 |
| Holdout | Gemini E2 | LR | Fixed 0.5 | AUROC | 0.820 | 0.832 | -0.013 (-0.021 to -0.003) | 2256 |
| Holdout | Gemini E2 | LR | Fixed 0.5 | Brier score | 0.167 | 0.179 | -0.012 (-0.017 to -0.008) | 2256 |
| Holdout | Gemini E2 | LR | Development-derived | Macro-F1 | 0.739 | 0.696 | 0.043 (0.028 to 0.058) | 2258 |
| Holdout | Gemini E2 | LR | Development-derived | Sensitivity | 0.723 | 0.810 | -0.087 (-0.113 to -0.063) | 2258 |
| Holdout | Gemini E2 | LR | Development-derived | Specificity | 0.796 | 0.682 | 0.114 (0.097 to 0.133) | 2258 |
| Holdout | Gemini E2 | LR | Development-derived | AUROC | 0.820 | 0.832 | -0.013 (-0.021 to -0.003) | 2256 |
| Holdout | Gemini E2 | LR | Development-derived | Brier score | 0.167 | 0.179 | -0.012 (-0.017 to -0.008) | 2256 |
| Holdout | Gemini E2 | XGBoost | Fixed 0.5 | Macro-F1 | 0.739 | 0.632 | 0.107 (0.077 to 0.134) | 2258 |
| Holdout | Gemini E2 | XGBoost | Fixed 0.5 | Sensitivity | 0.723 | 0.266 | 0.456 (0.415 to 0.497) | 2258 |
| Holdout | Gemini E2 | XGBoost | Fixed 0.5 | Specificity | 0.796 | 0.976 | -0.180 (-0.200 to -0.162) | 2258 |
| Holdout | Gemini E2 | XGBoost | Fixed 0.5 | AUROC | 0.820 | 0.835 | -0.015 (-0.030 to -0.001) | 2256 |
| Holdout | Gemini E2 | XGBoost | Fixed 0.5 | Brier score | 0.167 | 0.147 | 0.020 (0.011 to 0.029) | 2256 |
| Holdout | Gemini E2 | XGBoost | Development-derived | Macro-F1 | 0.739 | 0.652 | 0.087 (0.069 to 0.105) | 2258 |
| Holdout | Gemini E2 | XGBoost | Development-derived | Sensitivity | 0.723 | 0.878 | -0.155 (-0.185 to -0.126) | 2258 |
| Holdout | Gemini E2 | XGBoost | Development-derived | Specificity | 0.796 | 0.579 | 0.217 (0.196 to 0.239) | 2258 |
| Holdout | Gemini E2 | XGBoost | Development-derived | AUROC | 0.820 | 0.835 | -0.015 (-0.030 to -0.001) | 2256 |
| Holdout | Gemini E2 | XGBoost | Development-derived | Brier score | 0.167 | 0.147 | 0.020 (0.011 to 0.029) | 2256 |
| Holdout | GPT E1 | Gemini E2 | Fixed 0.5 | Macro-F1 | 0.729 | 0.739 | -0.011 (-0.025 to 0.006) | 2258 |
| Holdout | GPT E1 | Gemini E2 | Fixed 0.5 | Sensitivity | 0.655 | 0.723 | -0.068 (-0.095 to -0.040) | 2258 |
| Holdout | GPT E1 | Gemini E2 | Fixed 0.5 | Specificity | 0.821 | 0.796 | 0.025 (0.010 to 0.039) | 2258 |
| Holdout | GPT E1 | Gemini E2 | Fixed 0.5 | AUROC | 0.808 | 0.820 | -0.011 (-0.019 to -0.003) | 2256 |
| Holdout | GPT E1 | Gemini E2 | Fixed 0.5 | Brier score | 0.160 | 0.167 | -0.007 (-0.011 to -0.003) | 2256 |
| Holdout | GPT E1 | Gemini E2 | Development-derived | Macro-F1 | 0.709 | 0.739 | -0.030 (-0.043 to -0.018) | 2258 |
| Holdout | GPT E1 | Gemini E2 | Development-derived | Sensitivity | 0.748 | 0.723 | 0.025 (0.003 to 0.047) | 2258 |
| Holdout | GPT E1 | Gemini E2 | Development-derived | Specificity | 0.736 | 0.796 | -0.060 (-0.075 to -0.046) | 2258 |
| Holdout | GPT E1 | Gemini E2 | Development-derived | AUROC | 0.808 | 0.820 | -0.011 (-0.019 to -0.003) | 2256 |
| Holdout | GPT E1 | Gemini E2 | Development-derived | Brier score | 0.160 | 0.167 | -0.007 (-0.011 to -0.003) | 2256 |
| Holdout | GPT E1 | LR | Fixed 0.5 | Macro-F1 | 0.729 | 0.712 | 0.017 (-0.002 to 0.034) | 2258 |
| Holdout | GPT E1 | LR | Fixed 0.5 | Sensitivity | 0.655 | 0.784 | -0.130 (-0.161 to -0.097) | 2258 |
| Holdout | GPT E1 | LR | Fixed 0.5 | Specificity | 0.821 | 0.720 | 0.101 (0.082 to 0.120) | 2258 |
| Holdout | GPT E1 | LR | Fixed 0.5 | AUROC | 0.808 | 0.832 | -0.024 (-0.035 to -0.013) | 2258 |
| Holdout | GPT E1 | LR | Fixed 0.5 | Brier score | 0.160 | 0.179 | -0.019 (-0.025 to -0.013) | 2258 |
| Holdout | GPT E1 | LR | Development-derived | Macro-F1 | 0.709 | 0.696 | 0.013 (-0.002 to 0.028) | 2258 |
| Holdout | GPT E1 | LR | Development-derived | Sensitivity | 0.748 | 0.810 | -0.062 (-0.088 to -0.037) | 2258 |
| Holdout | GPT E1 | LR | Development-derived | Specificity | 0.736 | 0.682 | 0.054 (0.036 to 0.073) | 2258 |
| Holdout | GPT E1 | LR | Development-derived | AUROC | 0.808 | 0.832 | -0.024 (-0.035 to -0.013) | 2258 |
| Holdout | GPT E1 | LR | Development-derived | Brier score | 0.160 | 0.179 | -0.019 (-0.025 to -0.013) | 2258 |
| Holdout | GPT E1 | XGBoost | Fixed 0.5 | Macro-F1 | 0.729 | 0.632 | 0.097 (0.071 to 0.122) | 2258 |
| Holdout | GPT E1 | XGBoost | Fixed 0.5 | Sensitivity | 0.655 | 0.266 | 0.388 (0.352 to 0.426) | 2258 |
| Holdout | GPT E1 | XGBoost | Fixed 0.5 | Specificity | 0.821 | 0.976 | -0.156 (-0.174 to -0.138) | 2258 |
| Holdout | GPT E1 | XGBoost | Fixed 0.5 | AUROC | 0.808 | 0.835 | -0.027 (-0.042 to -0.010) | 2258 |
| Holdout | GPT E1 | XGBoost | Fixed 0.5 | Brier score | 0.160 | 0.147 | 0.013 (0.005 to 0.021) | 2258 |
| Holdout | GPT E1 | XGBoost | Development-derived | Macro-F1 | 0.709 | 0.652 | 0.057 (0.039 to 0.075) | 2258 |
| Holdout | GPT E1 | XGBoost | Development-derived | Sensitivity | 0.748 | 0.878 | -0.130 (-0.160 to -0.102) | 2258 |
| Holdout | GPT E1 | XGBoost | Development-derived | Specificity | 0.736 | 0.579 | 0.157 (0.135 to 0.179) | 2258 |
| Holdout | GPT E1 | XGBoost | Development-derived | AUROC | 0.808 | 0.835 | -0.027 (-0.042 to -0.010) | 2258 |
| Holdout | GPT E1 | XGBoost | Development-derived | Brier score | 0.160 | 0.147 | 0.013 (0.005 to 0.021) | 2258 |
| Test | Gemini E2 | LR | Fixed 0.5 | Macro-F1 | 0.730 | 0.710 | 0.020 (0.006 to 0.035) | 2186 |
| Test | Gemini E2 | LR | Fixed 0.5 | Sensitivity | 0.742 | 0.785 | -0.043 (-0.069 to -0.016) | 2186 |
| Test | Gemini E2 | LR | Fixed 0.5 | Specificity | 0.788 | 0.737 | 0.051 (0.035 to 0.067) | 2186 |
| Test | Gemini E2 | LR | Fixed 0.5 | AUROC | 0.834 | 0.844 | -0.010 (-0.020 to 0.000) | 2182 |
| Test | Gemini E2 | LR | Fixed 0.5 | Brier score | 0.167 | 0.174 | -0.007 (-0.012 to -0.002) | 2182 |
| Test | Gemini E2 | LR | Development-derived | Macro-F1 | 0.730 | 0.695 | 0.035 (0.020 to 0.049) | 2186 |
| Test | Gemini E2 | LR | Development-derived | Sensitivity | 0.742 | 0.801 | -0.060 (-0.087 to -0.031) | 2186 |
| Test | Gemini E2 | LR | Development-derived | Specificity | 0.788 | 0.707 | 0.081 (0.063 to 0.096) | 2186 |
| Test | Gemini E2 | LR | Development-derived | AUROC | 0.834 | 0.844 | -0.010 (-0.020 to 0.000) | 2182 |
| Test | Gemini E2 | LR | Development-derived | Brier score | 0.167 | 0.174 | -0.007 (-0.012 to -0.002) | 2182 |
| Test | Gemini E2 | GPT E2 | Fixed 0.5 | Macro-F1 | 0.730 | 0.729 | 0.001 (-0.012 to 0.014) | 2186 |
| Test | Gemini E2 | GPT E2 | Fixed 0.5 | Sensitivity | 0.742 | 0.680 | 0.062 (0.037 to 0.086) | 2186 |
| Test | Gemini E2 | GPT E2 | Fixed 0.5 | Specificity | 0.788 | 0.818 | -0.031 (-0.043 to -0.019) | 2186 |
| Test | Gemini E2 | GPT E2 | Fixed 0.5 | AUROC | 0.834 | 0.826 | 0.007 (0.001 to 0.014) | 2182 |
| Test | Gemini E2 | GPT E2 | Fixed 0.5 | Brier score | 0.167 | 0.153 | 0.014 (0.010 to 0.018) | 2182 |
| Test | Gemini E2 | GPT E2 | Development-derived | Macro-F1 | 0.730 | 0.681 | 0.049 (0.034 to 0.063) | 2186 |
| Test | Gemini E2 | GPT E2 | Development-derived | Sensitivity | 0.742 | 0.845 | -0.103 (-0.130 to -0.078) | 2186 |
| Test | Gemini E2 | GPT E2 | Development-derived | Specificity | 0.788 | 0.665 | 0.122 (0.107 to 0.138) | 2186 |
| Test | Gemini E2 | GPT E2 | Development-derived | AUROC | 0.834 | 0.826 | 0.007 (0.001 to 0.014) | 2182 |
| Test | Gemini E2 | GPT E2 | Development-derived | Brier score | 0.167 | 0.153 | 0.014 (0.010 to 0.018) | 2182 |
| Test | Gemini E2 | XGBoost | Fixed 0.5 | Macro-F1 | 0.730 | 0.674 | 0.056 (0.028 to 0.083) | 2186 |
| Test | Gemini E2 | XGBoost | Fixed 0.5 | Sensitivity | 0.742 | 0.316 | 0.425 (0.384 to 0.466) | 2186 |
| Test | Gemini E2 | XGBoost | Fixed 0.5 | Specificity | 0.788 | 0.979 | -0.192 (-0.212 to -0.172) | 2186 |
| Test | Gemini E2 | XGBoost | Fixed 0.5 | AUROC | 0.834 | 0.846 | -0.013 (-0.028 to 0.003) | 2182 |
| Test | Gemini E2 | XGBoost | Fixed 0.5 | Brier score | 0.167 | 0.128 | 0.038 (0.029 to 0.047) | 2182 |
| Test | Gemini E2 | XGBoost | Development-derived | Macro-F1 | 0.730 | 0.660 | 0.070 (0.052 to 0.086) | 2186 |
| Test | Gemini E2 | XGBoost | Development-derived | Sensitivity | 0.742 | 0.871 | -0.129 (-0.161 to -0.098) | 2186 |
| Test | Gemini E2 | XGBoost | Development-derived | Specificity | 0.788 | 0.622 | 0.166 (0.146 to 0.185) | 2186 |
| Test | Gemini E2 | XGBoost | Development-derived | AUROC | 0.834 | 0.846 | -0.013 (-0.028 to 0.003) | 2182 |
| Test | Gemini E2 | XGBoost | Development-derived | Brier score | 0.167 | 0.128 | 0.038 (0.029 to 0.047) | 2182 |
| Test | GPT E1 | Gemini E2 | Fixed 0.5 | Macro-F1 | 0.732 | 0.730 | 0.002 (-0.013 to 0.016) | 2186 |
| Test | GPT E1 | Gemini E2 | Fixed 0.5 | Sensitivity | 0.691 | 0.742 | -0.051 (-0.079 to -0.024) | 2186 |
| Test | GPT E1 | Gemini E2 | Fixed 0.5 | Specificity | 0.817 | 0.788 | 0.030 (0.015 to 0.044) | 2186 |
| Test | GPT E1 | Gemini E2 | Fixed 0.5 | AUROC | 0.818 | 0.834 | -0.016 (-0.023 to -0.008) | 2182 |
| Test | GPT E1 | Gemini E2 | Fixed 0.5 | Brier score | 0.156 | 0.167 | -0.010 (-0.015 to -0.006) | 2182 |
| Test | GPT E1 | Gemini E2 | Development-derived | Macro-F1 | 0.702 | 0.730 | -0.028 (-0.040 to -0.016) | 2186 |
| Test | GPT E1 | Gemini E2 | Development-derived | Sensitivity | 0.762 | 0.742 | 0.021 (-0.004 to 0.043) | 2186 |
| Test | GPT E1 | Gemini E2 | Development-derived | Specificity | 0.737 | 0.788 | -0.050 (-0.064 to -0.037) | 2186 |
| Test | GPT E1 | Gemini E2 | Development-derived | AUROC | 0.818 | 0.834 | -0.016 (-0.023 to -0.008) | 2182 |
| Test | GPT E1 | Gemini E2 | Development-derived | Brier score | 0.156 | 0.167 | -0.010 (-0.015 to -0.006) | 2182 |
| Test | GPT E1 | LR | Fixed 0.5 | Macro-F1 | 0.732 | 0.710 | 0.023 (0.005 to 0.042) | 2186 |
| Test | GPT E1 | LR | Fixed 0.5 | Sensitivity | 0.691 | 0.785 | -0.094 (-0.125 to -0.062) | 2186 |
| Test | GPT E1 | LR | Fixed 0.5 | Specificity | 0.817 | 0.737 | 0.081 (0.061 to 0.102) | 2186 |
| Test | GPT E1 | LR | Fixed 0.5 | AUROC | 0.818 | 0.844 | -0.026 (-0.038 to -0.014) | 2186 |
| Test | GPT E1 | LR | Fixed 0.5 | Brier score | 0.157 | 0.174 | -0.017 (-0.023 to -0.011) | 2186 |
| Test | GPT E1 | LR | Development-derived | Macro-F1 | 0.702 | 0.695 | 0.007 (-0.009 to 0.024) | 2186 |
| Test | GPT E1 | LR | Development-derived | Sensitivity | 0.762 | 0.801 | -0.039 (-0.065 to -0.011) | 2186 |
| Test | GPT E1 | LR | Development-derived | Specificity | 0.737 | 0.707 | 0.030 (0.012 to 0.049) | 2186 |
| Test | GPT E1 | LR | Development-derived | AUROC | 0.818 | 0.844 | -0.026 (-0.038 to -0.014) | 2186 |
| Test | GPT E1 | LR | Development-derived | Brier score | 0.157 | 0.174 | -0.017 (-0.023 to -0.011) | 2186 |
| Test | GPT E1 | GPT E2 | Fixed 0.5 | Macro-F1 | 0.732 | 0.729 | 0.003 (-0.010 to 0.017) | 2186 |
| Test | GPT E1 | GPT E2 | Fixed 0.5 | Sensitivity | 0.691 | 0.680 | 0.011 (-0.014 to 0.039) | 2186 |
| Test | GPT E1 | GPT E2 | Fixed 0.5 | Specificity | 0.817 | 0.818 | -0.001 (-0.016 to 0.013) | 2186 |
| Test | GPT E1 | GPT E2 | Fixed 0.5 | AUROC | 0.818 | 0.826 | -0.009 (-0.016 to -0.003) | 2186 |
| Test | GPT E1 | GPT E2 | Fixed 0.5 | Brier score | 0.157 | 0.153 | 0.004 (0.000 to 0.008) | 2186 |
| Test | GPT E1 | GPT E2 | Development-derived | Macro-F1 | 0.702 | 0.681 | 0.021 (0.008 to 0.035) | 2186 |
| Test | GPT E1 | GPT E2 | Development-derived | Sensitivity | 0.762 | 0.845 | -0.082 (-0.105 to -0.060) | 2186 |
| Test | GPT E1 | GPT E2 | Development-derived | Specificity | 0.737 | 0.665 | 0.072 (0.057 to 0.087) | 2186 |
| Test | GPT E1 | GPT E2 | Development-derived | AUROC | 0.818 | 0.826 | -0.009 (-0.016 to -0.003) | 2186 |
| Test | GPT E1 | GPT E2 | Development-derived | Brier score | 0.157 | 0.153 | 0.004 (0.000 to 0.008) | 2186 |
| Test | GPT E1 | XGBoost | Fixed 0.5 | Macro-F1 | 0.732 | 0.674 | 0.058 (0.029 to 0.086) | 2186 |
| Test | GPT E1 | XGBoost | Fixed 0.5 | Sensitivity | 0.691 | 0.316 | 0.375 (0.331 to 0.418) | 2186 |
| Test | GPT E1 | XGBoost | Fixed 0.5 | Specificity | 0.817 | 0.979 | -0.162 (-0.182 to -0.145) | 2186 |
| Test | GPT E1 | XGBoost | Fixed 0.5 | AUROC | 0.818 | 0.846 | -0.029 (-0.045 to -0.012) | 2186 |
| Test | GPT E1 | XGBoost | Fixed 0.5 | Brier score | 0.157 | 0.129 | 0.028 (0.019 to 0.037) | 2186 |
| Test | GPT E1 | XGBoost | Development-derived | Macro-F1 | 0.702 | 0.660 | 0.042 (0.025 to 0.060) | 2186 |
| Test | GPT E1 | XGBoost | Development-derived | Sensitivity | 0.762 | 0.871 | -0.109 (-0.138 to -0.076) | 2186 |
| Test | GPT E1 | XGBoost | Development-derived | Specificity | 0.737 | 0.622 | 0.116 (0.095 to 0.135) | 2186 |
| Test | GPT E1 | XGBoost | Development-derived | AUROC | 0.818 | 0.846 | -0.029 (-0.045 to -0.012) | 2186 |
| Test | GPT E1 | XGBoost | Development-derived | Brier score | 0.157 | 0.129 | 0.028 (0.019 to 0.037) | 2186 |

**Panel B. Test bootstrap rank stability across all 12 LLM conditions**

| **Strategy** | **Condition** | **Ranked first** | **Top two** | **Median rank (IQR)** | **Bootstrap samples** |
| --- | --- | --- | --- | --- | --- |
| Development-derived | GPT A | 0.0% | 0.0% | 10.0 (9.0-10.0) | 1000 |
| Development-derived | GPT B | 0.0% | 0.0% | 6.0 (5.0-7.0) | 1000 |
| Development-derived | GPT E1 | 0.0% | 1.2% | 4.0 (4.0-4.0) | 1000 |
| Development-derived | GPT E2 | 0.0% | 0.0% | 6.0 (5.0-6.0) | 1000 |
| Development-derived | Claude A | 0.0% | 0.0% | 12.0 (12.0-12.0) | 1000 |
| Development-derived | Claude B | 0.0% | 0.0% | 11.0 (11.0-11.0) | 1000 |
| Development-derived | Claude E1 | 0.1% | 54.7% | 2.0 (2.0-3.0) | 1000 |
| Development-derived | Claude E2 | 0.0% | 44.1% | 3.0 (2.0-3.0) | 1000 |
| Development-derived | Gemini A | 0.0% | 0.0% | 9.0 (9.0-10.0) | 1000 |
| Development-derived | Gemini B | 0.0% | 0.0% | 7.0 (6.0-8.0) | 1000 |
| Development-derived | Gemini E1 | 0.0% | 0.0% | 8.0 (7.0-8.0) | 1000 |
| Development-derived | Gemini E2 | 99.9% | 100.0% | 1.0 (1.0-1.0) | 1000 |
| Fixed 0.5 | GPT A | 0.1% | 0.1% | 7.0 (6.0-7.0) | 1000 |
| Fixed 0.5 | GPT B | 0.0% | 0.0% | 8.0 (8.0-8.0) | 1000 |
| Fixed 0.5 | GPT E1 | 49.0% | 73.4% | 2.0 (1.0-3.0) | 1000 |
| Fixed 0.5 | GPT E2 | 17.6% | 52.6% | 2.0 (2.0-3.0) | 1000 |
| Fixed 0.5 | Claude A | 0.0% | 0.0% | 10.0 (10.0-10.0) | 1000 |
| Fixed 0.5 | Claude B | 7.3% | 12.3% | 4.0 (4.0-6.0) | 1000 |
| Fixed 0.5 | Claude E1 | 0.0% | 0.1% | 5.0 (5.0-6.0) | 1000 |
| Fixed 0.5 | Claude E2 | 0.0% | 0.0% | 5.0 (5.0-6.0) | 1000 |
| Fixed 0.5 | Gemini A | 0.0% | 0.0% | 11.0 (11.0-11.0) | 1000 |
| Fixed 0.5 | Gemini B | 0.0% | 0.0% | 12.0 (12.0-12.0) | 1000 |
| Fixed 0.5 | Gemini E1 | 0.0% | 0.0% | 9.0 (9.0-9.0) | 1000 |
| Fixed 0.5 | Gemini E2 | 26.0% | 61.5% | 2.0 (1.0-3.0) | 1000 |

***Note.*** *Each paired replicate used identical subject indices for both models. AUROC and Brier differences used the two-model common-valid intersection; hard-label metrics used the full cohort. Percentile 95% confidence intervals are descriptive and were not used for multiplicity-adjusted significance claims or finalist reselection.*

**Table S8.** Scale correlations, grouped machine-learning ablations, demographics-only benchmarking, and exploratory LLM input-ablation analyses.

**Panel A. Spearman correlations between continuous SDS score and questionnaire predictors**

| **Cohort** | **Predictor** | **Spearman rho** | **95% CI** | **n** |
| --- | --- | --- | --- | --- |
| Development | sas_std | 0.558 | 0.528 to 0.589 | 1951 |
| Development | cfq_total | 0.431 | 0.387 to 0.470 | 1951 |
| Development | aaq2_total | 0.451 | 0.415 to 0.488 | 1951 |
| Development | scl90_somatization | 0.430 | 0.394 to 0.466 | 1951 |
| Development | scl90_obsessive_compulsive | 0.398 | 0.361 to 0.436 | 1951 |
| Development | scl90_interpersonal_sensitivity | 0.454 | 0.415 to 0.490 | 1951 |
| Development | scl90_anxiety | 0.461 | 0.424 to 0.497 | 1951 |
| Development | scl90_hostility | 0.407 | 0.368 to 0.443 | 1951 |
| Development | scl90_phobic_anxiety | 0.401 | 0.362 to 0.440 | 1951 |
| Development | scl90_paranoid_ideation | 0.421 | 0.383 to 0.459 | 1951 |
| Development | scl90_psychoticism | 0.442 | 0.405 to 0.478 | 1951 |
| Development | scl90_additional_items | 0.430 | 0.391 to 0.470 | 1951 |
| Test | sas_std | 0.595 | 0.564 to 0.624 | 2186 |
| Test | cfq_total | 0.435 | 0.397 to 0.471 | 2186 |
| Test | aaq2_total | 0.469 | 0.432 to 0.503 | 2186 |
| Test | scl90_somatization | 0.533 | 0.500 to 0.565 | 2186 |
| Test | scl90_obsessive_compulsive | 0.454 | 0.419 to 0.491 | 2186 |
| Test | scl90_interpersonal_sensitivity | 0.497 | 0.459 to 0.534 | 2186 |
| Test | scl90_anxiety | 0.540 | 0.506 to 0.571 | 2186 |
| Test | scl90_hostility | 0.522 | 0.486 to 0.554 | 2186 |
| Test | scl90_phobic_anxiety | 0.487 | 0.453 to 0.521 | 2186 |
| Test | scl90_paranoid_ideation | 0.473 | 0.437 to 0.507 | 2186 |
| Test | scl90_psychoticism | 0.520 | 0.486 to 0.552 | 2186 |
| Test | scl90_additional_items | 0.523 | 0.490 to 0.555 | 2186 |
| Holdout | sas_std | 0.620 | 0.592 to 0.647 | 2258 |
| Holdout | cfq_total | 0.411 | 0.373 to 0.448 | 2258 |
| Holdout | aaq2_total | 0.470 | 0.434 to 0.502 | 2258 |
| Holdout | scl90_somatization | 0.544 | 0.511 to 0.575 | 2258 |
| Holdout | scl90_obsessive_compulsive | 0.438 | 0.399 to 0.474 | 2258 |
| Holdout | scl90_interpersonal_sensitivity | 0.499 | 0.462 to 0.531 | 2258 |
| Holdout | scl90_anxiety | 0.546 | 0.514 to 0.578 | 2258 |
| Holdout | scl90_hostility | 0.512 | 0.479 to 0.543 | 2258 |
| Holdout | scl90_phobic_anxiety | 0.483 | 0.452 to 0.517 | 2258 |
| Holdout | scl90_paranoid_ideation | 0.453 | 0.416 to 0.488 | 2258 |
| Holdout | scl90_psychoticism | 0.503 | 0.468 to 0.535 | 2258 |
| Holdout | scl90_additional_items | 0.493 | 0.456 to 0.525 | 2258 |
| Combined | sas_std | 0.597 | 0.581 to 0.615 | 6395 |
| Combined | cfq_total | 0.431 | 0.409 to 0.453 | 6395 |
| Combined | aaq2_total | 0.469 | 0.447 to 0.490 | 6395 |
| Combined | scl90_somatization | 0.509 | 0.489 to 0.530 | 6395 |
| Combined | scl90_obsessive_compulsive | 0.431 | 0.409 to 0.452 | 6395 |
| Combined | scl90_interpersonal_sensitivity | 0.486 | 0.465 to 0.506 | 6395 |
| Combined | scl90_anxiety | 0.521 | 0.499 to 0.541 | 6395 |
| Combined | scl90_hostility | 0.489 | 0.468 to 0.509 | 6395 |
| Combined | scl90_phobic_anxiety | 0.458 | 0.438 to 0.479 | 6395 |
| Combined | scl90_paranoid_ideation | 0.451 | 0.431 to 0.472 | 6395 |
| Combined | scl90_psychoticism | 0.492 | 0.472 to 0.512 | 6395 |
| Combined | scl90_additional_items | 0.490 | 0.469 to 0.510 | 6395 |

**Panel B. Full, leave-one-group-out, and single-group machine-learning results**

| **Model** | **Variant** | **Cohort** | **Strategy** | **Threshold** | **Macro-F1** | **AUROC** | **Brier** | **Valid n/N** |
| --- | --- | --- | --- | --- | --- | --- | --- | --- |
| LR | Full model/input | Holdout | Development-derived | 0.466260 | 0.696 | 0.832 | 0.179 | 2258/2258 |
| LR | Leave out AAQ-II | Holdout | Development-derived | 0.472378 | 0.704 | 0.827 | 0.181 | 2258/2258 |
| LR | Leave out CFQ | Holdout | Development-derived | 0.466223 | 0.696 | 0.832 | 0.179 | 2258/2258 |
| LR | Leave out SAS | Holdout | Development-derived | 0.668989 | 0.723 | 0.806 | 0.189 | 2258/2258 |
| LR | Leave out SCL-90 group | Holdout | Development-derived | 0.590296 | 0.736 | 0.826 | 0.182 | 2258/2258 |
| LR | AAQ-II + demographics | Holdout | Development-derived | 0.497841 | 0.631 | 0.727 | 0.215 | 2258/2258 |
| LR | CFQ + demographics | Holdout | Development-derived | 0.477235 | 0.590 | 0.694 | 0.221 | 2258/2258 |
| LR | SAS + demographics | Holdout | Development-derived | 0.463481 | 0.671 | 0.814 | 0.184 | 2258/2258 |
| LR | SCL-90 group + demographics | Holdout | Development-derived | 0.614412 | 0.727 | 0.789 | 0.191 | 2258/2258 |
| LR | Full model/input | Holdout | Fixed 0.5 | 0.500000 | 0.712 | 0.832 | 0.179 | 2258/2258 |
| LR | Leave out AAQ-II | Holdout | Fixed 0.5 | 0.500000 | 0.716 | 0.827 | 0.181 | 2258/2258 |
| LR | Leave out CFQ | Holdout | Fixed 0.5 | 0.500000 | 0.712 | 0.832 | 0.179 | 2258/2258 |
| LR | Leave out SAS | Holdout | Fixed 0.5 | 0.500000 | 0.703 | 0.806 | 0.189 | 2258/2258 |
| LR | Leave out SCL-90 group | Holdout | Fixed 0.5 | 0.500000 | 0.707 | 0.826 | 0.182 | 2258/2258 |
| LR | AAQ-II + demographics | Holdout | Fixed 0.5 | 0.500000 | 0.633 | 0.727 | 0.215 | 2258/2258 |
| LR | CFQ + demographics | Holdout | Fixed 0.5 | 0.500000 | 0.615 | 0.694 | 0.221 | 2258/2258 |
| LR | SAS + demographics | Holdout | Fixed 0.5 | 0.500000 | 0.706 | 0.814 | 0.184 | 2258/2258 |
| LR | SCL-90 group + demographics | Holdout | Fixed 0.5 | 0.500000 | 0.693 | 0.789 | 0.191 | 2258/2258 |
| XGBoost | Full model/input | Holdout | Development-derived | 0.173913 | 0.652 | 0.835 | 0.147 | 2258/2258 |
| XGBoost | Leave out AAQ-II | Holdout | Development-derived | 0.208333 | 0.687 | 0.832 | 0.146 | 2258/2258 |
| XGBoost | Leave out CFQ | Holdout | Development-derived | 0.204819 | 0.654 | 0.832 | 0.145 | 2258/2258 |
| XGBoost | Leave out SAS | Holdout | Development-derived | 0.272727 | 0.702 | 0.810 | 0.156 | 2258/2258 |
| XGBoost | Leave out SCL-90 group | Holdout | Development-derived | 0.210526 | 0.669 | 0.817 | 0.155 | 2258/2258 |
| XGBoost | AAQ-II + demographics | Holdout | Development-derived | 0.250000 | 0.639 | 0.698 | 0.189 | 2258/2258 |
| XGBoost | CFQ + demographics | Holdout | Development-derived | 0.182692 | 0.520 | 0.686 | 0.190 | 2258/2258 |
| XGBoost | SAS + demographics | Holdout | Development-derived | 0.234043 | 0.653 | 0.788 | 0.160 | 2258/2258 |
| XGBoost | SCL-90 group + demographics | Holdout | Development-derived | 0.164179 | 0.613 | 0.770 | 0.164 | 2258/2258 |
| XGBoost | Full model/input | Holdout | Fixed 0.5 | 0.500000 | 0.632 | 0.835 | 0.147 | 2258/2258 |
| XGBoost | Leave out AAQ-II | Holdout | Fixed 0.5 | 0.500000 | 0.652 | 0.832 | 0.146 | 2258/2258 |
| XGBoost | Leave out CFQ | Holdout | Fixed 0.5 | 0.500000 | 0.636 | 0.832 | 0.145 | 2258/2258 |
| XGBoost | Leave out SAS | Holdout | Fixed 0.5 | 0.500000 | 0.572 | 0.810 | 0.156 | 2258/2258 |
| XGBoost | Leave out SCL-90 group | Holdout | Fixed 0.5 | 0.500000 | 0.584 | 0.817 | 0.155 | 2258/2258 |
| XGBoost | AAQ-II + demographics | Holdout | Fixed 0.5 | 0.500000 | 0.437 | 0.698 | 0.189 | 2258/2258 |
| XGBoost | CFQ + demographics | Holdout | Fixed 0.5 | 0.500000 | 0.433 | 0.686 | 0.190 | 2258/2258 |
| XGBoost | SAS + demographics | Holdout | Fixed 0.5 | 0.500000 | 0.663 | 0.788 | 0.160 | 2258/2258 |
| XGBoost | SCL-90 group + demographics | Holdout | Fixed 0.5 | 0.500000 | 0.697 | 0.770 | 0.164 | 2258/2258 |
| LR | Full model/input | Test | Development-derived | 0.466260 | 0.695 | 0.844 | 0.174 | 2186/2186 |
| LR | Leave out AAQ-II | Test | Development-derived | 0.472378 | 0.697 | 0.840 | 0.174 | 2186/2186 |
| LR | Leave out CFQ | Test | Development-derived | 0.466223 | 0.695 | 0.844 | 0.174 | 2186/2186 |
| LR | Leave out SAS | Test | Development-derived | 0.668989 | 0.741 | 0.815 | 0.186 | 2186/2186 |
| LR | Leave out SCL-90 group | Test | Development-derived | 0.590296 | 0.729 | 0.831 | 0.182 | 2186/2186 |
| LR | AAQ-II + demographics | Test | Development-derived | 0.497841 | 0.616 | 0.738 | 0.217 | 2186/2186 |
| LR | CFQ + demographics | Test | Development-derived | 0.477235 | 0.578 | 0.714 | 0.222 | 2186/2186 |
| LR | SAS + demographics | Test | Development-derived | 0.463481 | 0.666 | 0.813 | 0.186 | 2186/2186 |
| LR | SCL-90 group + demographics | Test | Development-derived | 0.614412 | 0.732 | 0.798 | 0.188 | 2186/2186 |
| LR | Full model/input | Test | Fixed 0.5 | 0.500000 | 0.710 | 0.844 | 0.174 | 2186/2186 |
| LR | Leave out AAQ-II | Test | Fixed 0.5 | 0.500000 | 0.708 | 0.840 | 0.174 | 2186/2186 |
| LR | Leave out CFQ | Test | Fixed 0.5 | 0.500000 | 0.710 | 0.844 | 0.174 | 2186/2186 |
| LR | Leave out SAS | Test | Fixed 0.5 | 0.500000 | 0.687 | 0.815 | 0.186 | 2186/2186 |
| LR | Leave out SCL-90 group | Test | Fixed 0.5 | 0.500000 | 0.692 | 0.831 | 0.182 | 2186/2186 |
| LR | AAQ-II + demographics | Test | Fixed 0.5 | 0.500000 | 0.619 | 0.738 | 0.217 | 2186/2186 |
| LR | CFQ + demographics | Test | Fixed 0.5 | 0.500000 | 0.611 | 0.714 | 0.222 | 2186/2186 |
| LR | SAS + demographics | Test | Fixed 0.5 | 0.500000 | 0.694 | 0.813 | 0.186 | 2186/2186 |
| LR | SCL-90 group + demographics | Test | Fixed 0.5 | 0.500000 | 0.686 | 0.798 | 0.188 | 2186/2186 |
| XGBoost | Full model/input | Test | Development-derived | 0.173913 | 0.660 | 0.846 | 0.129 | 2186/2186 |
| XGBoost | Leave out AAQ-II | Test | Development-derived | 0.208333 | 0.692 | 0.845 | 0.128 | 2186/2186 |
| XGBoost | Leave out CFQ | Test | Development-derived | 0.204819 | 0.660 | 0.841 | 0.129 | 2186/2186 |
| XGBoost | Leave out SAS | Test | Development-derived | 0.272727 | 0.714 | 0.821 | 0.137 | 2186/2186 |
| XGBoost | Leave out SCL-90 group | Test | Development-derived | 0.210526 | 0.675 | 0.828 | 0.139 | 2186/2186 |
| XGBoost | AAQ-II + demographics | Test | Development-derived | 0.250000 | 0.645 | 0.716 | 0.168 | 2186/2186 |
| XGBoost | CFQ + demographics | Test | Development-derived | 0.182692 | 0.547 | 0.694 | 0.173 | 2186/2186 |
| XGBoost | SAS + demographics | Test | Development-derived | 0.234043 | 0.669 | 0.800 | 0.142 | 2186/2186 |
| XGBoost | SCL-90 group + demographics | Test | Development-derived | 0.164179 | 0.627 | 0.786 | 0.144 | 2186/2186 |
| XGBoost | Full model/input | Test | Fixed 0.5 | 0.500000 | 0.674 | 0.846 | 0.129 | 2186/2186 |
| XGBoost | Leave out AAQ-II | Test | Fixed 0.5 | 0.500000 | 0.673 | 0.845 | 0.128 | 2186/2186 |
| XGBoost | Leave out CFQ | Test | Fixed 0.5 | 0.500000 | 0.674 | 0.841 | 0.129 | 2186/2186 |
| XGBoost | Leave out SAS | Test | Fixed 0.5 | 0.500000 | 0.606 | 0.821 | 0.137 | 2186/2186 |
| XGBoost | Leave out SCL-90 group | Test | Fixed 0.5 | 0.500000 | 0.623 | 0.828 | 0.139 | 2186/2186 |
| XGBoost | AAQ-II + demographics | Test | Fixed 0.5 | 0.500000 | 0.475 | 0.716 | 0.168 | 2186/2186 |
| XGBoost | CFQ + demographics | Test | Fixed 0.5 | 0.500000 | 0.437 | 0.694 | 0.173 | 2186/2186 |
| XGBoost | SAS + demographics | Test | Fixed 0.5 | 0.500000 | 0.662 | 0.800 | 0.142 | 2186/2186 |
| XGBoost | SCL-90 group + demographics | Test | Fixed 0.5 | 0.500000 | 0.712 | 0.786 | 0.144 | 2186/2186 |

**Panel C. Machine-learning ablation differences (variant minus full model)**

| **Model** | **Variant** | **Cohort** | **Delta Macro-F1 (95% CI)** | **Delta AUROC (95% CI)** | **Delta Brier (95% CI)** | **Hard-label n** | **Probability n** |
| --- | --- | --- | --- | --- | --- | --- | --- |
| LR | Leave out AAQ-II | Holdout | 0.008 (-0.000 to 0.016) | -0.005 (-0.008 to -0.002) | 0.001 (-0.000 to 0.003) | 2258 | 2258 |
| LR | Leave out CFQ | Holdout | 0.000 (0.000 to 0.000) | 0.000 (-0.000 to 0.000) | 0.000 (-0.000 to 0.000) | 2258 | 2258 |
| LR | Leave out SAS | Holdout | 0.027 (0.004 to 0.046) | -0.026 (-0.038 to -0.015) | 0.009 (0.005 to 0.014) | 2258 | 2258 |
| LR | Leave out SCL-90 group | Holdout | 0.040 (0.025 to 0.053) | -0.006 (-0.014 to 0.001) | 0.003 (-0.001 to 0.006) | 2258 | 2258 |
| LR | AAQ-II + demographics | Holdout | -0.065 (-0.088 to -0.043) | -0.105 (-0.126 to -0.084) | 0.036 (0.028 to 0.044) | 2258 | 2258 |
| LR | CFQ + demographics | Holdout | -0.106 (-0.126 to -0.085) | -0.138 (-0.160 to -0.116) | 0.042 (0.034 to 0.049) | 2258 | 2258 |
| LR | SAS + demographics | Holdout | -0.025 (-0.039 to -0.010) | -0.018 (-0.028 to -0.008) | 0.005 (0.000 to 0.010) | 2258 | 2258 |
| LR | SCL-90 group + demographics | Holdout | 0.031 (0.009 to 0.049) | -0.043 (-0.057 to -0.030) | 0.012 (0.006 to 0.017) | 2258 | 2258 |
| XGBoost | Leave out AAQ-II | Holdout | 0.034 (0.024 to 0.044) | -0.002 (-0.008 to 0.003) | -0.001 (-0.003 to 0.002) | 2258 | 2258 |
| XGBoost | Leave out CFQ | Holdout | 0.002 (-0.007 to 0.010) | -0.002 (-0.008 to 0.004) | -0.002 (-0.004 to 0.000) | 2258 | 2258 |
| XGBoost | Leave out SAS | Holdout | 0.050 (0.033 to 0.066) | -0.025 (-0.038 to -0.012) | 0.009 (0.005 to 0.013) | 2258 | 2258 |
| XGBoost | Leave out SCL-90 group | Holdout | 0.017 (0.001 to 0.033) | -0.018 (-0.029 to -0.006) | 0.009 (0.005 to 0.012) | 2258 | 2258 |
| XGBoost | AAQ-II + demographics | Holdout | -0.013 (-0.037 to 0.012) | -0.136 (-0.163 to -0.112) | 0.042 (0.035 to 0.049) | 2258 | 2258 |
| XGBoost | CFQ + demographics | Holdout | -0.133 (-0.153 to -0.111) | -0.148 (-0.171 to -0.126) | 0.043 (0.037 to 0.050) | 2258 | 2258 |
| XGBoost | SAS + demographics | Holdout | 0.001 (-0.018 to 0.019) | -0.047 (-0.062 to -0.031) | 0.013 (0.009 to 0.017) | 2258 | 2258 |
| XGBoost | SCL-90 group + demographics | Holdout | -0.039 (-0.056 to -0.022) | -0.064 (-0.081 to -0.048) | 0.017 (0.011 to 0.022) | 2258 | 2258 |
| LR | Leave out AAQ-II | Test | 0.002 (-0.006 to 0.010) | -0.003 (-0.006 to -0.000) | 0.000 (-0.001 to 0.002) | 2186 | 2186 |
| LR | Leave out CFQ | Test | 0.000 (0.000 to 0.000) | -0.000 (-0.000 to 0.000) | -0.000 (-0.000 to 0.000) | 2186 | 2186 |
| LR | Leave out SAS | Test | 0.046 (0.027 to 0.067) | -0.029 (-0.042 to -0.016) | 0.013 (0.007 to 0.018) | 2186 | 2186 |
| LR | Leave out SCL-90 group | Test | 0.034 (0.020 to 0.049) | -0.013 (-0.022 to -0.004) | 0.008 (0.004 to 0.012) | 2186 | 2186 |
| LR | AAQ-II + demographics | Test | -0.079 (-0.101 to -0.058) | -0.106 (-0.130 to -0.083) | 0.043 (0.036 to 0.051) | 2186 | 2186 |
| LR | CFQ + demographics | Test | -0.117 (-0.139 to -0.095) | -0.130 (-0.155 to -0.106) | 0.048 (0.040 to 0.057) | 2186 | 2186 |
| LR | SAS + demographics | Test | -0.029 (-0.046 to -0.014) | -0.031 (-0.043 to -0.018) | 0.012 (0.007 to 0.017) | 2186 | 2186 |
| LR | SCL-90 group + demographics | Test | 0.037 (0.020 to 0.057) | -0.046 (-0.061 to -0.030) | 0.014 (0.009 to 0.020) | 2186 | 2186 |
| XGBoost | Leave out AAQ-II | Test | 0.032 (0.020 to 0.043) | -0.002 (-0.008 to 0.004) | -0.001 (-0.003 to 0.002) | 2186 | 2186 |
| XGBoost | Leave out CFQ | Test | 0.000 (-0.008 to 0.008) | -0.006 (-0.011 to -0.000) | 0.000 (-0.002 to 0.002) | 2186 | 2186 |
| XGBoost | Leave out SAS | Test | 0.054 (0.039 to 0.071) | -0.025 (-0.040 to -0.011) | 0.008 (0.004 to 0.012) | 2186 | 2186 |
| XGBoost | Leave out SCL-90 group | Test | 0.015 (-0.001 to 0.030) | -0.019 (-0.029 to -0.007) | 0.010 (0.006 to 0.013) | 2186 | 2186 |
| XGBoost | AAQ-II + demographics | Test | -0.015 (-0.038 to 0.010) | -0.131 (-0.156 to -0.106) | 0.039 (0.032 to 0.046) | 2186 | 2186 |
| XGBoost | CFQ + demographics | Test | -0.113 (-0.134 to -0.090) | -0.152 (-0.177 to -0.126) | 0.044 (0.037 to 0.051) | 2186 | 2186 |
| XGBoost | SAS + demographics | Test | 0.009 (-0.009 to 0.026) | -0.046 (-0.063 to -0.030) | 0.014 (0.009 to 0.018) | 2186 | 2186 |
| XGBoost | SCL-90 group + demographics | Test | -0.033 (-0.048 to -0.019) | -0.060 (-0.078 to -0.043) | 0.015 (0.010 to 0.021) | 2186 | 2186 |

**Panel D. Exploratory Holdout-400 LLM input-ablation results at temperature=0**

| **Condition** | **Input variant** | **Threshold** | **Macro-F1** | **AUROC** | **Brier** | **Valid n/N** |
| --- | --- | --- | --- | --- | --- | --- |
| GPT E1 | Full model/input | 0.500000 | 0.717 | 0.788 | 0.167 | 400/400 |
| GPT E1 | Leave out SAS | 0.500000 | 0.692 | 0.780 | 0.201 | 400/400 |
| GPT E1 | Leave out CFQ | 0.500000 | 0.721 | 0.803 | 0.150 | 400/400 |
| GPT E1 | Leave out AAQ-II | 0.500000 | 0.707 | 0.785 | 0.165 | 400/400 |
| GPT E1 | Leave out SCL-90 group | 0.500000 | 0.672 | 0.787 | 0.153 | 400/400 |
| Gemini E2 | Full model/input | 0.500000 | 0.727 | 0.801 | 0.169 | 399/400 |
| Gemini E2 | Leave out SAS | 0.500000 | 0.699 | 0.785 | 0.185 | 400/400 |
| Gemini E2 | Leave out CFQ | 0.500000 | 0.734 | 0.807 | 0.169 | 399/400 |
| Gemini E2 | Leave out AAQ-II | 0.500000 | 0.724 | 0.800 | 0.175 | 400/400 |
| Gemini E2 | Leave out SCL-90 group | 0.500000 | 0.731 | 0.789 | 0.161 | 399/400 |

**Panel D (continued). Exploratory LLM input-ablation differences (ablated input minus full input)**

| **Condition** | **Input variant** | **Delta Macro-F1 (95% CI)** | **Delta AUROC (95% CI)** | **Delta Brier (95% CI)** | **Hard-label n** | **Probability n** |
| --- | --- | --- | --- | --- | --- | --- |
| GPT E1 | Leave out SAS | -0.024 (-0.060 to 0.012) | -0.008 (-0.023 to 0.008) | 0.035 (0.023 to 0.047) | 400 | 400 |
| GPT E1 | Leave out CFQ | 0.005 (-0.037 to 0.050) | 0.015 (-0.002 to 0.034) | -0.016 (-0.026 to -0.007) | 400 | 400 |
| GPT E1 | Leave out AAQ-II | -0.010 (-0.040 to 0.021) | -0.003 (-0.019 to 0.013) | -0.002 (-0.010 to 0.007) | 400 | 400 |
| GPT E1 | Leave out SCL-90 group | -0.045 (-0.105 to 0.010) | -0.000 (-0.038 to 0.033) | -0.014 (-0.031 to 0.004) | 400 | 400 |
| Gemini E2 | Leave out SAS | -0.028 (-0.060 to 0.006) | -0.016 (-0.035 to 0.001) | 0.017 (0.008 to 0.026) | 400 | 399 |
| Gemini E2 | Leave out CFQ | 0.006 (-0.014 to 0.027) | 0.008 (-0.002 to 0.017) | 0.000 (-0.005 to 0.005) | 400 | 398 |
| Gemini E2 | Leave out AAQ-II | -0.003 (-0.020 to 0.014) | -0.001 (-0.014 to 0.012) | 0.007 (0.001 to 0.013) | 400 | 399 |
| Gemini E2 | Leave out SCL-90 group | 0.004 (-0.033 to 0.038) | -0.013 (-0.040 to 0.011) | -0.006 (-0.017 to 0.004) | 400 | 398 |

***Note.*** *ML models were refitted after deleting a group with the prespecified preprocessing and hyperparameters; no new hyperparameter search was performed. Single-group models retained demographics. LLM target profiles and few-shot demonstrations had the same group removed. Ablations quantify predictive dependence under correlated inputs and are not causal attribution analyses.*

**Panel E. Demographics-only versus full-model comparisons**

| **Model** | **Cohort** | **Demographics-only AUROC** | **Full-model AUROC** | **ΔAUROC (95% CI)** | **Demographics-only Brier** | **Full-model Brier** | **ΔBrier (95% CI)** |
| --- | --- | --- | --- | --- | --- | --- | --- |
| LR | Test | 0.531 | 0.844 | −0.313 (−0.344 to −0.278) | 0.242 | 0.174 | 0.069 (0.060 to 0.078) |
| LR | Holdout | 0.538 | 0.832 | −0.294 (−0.329 to −0.264) | 0.238 | 0.179 | 0.058 (0.050 to 0.067) |
| XGBoost | Test | 0.498 | 0.846 | −0.349 (−0.382 to −0.316) | 0.207 | 0.129 | 0.078 (0.068 to 0.087) |
| XGBoost | Holdout | 0.521 | 0.835 | −0.314 (−0.345 to −0.283) | 0.236 | 0.147 | 0.089 (0.080 to 0.101) |

***Note.*** *Differences are demographics-only minus full model and use paired 1,000-resample bootstrap confidence intervals. Positive ΔBrier values indicate worse probability accuracy. Sex and age were the only demographic variables available. Because age differed materially across the grade cohorts, these comparisons are descriptive assessments of information sufficiency rather than causal attribution analyses.*

**Table S9.** Predictor distributions, outcome prevalence, and pairwise cohort-shift measures across Development, Test, and Holdout.

**Panel A. Complete cohort distributions**

| **Cohort** | **Variable** | **Type** | **n** | **Mean (SD)** | **Median [IQR]** | **Proportion (95% CI)** |
| --- | --- | --- | --- | --- | --- | --- |
| Development | age_years | continuous | 1951 | 19.336 (1.227) | 19.203 [18.592-19.936] | - |
| Development | sas_std | continuous | 1951 | 40.494 (6.227) | 39.000 [36.000-44.000] | - |
| Development | cfq_total | continuous | 1951 | 28.810 (8.137) | 30.000 [24.000-34.000] | - |
| Development | aaq2_total | continuous | 1951 | 20.261 (6.142) | 20.000 [16.000-24.000] | - |
| Development | scl90_somatization | continuous | 1951 | 1.308 (0.380) | 1.170 [1.000-1.420] | - |
| Development | scl90_obsessive_compulsive | continuous | 1951 | 1.942 (0.543) | 1.900 [1.500-2.300] | - |
| Development | scl90_interpersonal_sensitivity | continuous | 1951 | 1.752 (0.566) | 1.670 [1.330-2.110] | - |
| Development | scl90_anxiety | continuous | 1951 | 1.512 (0.468) | 1.400 [1.200-1.800] | - |
| Development | scl90_hostility | continuous | 1951 | 1.494 (0.499) | 1.330 [1.170-1.670] | - |
| Development | scl90_phobic_anxiety | continuous | 1951 | 1.454 (0.489) | 1.290 [1.140-1.710] | - |
| Development | scl90_paranoid_ideation | continuous | 1951 | 1.533 (0.492) | 1.500 [1.170-1.830] | - |
| Development | scl90_psychoticism | continuous | 1951 | 1.488 (0.434) | 1.400 [1.200-1.700] | - |
| Development | scl90_additional_items | continuous | 1951 | 1.524 (0.478) | 1.430 [1.140-1.860] | - |
| Development | sex_female | binary | 1951 | - | - | 54.5% (52.3% to 56.7%) |
| Development | sds_screen_positive | binary | 1951 | - | - | 16.3% (14.7% to 18.0%) |
| Test | age_years | continuous | 2186 | 20.398 (0.961) | 20.290 [19.708-20.951] | - |
| Test | sas_std | continuous | 2186 | 41.816 (7.045) | 40.000 [36.000-46.000] | - |
| Test | cfq_total | continuous | 2186 | 29.946 (8.371) | 31.000 [25.000-35.000] | - |
| Test | aaq2_total | continuous | 2186 | 21.275 (6.365) | 21.000 [17.000-26.000] | - |
| Test | scl90_somatization | continuous | 2186 | 1.385 (0.452) | 1.250 [1.080-1.580] | - |
| Test | scl90_obsessive_compulsive | continuous | 2186 | 1.989 (0.601) | 1.900 [1.500-2.400] | - |
| Test | scl90_interpersonal_sensitivity | continuous | 2186 | 1.824 (0.614) | 1.780 [1.330-2.220] | - |
| Test | scl90_anxiety | continuous | 2186 | 1.596 (0.521) | 1.500 [1.200-1.900] | - |
| Test | scl90_hostility | continuous | 2186 | 1.582 (0.562) | 1.500 [1.170-1.830] | - |
| Test | scl90_phobic_anxiety | continuous | 2186 | 1.512 (0.547) | 1.290 [1.140-1.823] | - |
| Test | scl90_paranoid_ideation | continuous | 2186 | 1.597 (0.535) | 1.500 [1.170-1.830] | - |
| Test | scl90_psychoticism | continuous | 2186 | 1.552 (0.501) | 1.400 [1.200-1.800] | - |
| Test | scl90_additional_items | continuous | 2186 | 1.607 (0.534) | 1.430 [1.140-1.860] | - |
| Test | sex_female | binary | 2186 | - | - | 51.5% (49.4% to 53.6%) |
| Test | sds_screen_positive | binary | 2186 | - | - | 24.4% (22.7% to 26.3%) |
| Holdout | age_years | continuous | 2258 | 21.487 (0.994) | 21.386 [20.770-22.076] | - |
| Holdout | sas_std | continuous | 2258 | 42.132 (7.077) | 41.000 [36.000-46.000] | - |
| Holdout | cfq_total | continuous | 2258 | 30.307 (8.731) | 31.000 [25.000-36.000] | - |
| Holdout | aaq2_total | continuous | 2258 | 21.368 (6.681) | 21.000 [17.000-26.000] | - |
| Holdout | scl90_somatization | continuous | 2258 | 1.384 (0.439) | 1.250 [1.080-1.580] | - |
| Holdout | scl90_obsessive_compulsive | continuous | 2258 | 1.964 (0.593) | 1.900 [1.500-2.300] | - |
| Holdout | scl90_interpersonal_sensitivity | continuous | 2258 | 1.813 (0.615) | 1.780 [1.330-2.220] | - |
| Holdout | scl90_anxiety | continuous | 2258 | 1.594 (0.523) | 1.500 [1.200-1.900] | - |
| Holdout | scl90_hostility | continuous | 2258 | 1.585 (0.555) | 1.500 [1.170-1.830] | - |
| Holdout | scl90_phobic_anxiety | continuous | 2258 | 1.482 (0.511) | 1.290 [1.140-1.710] | - |
| Holdout | scl90_paranoid_ideation | continuous | 2258 | 1.596 (0.534) | 1.500 [1.170-1.830] | - |
| Holdout | scl90_psychoticism | continuous | 2258 | 1.546 (0.475) | 1.400 [1.200-1.800] | - |
| Holdout | scl90_additional_items | continuous | 2258 | 1.618 (0.525) | 1.570 [1.140-2.000] | - |
| Holdout | sex_female | binary | 2258 | - | - | 50.3% (48.2% to 52.4%) |
| Holdout | sds_screen_positive | binary | 2258 | - | - | 27.9% (26.1% to 29.8%) |

**Panel B. Pairwise standardized mean differences and distribution-shape statistics**

| **Cohort comparison** | **Variable** | **SMD** | **Absolute SMD** | **KS statistic** | **Absolute prevalence difference** |
| --- | --- | --- | --- | --- | --- |
| Development vs Test | age_years | -0.964 | 0.964 | 0.483 |  |
| Development vs Test | sas_std | -0.199 | 0.199 | 0.096 |  |
| Development vs Test | cfq_total | -0.138 | 0.138 | 0.073 |  |
| Development vs Test | aaq2_total | -0.162 | 0.162 | 0.067 |  |
| Development vs Test | scl90_somatization | -0.183 | 0.183 | 0.070 |  |
| Development vs Test | scl90_obsessive_compulsive | -0.083 | 0.083 | 0.054 |  |
| Development vs Test | scl90_interpersonal_sensitivity | -0.122 | 0.122 | 0.056 |  |
| Development vs Test | scl90_anxiety | -0.171 | 0.171 | 0.077 |  |
| Development vs Test | scl90_hostility | -0.164 | 0.164 | 0.070 |  |
| Development vs Test | scl90_phobic_anxiety | -0.111 | 0.111 | 0.046 |  |
| Development vs Test | scl90_paranoid_ideation | -0.124 | 0.124 | 0.048 |  |
| Development vs Test | scl90_psychoticism | -0.137 | 0.137 | 0.067 |  |
| Development vs Test | scl90_additional_items | -0.165 | 0.165 | 0.075 |  |
| Development vs Test | sex_female | 0.061 | 0.061 |  | 0.030 |
| Development vs Test | sds_screen_positive | -0.203 | 0.203 |  | 0.081 |
| Development vs Holdout | age_years | -1.926 | 1.926 | 0.754 |  |
| Development vs Holdout | sas_std | -0.246 | 0.246 | 0.110 |  |
| Development vs Holdout | cfq_total | -0.177 | 0.177 | 0.080 |  |
| Development vs Holdout | aaq2_total | -0.172 | 0.172 | 0.071 |  |
| Development vs Holdout | scl90_somatization | -0.183 | 0.183 | 0.072 |  |
| Development vs Holdout | scl90_obsessive_compulsive | -0.039 | 0.039 | 0.031 |  |
| Development vs Holdout | scl90_interpersonal_sensitivity | -0.102 | 0.102 | 0.054 |  |
| Development vs Holdout | scl90_anxiety | -0.166 | 0.166 | 0.070 |  |
| Development vs Holdout | scl90_hostility | -0.172 | 0.172 | 0.070 |  |
| Development vs Holdout | scl90_phobic_anxiety | -0.057 | 0.057 | 0.029 |  |
| Development vs Holdout | scl90_paranoid_ideation | -0.123 | 0.123 | 0.052 |  |
| Development vs Holdout | scl90_psychoticism | -0.128 | 0.128 | 0.071 |  |
| Development vs Holdout | scl90_additional_items | -0.187 | 0.187 | 0.084 |  |
| Development vs Holdout | sex_female | 0.084 | 0.084 |  | 0.042 |
| Development vs Holdout | sds_screen_positive | -0.283 | 0.283 |  | 0.116 |
| Test vs Holdout | age_years | -1.114 | 1.114 | 0.471 |  |
| Test vs Holdout | sas_std | -0.045 | 0.045 | 0.033 |  |
| Test vs Holdout | cfq_total | -0.042 | 0.042 | 0.018 |  |
| Test vs Holdout | aaq2_total | -0.014 | 0.014 | 0.014 |  |
| Test vs Holdout | scl90_somatization | 0.003 | 0.003 | 0.024 |  |
| Test vs Holdout | scl90_obsessive_compulsive | 0.042 | 0.042 | 0.031 |  |
| Test vs Holdout | scl90_interpersonal_sensitivity | 0.018 | 0.018 | 0.018 |  |
| Test vs Holdout | scl90_anxiety | 0.004 | 0.004 | 0.015 |  |
| Test vs Holdout | scl90_hostility | -0.007 | 0.007 | 0.014 |  |
| Test vs Holdout | scl90_phobic_anxiety | 0.056 | 0.056 | 0.020 |  |
| Test vs Holdout | scl90_paranoid_ideation | 0.001 | 0.001 | 0.009 |  |
| Test vs Holdout | scl90_psychoticism | 0.012 | 0.012 | 0.021 |  |
| Test vs Holdout | scl90_additional_items | -0.020 | 0.020 | 0.024 |  |
| Test vs Holdout | sex_female | 0.023 | 0.023 |  | 0.012 |
| Test vs Holdout | sds_screen_positive | -0.080 | 0.080 |  | 0.035 |

***Note.*** *Continuous variables are summarized by mean (SD) and median [IQR]; binary variables are shown as proportions with Wilson 95% confidence intervals. SMD quantifies standardized location differences, while the two-sample Kolmogorov-Smirnov statistic summarizes continuous distribution-shape differences. Large-sample per-variable P values were not used as the primary drift criterion.*

**Table S10.** Enhanced probability-calibration results under model-native and common-valid denominators.

| **Cohort** | **Condition** | **Denominator** | **n (positive)** | **Brier (95% CI)** | **ECE (95% CI)** | **Calibration intercept (95% CI)** | **Calibration slope (95% CI)** |
| --- | --- | --- | --- | --- | --- | --- | --- |
| Test | LR | native valid | 2186 (534) | 0.174 (0.165 to 0.183) | 0.204 (0.189 to 0.219) | -1.391 (-1.520 to -1.262) | 0.964 (0.874 to 1.053) |
| Test | LR | common valid | 2182 (532) | 0.174 (0.165 to 0.182) | 0.204 (0.190 to 0.221) | -1.395 (-1.523 to -1.266) | 0.963 (0.874 to 1.053) |
| Test | XGBoost | native valid | 2186 (534) | 0.129 (0.119 to 0.138) | 0.042 (0.029 to 0.057) | 0.248 (0.080 to 0.416) | 0.926 (0.823 to 1.029) |
| Test | XGBoost | common valid | 2182 (532) | 0.128 (0.119 to 0.137) | 0.042 (0.029 to 0.057) | 0.244 (0.076 to 0.412) | 0.925 (0.822 to 1.028) |
| Test | GPT E1 | native valid | 2186 (534) | 0.157 (0.148 to 0.166) | 0.144 (0.129 to 0.160) | -0.953 (-1.068 to -0.838) | 0.891 (0.803 to 0.978) |
| Test | GPT E1 | common valid | 2182 (532) | 0.156 (0.147 to 0.165) | 0.144 (0.130 to 0.159) | -0.956 (-1.071 to -0.841) | 0.892 (0.804 to 0.980) |
| Test | Gemini E2 | native valid | 2182 (532) | 0.167 (0.158 to 0.176) | 0.186 (0.171 to 0.201) | -1.213 (-1.336 to -1.090) | 1.040 (0.945 to 1.134) |
| Test | Gemini E2 | common valid | 2182 (532) | 0.167 (0.158 to 0.176) | 0.186 (0.171 to 0.201) | -1.213 (-1.336 to -1.090) | 1.040 (0.945 to 1.134) |
| Holdout | LR | native valid | 2258 (631) | 0.179 (0.170 to 0.188) | 0.187 (0.172 to 0.202) | -1.200 (-1.318 to -1.082) | 0.927 (0.842 to 1.012) |
| Holdout | LR | common valid | 2256 (631) | 0.179 (0.170 to 0.189) | 0.186 (0.172 to 0.201) | -1.198 (-1.316 to -1.080) | 0.928 (0.843 to 1.013) |
| Holdout | XGBoost | native valid | 2258 (631) | 0.147 (0.138 to 0.156) | 0.061 (0.047 to 0.078) | 0.455 (0.297 to 0.614) | 1.018 (0.913 to 1.123) |
| Holdout | XGBoost | common valid | 2256 (631) | 0.147 (0.138 to 0.155) | 0.061 (0.047 to 0.076) | 0.458 (0.299 to 0.616) | 1.018 (0.914 to 1.123) |
| Holdout | GPT E1 | native valid | 2258 (631) | 0.160 (0.151 to 0.169) | 0.113 (0.098 to 0.129) | -0.711 (-0.819 to -0.604) | 0.856 (0.773 to 0.939) |
| Holdout | GPT E1 | common valid | 2256 (631) | 0.160 (0.151 to 0.169) | 0.113 (0.100 to 0.129) | -0.708 (-0.816 to -0.601) | 0.858 (0.775 to 0.941) |
| Holdout | Gemini E2 | native valid | 2256 (631) | 0.167 (0.158 to 0.176) | 0.150 (0.136 to 0.165) | -0.930 (-1.041 to -0.818) | 1.004 (0.916 to 1.093) |
| Holdout | Gemini E2 | common valid | 2256 (631) | 0.167 (0.158 to 0.176) | 0.150 (0.136 to 0.165) | -0.930 (-1.041 to -0.818) | 1.004 (0.916 to 1.093) |

***Note.*** *The ECE values shown in this table use 10 equal-frequency bins; the prespecified primary ECE used 10 equal-width bins. Supplementary Figure S6 reports equal-width and equal-frequency sensitivity over 5, 10, 15, and 20 bins. Calibration intercept and slope were estimated after clipping probabilities to [1e-6, 1-1e-6] and applying the logit transform. Ideal values are 0 and 1, respectively. Model-native denominators retain each model's valid outputs; common-valid rows use the four-model intersection.*

**Table S11.** Repeated-output stability, unstable-case localization, exploratory mitigation analyses, and cross-window backend-drift audit for the two LLM finalists.

**Panel A. Principal repeated-output stability summaries**

| **Condition** | **Call window** | **Temperature** | **Cases** | **Inconsistent n/N; rate or paired difference (95% CI)** | **Mean entropy** | **Mean vote margin** | **Probability range median [IQR]** | **Mode Macro-F1** |
| --- | --- | --- | --- | --- | --- | --- | --- | --- |
| GPT E1 | primary complete Holdout | 0.1 | 2258 | 532/2258; 23.6% (21.9% to 25.4%) | 0.172 | 0.885 | 0.100 [0.070-0.200] | 0.734 |
| GPT E1 | supplementary representative 400 | 0.0 | 400 | 62/400; 15.5% (12.3% to 19.4%) | 0.127 | 0.913 | 0.070 [0.050-0.110] | 0.736 |
| GPT E1 | supplementary representative 400 | 0.1 | 400 | 66/400; 16.5% (13.2% to 20.5%) | 0.137 | 0.905 | 0.073 [0.050-0.120] | 0.744 |
| GPT E1 | temperature paired difference T0 minus T0.1 |  | 400 | Difference: -1.00% (-4.25% to 2.25%) |  |  | [-] |  |
| Gemini E2 | primary complete Holdout | 0.1 | 2258 | 34/2258; 1.5% (1.1% to 2.1%) | 0.009 | 0.995 | 0.000 [0.000-0.000] | 0.739 |
| Gemini E2 | supplementary representative 400 | 0.0 | 400 | 4/400; 1.0% (0.4% to 2.5%) | 0.007 | 0.996 | 0.000 [0.000-0.000] | 0.730 |
| Gemini E2 | supplementary representative 400 | 0.1 | 400 | 3/400; 0.8% (0.3% to 2.2%) | 0.006 | 0.996 | 0.000 [0.000-0.000] | 0.730 |
| Gemini E2 | temperature paired difference T0 minus T0.1 |  | 400 | Difference: 0.25% (-1.00% to 1.50%) |  |  | [-] |  |

**Panel B. Instability by threshold-distance band, observed outcome, and sex**

| **Condition** | **Call window** | **Analysis** | **Group** | **n** | **Inconsistent n/N; rate (95% CI)** |
| --- | --- | --- | --- | --- | --- |
| GPT E1 | primary complete Holdout | distance to 0.5 | <0.05 | 47 | 47/47; 100.0% (92.4% to 100.0%) |
| GPT E1 | primary complete Holdout | distance to 0.5 | 0.05-<0.10 | 129 | 118/129; 91.5% (85.4% to 95.2%) |
| GPT E1 | primary complete Holdout | distance to 0.5 | 0.10-<0.20 | 585 | 316/585; 54.0% (50.0% to 58.0%) |
| GPT E1 | primary complete Holdout | distance to 0.5 | >=0.20 | 1497 | 51/1497; 3.4% (2.6% to 4.5%) |
| GPT E1 | primary complete Holdout | outcome | 0 | 1627 | 339/1627; 20.8% (18.9% to 22.9%) |
| GPT E1 | primary complete Holdout | outcome | 1 | 631 | 193/631; 30.6% (27.1% to 34.3%) |
| GPT E1 | primary complete Holdout | sex | female | 1136 | 275/1136; 24.2% (21.8% to 26.8%) |
| GPT E1 | primary complete Holdout | sex | male | 1122 | 257/1122; 22.9% (20.5% to 25.5%) |
| Gemini E2 | primary complete Holdout | distance to 0.5 | <0.05 | 0 | Not estimable (n=0) |
| Gemini E2 | primary complete Holdout | distance to 0.5 | 0.05-<0.10 | 53 | 13/53; 24.5% (14.9% to 37.6%) |
| Gemini E2 | primary complete Holdout | distance to 0.5 | 0.10-<0.20 | 613 | 11/613; 1.8% (1.0% to 3.2%) |
| Gemini E2 | primary complete Holdout | distance to 0.5 | >=0.20 | 1592 | 10/1592; 0.6% (0.3% to 1.2%) |
| Gemini E2 | primary complete Holdout | outcome | 0 | 1627 | 24/1627; 1.5% (1.0% to 2.2%) |
| Gemini E2 | primary complete Holdout | outcome | 1 | 631 | 10/631; 1.6% (0.9% to 2.9%) |
| Gemini E2 | primary complete Holdout | sex | female | 1136 | 19/1136; 1.7% (1.1% to 2.6%) |
| Gemini E2 | primary complete Holdout | sex | male | 1122 | 15/1122; 1.3% (0.8% to 2.2%) |

**Panel C. April versus August 2026 temperature=0.1 call-window audit**

| **Condition** | **n** | **Label agreement** | **Probability MAE** | **Probability Spearman** | **Common n** | **Interpretation** |
| --- | --- | --- | --- | --- | --- | --- |
| GPT E1 | 400 | 96.8% | 0.027 | 0.991 | 400 | High agreement; not a strict frozen-version replication |
| Gemini E2 | 400 | 98.3% | 0.016 | 0.983 | 400 | High agreement; not a strict frozen-version replication |

***Note.*** *An inconsistent case had at least two distinct valid repeated hard labels or any prespecified invalid-output contribution. Rate intervals are Wilson 95% intervals. Participant-level median probability and range summarize valid repeated probabilities. Distances were rounded to 12 decimal places before assigning half-open bands; rates and intervals were not estimated for empty groups. The call-window comparison is an audit of hosted-backend drift, not a strict frozen-version replication.*

**Panel D. Exploratory cumulative probability-margin review**

| **Condition** | **Margin** | **Deferred n/N (%)** | **Inconsistent cases captured n/N (%)** | **Retained coverage n/N (%)** | **Retained inconsistent n/N (%)** |
| --- | --- | --- | --- | --- | --- |
| GPT E1 | 0.05 | 47/2,258 (2.1%) | 47/532 (8.8%) | 2,211/2,258 (97.9%) | 485/2,211 (21.9%) |
| GPT E1 | 0.10 | 176/2,258 (7.8%) | 165/532 (31.0%) | 2,082/2,258 (92.2%) | 367/2,082 (17.6%) |
| GPT E1 | 0.20 | 761/2,258 (33.7%) | 481/532 (90.4%) | 1,497/2,258 (66.3%) | 51/1,497 (3.4%) |
| Gemini E2 | 0.05 | 0/2,258 (0.0%) | 0/34 (0.0%) | 2,258/2,258 (100.0%) | 34/2,258 (1.5%) |
| Gemini E2 | 0.10 | 53/2,258 (2.3%) | 13/34 (38.2%) | 2,205/2,258 (97.7%) | 21/2,205 (1.0%) |
| Gemini E2 | 0.20 | 666/2,258 (29.5%) | 24/34 (70.6%) | 1,592/2,258 (70.5%) | 10/1,592 (0.6%) |

**Panel E. Exploratory repeated-call majority-vote aggregation**

| **Condition** | **Repeats** | **Mean single-call macro-F1** | **Majority-vote macro-F1** | **Majority minus mean single-call macro-F1 (95% CI)** |
| --- | --- | --- | --- | --- |
| GPT E1 | 10 | 0.7296 | 0.7343 | 0.0046 (−0.0015 to 0.0110) |
| Gemini E2 | 10 | 0.7381 | 0.7395 | 0.0014 (0.0002 to 0.0025) |

***Note.*** *Probability margins were defined as the absolute distance between each participant’s median valid repeated probability and 0.5. LLM probabilities were not post-hoc calibrated. The margin analyses are retrospective selective-review scenarios rather than validated clinical utility or abstention rules. Majority-vote differences compare modal-label macro-F1 with the mean macro-F1 across ten individual calls; confidence intervals use participant-level bootstrap resampling.*

**Table S12.** Exploratory sex-stratified performance, calibration, output validity, and female-minus-male differences.

**Panel A. Sex-stratified performance and LLM operational validity**

| **Cohort** | **Condition** | **Strategy** | **Sex** | **n (positive)** | **Threshold** | **Sensitivity (95% CI)** | **Specificity (95% CI)** | **Brier (95% CI)** | **Valid-output rate (95% CI)** |
| --- | --- | --- | --- | --- | --- | --- | --- | --- | --- |
| Test | LR | Fixed 0.5 | Female | 1125 (301) | 0.500000 | 0.821 (0.774 to 0.866) | 0.701 (0.669 to 0.732) | 0.185 (0.173 to 0.198) | 1.000 (0.997 to 1.000) |
| Test | LR | Fixed 0.5 | Male | 1061 (233) | 0.500000 | 0.738 (0.680 to 0.793) | 0.772 (0.742 to 0.802) | 0.162 (0.149 to 0.175) | 1.000 (0.996 to 1.000) |
| Test | LR | Development-derived | Female | 1125 (301) | 0.466260 | 0.831 (0.786 to 0.874) | 0.664 (0.632 to 0.694) | 0.185 (0.173 to 0.198) | 1.000 (0.997 to 1.000) |
| Test | LR | Development-derived | Male | 1061 (233) | 0.466260 | 0.764 (0.708 to 0.813) | 0.750 (0.721 to 0.780) | 0.162 (0.149 to 0.175) | 1.000 (0.996 to 1.000) |
| Test | XGBoost | Fixed 0.5 | Female | 1125 (301) | 0.500000 | 0.362 (0.309 to 0.416) | 0.976 (0.965 to 0.986) | 0.137 (0.124 to 0.150) | 1.000 (0.997 to 1.000) |
| Test | XGBoost | Fixed 0.5 | Male | 1061 (233) | 0.500000 | 0.258 (0.203 to 0.318) | 0.983 (0.974 to 0.991) | 0.120 (0.107 to 0.134) | 1.000 (0.996 to 1.000) |
| Test | XGBoost | Development-derived | Female | 1125 (301) | 0.173913 | 0.874 (0.834 to 0.910) | 0.575 (0.542 to 0.609) | 0.137 (0.124 to 0.150) | 1.000 (0.997 to 1.000) |
| Test | XGBoost | Development-derived | Male | 1061 (233) | 0.173913 | 0.867 (0.822 to 0.909) | 0.668 (0.635 to 0.697) | 0.120 (0.107 to 0.134) | 1.000 (0.996 to 1.000) |
| Test | GPT E1 | Fixed 0.5 | Female | 1125 (301) | 0.500000 | 0.718 (0.671 to 0.768) | 0.784 (0.756 to 0.811) | 0.167 (0.155 to 0.179) | 1.000 (0.997 to 1.000) |
| Test | GPT E1 | Fixed 0.5 | Male | 1061 (233) | 0.500000 | 0.657 (0.600 to 0.715) | 0.850 (0.825 to 0.874) | 0.146 (0.134 to 0.158) | 1.000 (0.996 to 1.000) |
| Test | GPT E1 | Development-derived | Female | 1125 (301) | 0.380000 | 0.787 (0.742 to 0.835) | 0.699 (0.668 to 0.730) | 0.167 (0.155 to 0.179) | 1.000 (0.997 to 1.000) |
| Test | GPT E1 | Development-derived | Male | 1061 (233) | 0.380000 | 0.730 (0.677 to 0.784) | 0.775 (0.748 to 0.803) | 0.146 (0.134 to 0.158) | 1.000 (0.996 to 1.000) |
| Test | Gemini E2 | Fixed 0.5 | Female | 1125 (301) | 0.500000 | 0.757 (0.710 to 0.806) | 0.765 (0.736 to 0.793) | 0.175 (0.163 to 0.187) | 1.000 (0.997 to 1.000) |
| Test | Gemini E2 | Fixed 0.5 | Male | 1061 (233) | 0.500000 | 0.721 (0.664 to 0.776) | 0.810 (0.783 to 0.836) | 0.158 (0.146 to 0.172) | 0.996 (0.990 to 0.999) |
| Test | Gemini E2 | Development-derived | Female | 1125 (301) | 0.500000 | 0.757 (0.710 to 0.806) | 0.765 (0.736 to 0.793) | 0.175 (0.163 to 0.187) | 1.000 (0.997 to 1.000) |
| Test | Gemini E2 | Development-derived | Male | 1061 (233) | 0.500000 | 0.721 (0.664 to 0.776) | 0.810 (0.783 to 0.836) | 0.158 (0.146 to 0.172) | 0.996 (0.990 to 0.999) |
| Holdout | LR | Fixed 0.5 | Female | 1136 (353) | 0.500000 | 0.822 (0.781 to 0.862) | 0.687 (0.655 to 0.720) | 0.182 (0.169 to 0.194) | 1.000 (0.997 to 1.000) |
| Holdout | LR | Fixed 0.5 | Male | 1122 (278) | 0.500000 | 0.737 (0.685 to 0.785) | 0.750 (0.722 to 0.778) | 0.177 (0.165 to 0.190) | 1.000 (0.997 to 1.000) |
| Holdout | LR | Development-derived | Female | 1136 (353) | 0.466260 | 0.841 (0.802 to 0.877) | 0.644 (0.611 to 0.675) | 0.182 (0.169 to 0.194) | 1.000 (0.997 to 1.000) |
| Holdout | LR | Development-derived | Male | 1122 (278) | 0.466260 | 0.770 (0.720 to 0.816) | 0.717 (0.688 to 0.746) | 0.177 (0.165 to 0.190) | 1.000 (0.997 to 1.000) |
| Holdout | XGBoost | Fixed 0.5 | Female | 1136 (353) | 0.500000 | 0.306 (0.262 to 0.357) | 0.974 (0.962 to 0.986) | 0.156 (0.143 to 0.169) | 1.000 (0.997 to 1.000) |
| Holdout | XGBoost | Fixed 0.5 | Male | 1122 (278) | 0.500000 | 0.216 (0.164 to 0.266) | 0.977 (0.967 to 0.987) | 0.138 (0.126 to 0.150) | 1.000 (0.997 to 1.000) |
| Holdout | XGBoost | Development-derived | Female | 1136 (353) | 0.173913 | 0.887 (0.854 to 0.918) | 0.580 (0.544 to 0.614) | 0.156 (0.143 to 0.169) | 1.000 (0.997 to 1.000) |
| Holdout | XGBoost | Development-derived | Male | 1122 (278) | 0.173913 | 0.867 (0.823 to 0.906) | 0.578 (0.544 to 0.611) | 0.138 (0.126 to 0.150) | 1.000 (0.997 to 1.000) |
| Holdout | GPT E1 | Fixed 0.5 | Female | 1136 (353) | 0.500000 | 0.711 (0.663 to 0.758) | 0.802 (0.771 to 0.832) | 0.160 (0.148 to 0.172) | 1.000 (0.997 to 1.000) |
| Holdout | GPT E1 | Fixed 0.5 | Male | 1122 (278) | 0.500000 | 0.583 (0.520 to 0.640) | 0.838 (0.812 to 0.861) | 0.161 (0.149 to 0.175) | 1.000 (0.997 to 1.000) |
| Holdout | GPT E1 | Development-derived | Female | 1136 (353) | 0.380000 | 0.782 (0.738 to 0.825) | 0.720 (0.687 to 0.752) | 0.160 (0.148 to 0.172) | 1.000 (0.997 to 1.000) |
| Holdout | GPT E1 | Development-derived | Male | 1122 (278) | 0.380000 | 0.705 (0.649 to 0.757) | 0.750 (0.721 to 0.777) | 0.161 (0.149 to 0.175) | 1.000 (0.997 to 1.000) |
| Holdout | Gemini E2 | Fixed 0.5 | Female | 1136 (353) | 0.500000 | 0.751 (0.705 to 0.797) | 0.789 (0.759 to 0.817) | 0.163 (0.151 to 0.176) | 0.998 (0.994 to 1.000) |
| Holdout | Gemini E2 | Fixed 0.5 | Male | 1122 (278) | 0.500000 | 0.687 (0.630 to 0.742) | 0.802 (0.775 to 0.828) | 0.170 (0.159 to 0.183) | 1.000 (0.997 to 1.000) |
| Holdout | Gemini E2 | Development-derived | Female | 1136 (353) | 0.500000 | 0.751 (0.705 to 0.797) | 0.789 (0.759 to 0.817) | 0.163 (0.151 to 0.176) | 0.998 (0.994 to 1.000) |
| Holdout | Gemini E2 | Development-derived | Male | 1122 (278) | 0.500000 | 0.687 (0.630 to 0.742) | 0.802 (0.775 to 0.828) | 0.170 (0.159 to 0.183) | 1.000 (0.997 to 1.000) |

**Panel B. Sex-stratified calibration**

| **Cohort** | **Condition** | **Strategy** | **Sex** | **Calibration intercept (95% CI)** | **Calibration slope (95% CI)** | **Backend** |
| --- | --- | --- | --- | --- | --- | --- |
| Test | LR | Fixed 0.5 | Female | -1.449 (-1.633 to -1.266) | 1.002 (0.876 to 1.129) | statsmodels_glm |
| Test | LR | Fixed 0.5 | Male | -1.337 (-1.519 to -1.156) | 0.928 (0.801 to 1.056) | statsmodels_glm |
| Test | LR | Development-derived | Female | -1.449 (-1.633 to -1.266) | 1.002 (0.876 to 1.129) | statsmodels_glm |
| Test | LR | Development-derived | Male | -1.337 (-1.519 to -1.156) | 0.928 (0.801 to 1.056) | statsmodels_glm |
| Test | XGBoost | Fixed 0.5 | Female | 0.161 (-0.060 to 0.383) | 0.859 (0.720 to 0.997) | statsmodels_glm |
| Test | XGBoost | Fixed 0.5 | Male | 0.361 (0.103 to 0.619) | 1.005 (0.850 to 1.159) | statsmodels_glm |
| Test | XGBoost | Development-derived | Female | 0.161 (-0.060 to 0.383) | 0.859 (0.720 to 0.997) | statsmodels_glm |
| Test | XGBoost | Development-derived | Male | 0.361 (0.103 to 0.619) | 1.005 (0.850 to 1.159) | statsmodels_glm |
| Test | GPT E1 | Fixed 0.5 | Female | -0.954 (-1.111 to -0.798) | 0.892 (0.772 to 1.012) | statsmodels_glm |
| Test | GPT E1 | Fixed 0.5 | Male | -0.951 (-1.122 to -0.781) | 0.889 (0.759 to 1.019) | statsmodels_glm |
| Test | GPT E1 | Development-derived | Female | -0.954 (-1.111 to -0.798) | 0.892 (0.772 to 1.012) | statsmodels_glm |
| Test | GPT E1 | Development-derived | Male | -0.951 (-1.122 to -0.781) | 0.889 (0.759 to 1.019) | statsmodels_glm |
| Test | Gemini E2 | Fixed 0.5 | Female | -1.196 (-1.367 to -1.025) | 1.052 (0.922 to 1.183) | statsmodels_glm |
| Test | Gemini E2 | Fixed 0.5 | Male | -1.236 (-1.414 to -1.057) | 1.022 (0.886 to 1.159) | statsmodels_glm |
| Test | Gemini E2 | Development-derived | Female | -1.196 (-1.367 to -1.025) | 1.052 (0.922 to 1.183) | statsmodels_glm |
| Test | Gemini E2 | Development-derived | Male | -1.236 (-1.414 to -1.057) | 1.022 (0.886 to 1.159) | statsmodels_glm |
| Holdout | LR | Fixed 0.5 | Female | -1.202 (-1.371 to -1.033) | 0.997 (0.872 to 1.121) | statsmodels_glm |
| Holdout | LR | Fixed 0.5 | Male | -1.213 (-1.379 to -1.047) | 0.856 (0.740 to 0.972) | statsmodels_glm |
| Holdout | LR | Development-derived | Female | -1.202 (-1.371 to -1.033) | 0.997 (0.872 to 1.121) | statsmodels_glm |
| Holdout | LR | Development-derived | Male | -1.213 (-1.379 to -1.047) | 0.856 (0.740 to 0.972) | statsmodels_glm |
| Holdout | XGBoost | Fixed 0.5 | Female | 0.407 (0.195 to 0.620) | 0.892 (0.758 to 1.026) | statsmodels_glm |
| Holdout | XGBoost | Fixed 0.5 | Male | 0.530 (0.291 to 0.769) | 1.177 (1.010 to 1.344) | statsmodels_glm |
| Holdout | XGBoost | Development-derived | Female | 0.407 (0.195 to 0.620) | 0.892 (0.758 to 1.026) | statsmodels_glm |
| Holdout | XGBoost | Development-derived | Male | 0.530 (0.291 to 0.769) | 1.177 (1.010 to 1.344) | statsmodels_glm |
| Holdout | GPT E1 | Fixed 0.5 | Female | -0.656 (-0.807 to -0.505) | 0.958 (0.834 to 1.082) | statsmodels_glm |
| Holdout | GPT E1 | Fixed 0.5 | Male | -0.792 (-0.947 to -0.637) | 0.753 (0.641 to 0.866) | statsmodels_glm |
| Holdout | GPT E1 | Development-derived | Female | -0.656 (-0.807 to -0.505) | 0.958 (0.834 to 1.082) | statsmodels_glm |
| Holdout | GPT E1 | Development-derived | Male | -0.792 (-0.947 to -0.637) | 0.753 (0.641 to 0.866) | statsmodels_glm |
| Holdout | Gemini E2 | Fixed 0.5 | Female | -0.865 (-1.024 to -0.706) | 1.098 (0.969 to 1.227) | statsmodels_glm |
| Holdout | Gemini E2 | Fixed 0.5 | Male | -1.010 (-1.167 to -0.853) | 0.904 (0.781 to 1.027) | statsmodels_glm |
| Holdout | Gemini E2 | Development-derived | Female | -0.865 (-1.024 to -0.706) | 1.098 (0.969 to 1.227) | statsmodels_glm |
| Holdout | Gemini E2 | Development-derived | Male | -1.010 (-1.167 to -0.853) | 0.904 (0.781 to 1.027) | statsmodels_glm |

**Panel C. Female-minus-male differences with independent within-group bootstrap resampling**

| **Cohort** | **Condition** | **Strategy** | **Metric** | **Female** | **Male** | **Female minus male (95% CI)** | **Female/Male n** |
| --- | --- | --- | --- | --- | --- | --- | --- |
| Test | LR | Fixed 0.5 | Sensitivity | 0.821 | 0.738 | 0.082 (0.013 to 0.157) | 1125/1061 |
| Test | LR | Fixed 0.5 | Specificity | 0.701 | 0.772 | -0.070 (-0.116 to -0.031) | 1125/1061 |
| Test | LR | Fixed 0.5 | Brier score | 0.185 | 0.162 | 0.023 (0.006 to 0.042) | 1125/1061 |
| Test | LR | Development-derived | Sensitivity | 0.831 | 0.764 | 0.067 (-0.001 to 0.139) | 1125/1061 |
| Test | LR | Development-derived | Specificity | 0.664 | 0.750 | -0.086 (-0.131 to -0.045) | 1125/1061 |
| Test | LR | Development-derived | Brier score | 0.185 | 0.162 | 0.023 (0.006 to 0.042) | 1125/1061 |
| Test | XGBoost | Fixed 0.5 | Sensitivity | 0.362 | 0.258 | 0.105 (0.032 to 0.184) | 1125/1061 |
| Test | XGBoost | Fixed 0.5 | Specificity | 0.976 | 0.983 | -0.007 (-0.022 to 0.005) | 1125/1061 |
| Test | XGBoost | Fixed 0.5 | Brier score | 0.137 | 0.120 | 0.017 (0.000 to 0.035) | 1125/1061 |
| Test | XGBoost | Development-derived | Sensitivity | 0.874 | 0.867 | 0.007 (-0.051 to 0.067) | 1125/1061 |
| Test | XGBoost | Development-derived | Specificity | 0.575 | 0.668 | -0.093 (-0.142 to -0.045) | 1125/1061 |
| Test | XGBoost | Development-derived | Brier score | 0.137 | 0.120 | 0.017 (0.000 to 0.035) | 1125/1061 |
| Test | GPT E1 | Fixed 0.5 | Sensitivity | 0.718 | 0.657 | 0.061 (-0.016 to 0.139) | 1125/1061 |
| Test | GPT E1 | Fixed 0.5 | Specificity | 0.784 | 0.850 | -0.066 (-0.102 to -0.026) | 1125/1061 |
| Test | GPT E1 | Fixed 0.5 | Brier score | 0.167 | 0.146 | 0.021 (0.003 to 0.039) | 1125/1061 |
| Test | GPT E1 | Development-derived | Sensitivity | 0.787 | 0.730 | 0.058 (-0.011 to 0.134) | 1125/1061 |
| Test | GPT E1 | Development-derived | Specificity | 0.699 | 0.775 | -0.076 (-0.121 to -0.034) | 1125/1061 |
| Test | GPT E1 | Development-derived | Brier score | 0.167 | 0.146 | 0.021 (0.003 to 0.039) | 1125/1061 |
| Test | Gemini E2 | Fixed 0.5 | Sensitivity | 0.757 | 0.721 | 0.036 (-0.036 to 0.112) | 1125/1061 |
| Test | Gemini E2 | Fixed 0.5 | Specificity | 0.765 | 0.810 | -0.046 (-0.087 to -0.008) | 1125/1061 |
| Test | Gemini E2 | Fixed 0.5 | Brier score | 0.175 | 0.158 | 0.016 (-0.002 to 0.035) | 1125/1061 |
| Test | Gemini E2 | Development-derived | Sensitivity | 0.757 | 0.721 | 0.036 (-0.036 to 0.112) | 1125/1061 |
| Test | Gemini E2 | Development-derived | Specificity | 0.765 | 0.810 | -0.046 (-0.087 to -0.008) | 1125/1061 |
| Test | Gemini E2 | Development-derived | Brier score | 0.175 | 0.158 | 0.016 (-0.002 to 0.035) | 1125/1061 |
| Holdout | LR | Fixed 0.5 | Sensitivity | 0.822 | 0.737 | 0.084 (0.023 to 0.152) | 1136/1122 |
| Holdout | LR | Fixed 0.5 | Specificity | 0.687 | 0.750 | -0.063 (-0.105 to -0.022) | 1136/1122 |
| Holdout | LR | Fixed 0.5 | Brier score | 0.182 | 0.177 | 0.005 (-0.013 to 0.024) | 1136/1122 |
| Holdout | LR | Development-derived | Sensitivity | 0.841 | 0.770 | 0.072 (0.007 to 0.136) | 1136/1122 |
| Holdout | LR | Development-derived | Specificity | 0.644 | 0.717 | -0.073 (-0.117 to -0.030) | 1136/1122 |
| Holdout | LR | Development-derived | Brier score | 0.182 | 0.177 | 0.005 (-0.013 to 0.024) | 1136/1122 |
| Holdout | XGBoost | Fixed 0.5 | Sensitivity | 0.306 | 0.216 | 0.090 (0.023 to 0.159) | 1136/1122 |
| Holdout | XGBoost | Fixed 0.5 | Specificity | 0.974 | 0.977 | -0.003 (-0.019 to 0.013) | 1136/1122 |
| Holdout | XGBoost | Fixed 0.5 | Brier score | 0.156 | 0.138 | 0.018 (0.000 to 0.037) | 1136/1122 |
| Holdout | XGBoost | Development-derived | Sensitivity | 0.887 | 0.867 | 0.020 (-0.033 to 0.071) | 1136/1122 |
| Holdout | XGBoost | Development-derived | Specificity | 0.580 | 0.578 | 0.002 (-0.049 to 0.048) | 1136/1122 |
| Holdout | XGBoost | Development-derived | Brier score | 0.156 | 0.138 | 0.018 (0.000 to 0.037) | 1136/1122 |
| Holdout | GPT E1 | Fixed 0.5 | Sensitivity | 0.711 | 0.583 | 0.128 (0.055 to 0.210) | 1136/1122 |
| Holdout | GPT E1 | Fixed 0.5 | Specificity | 0.802 | 0.838 | -0.036 (-0.076 to 0.004) | 1136/1122 |
| Holdout | GPT E1 | Fixed 0.5 | Brier score | 0.160 | 0.161 | -0.001 (-0.019 to 0.016) | 1136/1122 |
| Holdout | GPT E1 | Development-derived | Sensitivity | 0.782 | 0.705 | 0.077 (0.012 to 0.151) | 1136/1122 |
| Holdout | GPT E1 | Development-derived | Specificity | 0.720 | 0.750 | -0.030 (-0.073 to 0.011) | 1136/1122 |
| Holdout | GPT E1 | Development-derived | Brier score | 0.160 | 0.161 | -0.001 (-0.019 to 0.016) | 1136/1122 |
| Holdout | Gemini E2 | Fixed 0.5 | Sensitivity | 0.751 | 0.687 | 0.064 (-0.007 to 0.138) | 1136/1122 |
| Holdout | Gemini E2 | Fixed 0.5 | Specificity | 0.789 | 0.802 | -0.013 (-0.052 to 0.027) | 1136/1122 |
| Holdout | Gemini E2 | Fixed 0.5 | Brier score | 0.163 | 0.170 | -0.007 (-0.025 to 0.012) | 1136/1122 |
| Holdout | Gemini E2 | Development-derived | Sensitivity | 0.751 | 0.687 | 0.064 (-0.007 to 0.138) | 1136/1122 |
| Holdout | Gemini E2 | Development-derived | Specificity | 0.789 | 0.802 | -0.013 (-0.052 to 0.027) | 1136/1122 |
| Holdout | Gemini E2 | Development-derived | Brier score | 0.163 | 0.170 | -0.007 (-0.025 to 0.012) | 1136/1122 |

***Note.*** *These analyses are an exploratory subgroup audit, not a comprehensive fairness validation. Age was not dichotomized because the undergraduate age range was narrow, and sample sizes did not support reliable intersectional analysis. Differences use 1,000 bootstrap resamples; output-validity columns are not applicable to structured-data models and are shown according to the source table convention.*

**Table S13.** Expected screening outcomes per 1,000 students and illustrative false-negative:false-positive error-cost scenarios.

**Panel A. Expected outcomes per 1,000 at the observed cohort prevalence**

| **Cohort** | **Condition** | **Strategy** | **Threshold** | **Prevalence** | **TP** | **FP** | **Missed positives** | **TN** | **Further review** |
| --- | --- | --- | --- | --- | --- | --- | --- | --- | --- |
| Test | LR | Fixed 0.5 | 0.500000 | 24.4% | 191.7 | 199.0 | 52.6 | 556.7 | 390.7 |
| Test | LR | Development-derived | 0.466260 | 24.4% | 195.8 | 221.4 | 48.5 | 534.3 | 417.2 |
| Test | XGBoost | Fixed 0.5 | 0.500000 | 24.4% | 77.3 | 15.6 | 167.0 | 740.2 | 92.9 |
| Test | XGBoost | Development-derived | 0.173913 | 24.4% | 212.7 | 285.9 | 31.6 | 469.8 | 498.6 |
| Test | GPT E1 | Fixed 0.5 | 0.500000 | 24.4% | 168.8 | 138.2 | 75.5 | 617.6 | 307.0 |
| Test | GPT E1 | Development-derived | 0.380000 | 24.4% | 186.2 | 198.5 | 58.1 | 557.2 | 384.7 |
| Test | Gemini E2 | Fixed 0.5 | 0.500000 | 24.4% | 181.2 | 160.6 | 63.1 | 595.2 | 341.7 |
| Test | Gemini E2 | Development-derived | 0.500000 | 24.4% | 181.2 | 160.6 | 63.1 | 595.2 | 341.7 |
| Holdout | LR | Fixed 0.5 | 0.500000 | 27.9% | 219.2 | 201.9 | 60.2 | 518.6 | 421.2 |
| Holdout | LR | Development-derived | 0.466260 | 27.9% | 226.3 | 229.4 | 53.1 | 491.1 | 455.7 |
| Holdout | XGBoost | Fixed 0.5 | 0.500000 | 27.9% | 74.4 | 17.3 | 205.0 | 703.3 | 91.7 |
| Holdout | XGBoost | Development-derived | 0.173913 | 27.9% | 245.3 | 303.4 | 34.1 | 417.2 | 548.7 |
| Holdout | GPT E1 | Fixed 0.5 | 0.500000 | 27.9% | 182.9 | 129.3 | 96.5 | 591.2 | 312.2 |
| Holdout | GPT E1 | Development-derived | 0.380000 | 27.9% | 209.0 | 190.4 | 70.4 | 530.1 | 399.5 |
| Holdout | Gemini E2 | Fixed 0.5 | 0.500000 | 27.9% | 201.9 | 147.0 | 77.5 | 573.5 | 349.0 |
| Holdout | Gemini E2 | Development-derived | 0.500000 | 27.9% | 201.9 | 147.0 | 77.5 | 573.5 | 349.0 |

**Panel B. Standardized expected loss by illustrative FN:FP cost ratio**

| **Cohort** | **Condition** | **Strategy** | **Loss 1:1** | **Loss 2:1** | **Loss 5:1** | **Loss 10:1** |
| --- | --- | --- | --- | --- | --- | --- |
| Test | LR | Fixed 0.5 | 251.6 | 304.2 | 462.0 | 725.1 |
| Test | LR | Development-derived | 269.9 | 318.4 | 463.9 | 706.3 |
| Test | XGBoost | Fixed 0.5 | 182.5 | 349.5 | 850.4 | 1685.3 |
| Test | XGBoost | Development-derived | 317.5 | 349.0 | 443.7 | 601.6 |
| Test | GPT E1 | Fixed 0.5 | 213.6 | 289.1 | 515.6 | 893.0 |
| Test | GPT E1 | Development-derived | 256.6 | 314.7 | 489.0 | 779.5 |
| Test | Gemini E2 | Fixed 0.5 | 223.7 | 286.8 | 476.2 | 791.9 |
| Test | Gemini E2 | Development-derived | 223.7 | 286.8 | 476.2 | 791.9 |
| Holdout | LR | Fixed 0.5 | 262.2 | 322.4 | 503.1 | 804.3 |
| Holdout | LR | Development-derived | 282.6 | 335.7 | 495.1 | 760.9 |
| Holdout | XGBoost | Fixed 0.5 | 222.3 | 427.4 | 1042.5 | 2067.8 |
| Holdout | XGBoost | Development-derived | 337.5 | 371.6 | 473.9 | 644.4 |
| Holdout | GPT E1 | Fixed 0.5 | 225.9 | 322.4 | 612.0 | 1094.8 |
| Holdout | GPT E1 | Development-derived | 260.9 | 331.3 | 542.5 | 894.6 |
| Holdout | Gemini E2 | Fixed 0.5 | 224.5 | 302.0 | 534.5 | 922.1 |
| Holdout | Gemini E2 | Development-derived | 224.5 | 302.0 | 534.5 | 922.1 |

***Note.*** *TP, true positives; FP, false positives; TN, true negatives. Missed positives are false negatives. Further review equals TP+FP. Standardized expected loss is (FN cost ratio x missed positives)+false positives per 1,000. Cost ratios are illustrative scenarios rather than validated clinical utilities or monetary costs.*

**Figure S1.** Combined-sample Spearman correlation matrix for continuous SDS standard score, SAS, CFQ, AAQ-II, the eight eligible non-depression SCL-90 symptom dimensions, and the additional-items score. Cells are colored by Spearman rho on the common scale from -1 to 1; diagonal entries equal 1. The matrix is descriptive of shared questionnaire constructs and does not imply causal relationships. SDS-specific correlations, cohort-specific estimates, sample sizes, and bootstrap 95% confidence intervals are reported in Supplementary Table S8.

**Figure S2.** Grouped scale-ablation effects on macro-F1, AUROC, and Brier score. Points show the ablated-minus-full-model difference and horizontal bars show paired bootstrap 95% confidence intervals. Blue estimates are LR and XGBoost leave-one-group-out analyses on Test and Holdout; green estimates are exploratory GPT E1 and Gemini E2 input deletions in the prevalence-representative 400-case Holdout subset at temperature=0. For Brier score, positive values indicate worse probability accuracy. LLM target profiles and few-shot examples had the same group removed. Full and single-group results are in Supplementary Table S8. The larger demographics-only versus full-model differences are reported separately in Supplementary Table S8, Panel E, and are omitted here to preserve the visual scale of the grouped questionnaire-ablation effects.

**Figure S3.** Absolute standardized mean differences across Development, Test, and Holdout. Rows are demographic, questionnaire, and SDS screen-positive variables; columns are the three pairwise cohort comparisons. Cell labels give absolute SMD values and color intensity increases with shift magnitude. The large age differences reflect the prespecified grade-cohort design. Complete descriptive distributions, signed SMDs, Kolmogorov-Smirnov statistics for continuous variables, Wilson intervals for binary proportions, and absolute prevalence differences are reported in Supplementary Table S9.

**Figure S4.** Calibration and probability distributions on Test for LR, XGBoost, GPT E1, and Gemini E2. For each condition, the left panel plots observed SDS screen-positive rate against mean predicted probability in equal-frequency bins; the dashed line is ideal calibration and point labels show the bin sample size. The right panel shows predicted-probability histograms stratified by observed negative and positive status without kernel smoothing. LLM panels use operationally valid raw model-reported probabilities; no post-hoc LLM calibration was fitted. Calibration intercepts, slopes, Brier scores, ECE values, confidence intervals, and model-native/common-valid denominators are in Supplementary Table S10.

**Figure S5.** Calibration and probability distributions on the independent within-institution Holdout grade cohort for LR, XGBoost, GPT E1, and Gemini E2. For each condition, the left panel plots observed SDS screen-positive rate against mean predicted probability in equal-frequency bins; the dashed line is ideal calibration and point labels show the bin sample size. The right panel shows predicted-probability histograms stratified by observed negative and positive status. LLM panels use operationally valid raw model-reported probabilities; no post-hoc LLM calibration was fitted. Holdout is not geographic, temporal, or clinical external validation. Numerical calibration results are in Supplementary Table S10.

**Figure S6.** Expected calibration error sensitivity analysis on Test and Holdout. Lines show ECE for LR, XGBoost, GPT E1, and Gemini E2 under equal-width (solid) and equal-frequency (dashed) binning with 5, 10, 15, or 20 bins. The analysis demonstrates the dependence of ECE magnitude on binning specification while preserving the broad between-model pattern. LLM estimates use operationally valid probabilities. Supplementary Table S10 reports the 10-bin equal-frequency ECE used for its tabular summary, together with bootstrap confidence intervals, calibration intercepts and slopes, Brier scores, and denominator analyses. The prespecified primary ECE used 10 equal-width bins.

**Figure S7.** Threshold robustness for LR, XGBoost, GPT E1, and Gemini E2 on Test (top row) and Holdout (bottom row). Curves show sensitivity, specificity, and macro-F1 over decision thresholds from 0.05 to 0.95. The vertical dashed line marks the prespecified 0.5 rule; colored dots mark each model-specific Youden threshold selected only in Development-validation and applied unchanged to both evaluation cohorts. Step-like curves reflect discrete predicted probabilities, particularly after isotonic calibration. Numerical 0.5, Development-derived, and fixed-sensitivity/fixed-specificity operating-point results are reported in Supplementary Table S6.
